# Lactam Truncation Yields a Dihydroquinazolinone Scaffold with Potent Antimalarial Activity that Targets PfATP4

**DOI:** 10.1002/cmdc.202400549

**Published:** 2024-10-29

**Authors:** Trent D. Ashton, Petar P. S. Calic, Madeline G. Dans, Zi Kang Ooi, Qingmiao Zhou, Katie Loi, Kate E. Jarman, Josephine Palandri, Deyun Qiu, Adele M. Lehane, Bikash Maity, Nirupam De, Mufuliat T. Famodimu, Michael J. Delves, Emma Y. Mao, Maria R. Gancheva, Danny W. Wilson, Mrittika Chowdury, Tania F. de Koning‐Ward, Delphine Baud, Stephen Brand, Paul F. Jackson, Alan F. Cowman, Brad E. Sleebs

**Affiliations:** ^1^ Walter and Eliza Hall Institute of Medical Research Parkville 3052 Victoria Australia; ^2^ Department of Medical Biology University of Melbourne Parkville 3010 Victoria Australia; ^3^ Research School of Biology Australian National University Canberra 2601 Australia; ^4^ TCG Lifesciences Kolkata West Bengal 700091 India; ^5^ Department of Infection Biology London School of Hygiene and Tropical Medicine London WC1E 7HT UK; ^6^ Research Centre for Infectious Diseases School of Biological Sciences University of Adelaide Adelaide 5005 Australia; ^7^ School of Medicine Deakin University Waurn Ponds Victoria 3216 Australia; ^8^ Institute for Mental and Physical Health and Clinical Translation Deakin University Geelong Victoria 3216 Australia; ^9^ MMV Medicines for Malaria Venture, ICC Route de Pré-Bois 20 1215 Geneva Switzerland; ^10^ Emerging Science & Innovation Discovery Sciences, Janssen R&D LLC La Jolla California 92121 USA

**Keywords:** Antimalarial, Malaria, PfATP4, *Plasmodium*, Quinazolinone

## Abstract

The emergence of resistance against current antimalarial treatments has necessitated the need for the development of novel antimalarial chemotypes. Toward this goal, we recently optimised the antimalarial activity of the dihydroquinazolinone scaffold and showed it targeted PfATP4. Here, we deconstruct the lactam moiety of the tricyclic dihydroquinazolinone scaffold and investigate the structure‐activity relationship of the truncated scaffold. It was shown that SAR between scaffolds was largely transferrable and generated analogues with potent asexual stage activity. Evaluation of the truncated analogues against PfATP4 mutant drug‐resistant parasite strains and in assays measuring PfATP4‐associated ATPase activity demonstrated retention of PfATP4 as the molecular target. Analogues exhibited activity against both male and female gametes and multidrug resistant parasites. Limited efficacy of analogues in a *P. berghei* asexual stage mouse model was attributed to their moderate metabolic stability and low aqueous stability. Further development is required to address these attributes toward the potential use of the dihydroquinazolinone class in a curative and transmission blocking combination antimalarial therapy.

## Graphical abstract

Truncation of the dihydroquinazolinone scaffold produced analogues with potent antimalarial activity against the asexual blood and transmission stages of the malaria parasite's lifecycle by targeting PfATP4. Enhancing the metabolic stability and aqueous solubility remains a challenge to achieve curative efficacy in a mouse model to demonstrate the potential of the scaffold in a curative or transmission blocking therapy.

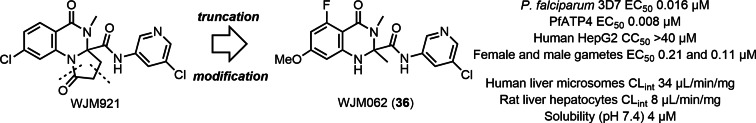

## Introduction

Malaria is a devastating disease that is caused by five species of the *Plasmodium* parasite. *P. falciparum* is the most prevalent in sub‐Saharan Africa and causes approximately 95 % of malaria related deaths worldwide.^[1]^ *P. vivax* is primarily found in Southeast Asia and the Americas and is responsible for the relapse of the disease after drug treatment due to the dormant hypnozoite stage. *P. ovale*, *P. malariae* and *P. knowlesi* are endemic to South‐East Asia and although they rarely cause death, worryingly, the prevalence of infections by these species is increasing.[^2^, ^3^]

Malaria infections are curtailed by using insecticide treated bed nets and chemo‐preventative antimalarials such as doxycycline and atovaquone‐proguanil (Malarone). RTS,S /AS01 is the only approved vaccine to prevent malaria but is only 30–40 % effective,^[4]^ however new adjunct matrix version (R21/Matrix‐M) of this vaccine has significantly improved effectiveness in clinical trials.^[5]^ Treatment of malaria in the past has been heavily reliant on quinoline and the related phenanthrene‐based antimalarials. Although these antimalarials have largely been effective in the past, the over‐reliance on these drug classes has resulted in widespread resistance limiting their current use.^[6]^ Artemisinin‐based combination therapies are the current front‐line antimalarial therapies; however, resistance has emerged in South‐East Asia^[7]^ and more recently in Sub‐Saharan Africa.^[8]^ This highlights the need for the discovery of novel chemotypes that are devoid of the same resistance profile as current drugs and ideally target multiple stages of the parasite's lifecycle. In the last 20 years, promisingly new antimalarial chemotypes have been developed and entered human clinical trials.^[9]^ Even so, resistance‐conferring mutations have been detected in clinical trials on these candidates, highlighting the need to continually discover new antimalarial entities for development.

**Figure 1:**
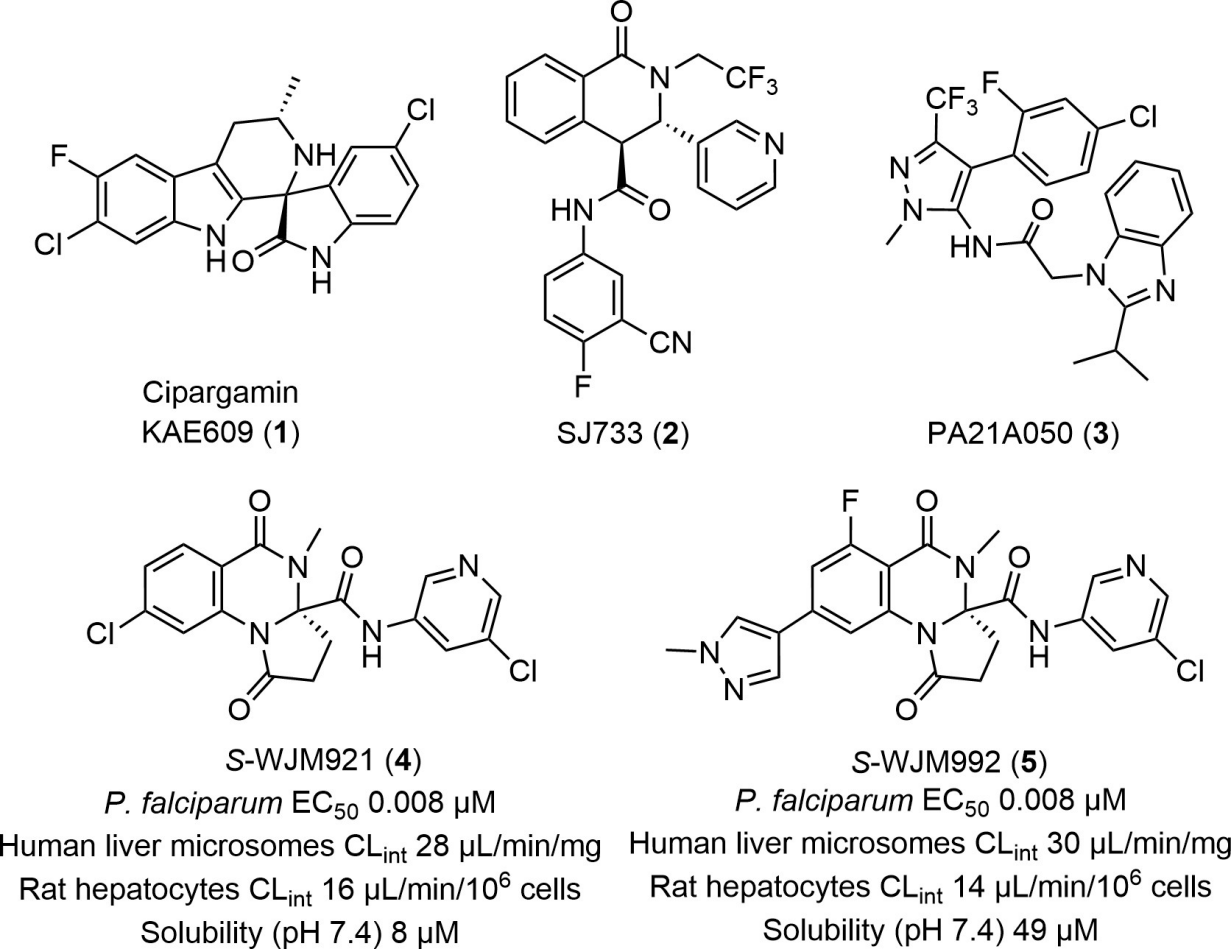
The dihydroquinazolinone and known PfATP4 targeted antimalarials.

To uncover new antimalarial chemotypes, we recently screened the Janssen Jumpstarter library against the *P. falciparum* 3D7 asexual stage parasite and uncovered several hit structural classes for further development.[^10^, ^11^, ^12^, ^13^, ^14^, ^15^] One of the hit classes discovered was from the dihydroquinazolinone structural class.^[10]^ In previous research, we established the structure activity relationship and optimized the scaffold producing the early frontrunner compound WJM921 **4** (Figure 1). WJM921 **4** exhibited potent activity against the *P. falciparum* asexual stage parasites *in vitro* but had limited aqueous solubility and moderate metabolic stability *in vitro*.^[10]^ These *in vitro* ADME properties were then further enhanced producing the frontrunner analogue, WJM992 **5**, which at a 25 mg/kg oral dose cleared parasitemia in a 4‐day *P. falciparum* humanised mouse model, although recrudescence of parasitemia was detected after day 4.^[16]^

Using forward genetics we previously established that the dihydroquinazolinone chemotype targets PfATP4.^[10]^ PfATP4 is a clinically validated antimalarial drug target with several inhibitors that are currently in pre‐clinical or clinical development, including KAE609 (cipargamin) **1**,^[17]^ SJ733 **2**,^[18]^ and PA21 A050 **3**.^[19]^ PfATP4 is a P‐type ATPase ion transporter and is essential for regulating cytosolic levels of Na^+^.^[20]^ PfATP4 maintains Na^+^ homeostasis by extruding Na^+^ from the parasite cytosol while importing H^+^.^[21]^ PfATP4 inhibitors, including WJM921 **4** cause increased cytosolic Na^+^ and pH resulting in the death of both asexual and sexual (transmission) stage parasites.[^10^, ^16^] A limitation of PfATP4 inhibitors is the relatively quick onset of resistance. Notably, KAE609 **1**, SJ733 **2**, and PA21A050 **3** have decreased activity against parasites with a PfATP4^G358S^ mutation^[22]^ originally detected in patients treated with KAE609 **1** in clinical trials.^[23]^ Dihydroquinazolinone analogues have slightly reduced (1.5 to 2‐fold) activity against the PfATP4^G358S^ parasite strain and thus there is continued interest in further optimisation of this structural class.

One region of the dihydroquinazolinone scaffold that remains unexplored is the N1‐C2 lactam motif. Here we investigate the necessity of the lactam motif by truncating the lactam motif to assess the effect on antimalarial activity and *in vitro* ADME properties. We also evaluated truncated analogues against mutant PfATP4 parasite strains and in an assay that measures PfATP4 activity to assess whether modifications made to the scaffold altered their mechanism of action. Finally, we assess the activity of the truncated analogues against multidrug‐resistant parasites, *P. knowlesi* parasites to assess species differentiation, sexual stage parasites to test transmission blocking potential, and finally, determine efficacy in a *P. berghei* mouse model.

## Results and Discussion

### Synthesis

**Scheme 1:**
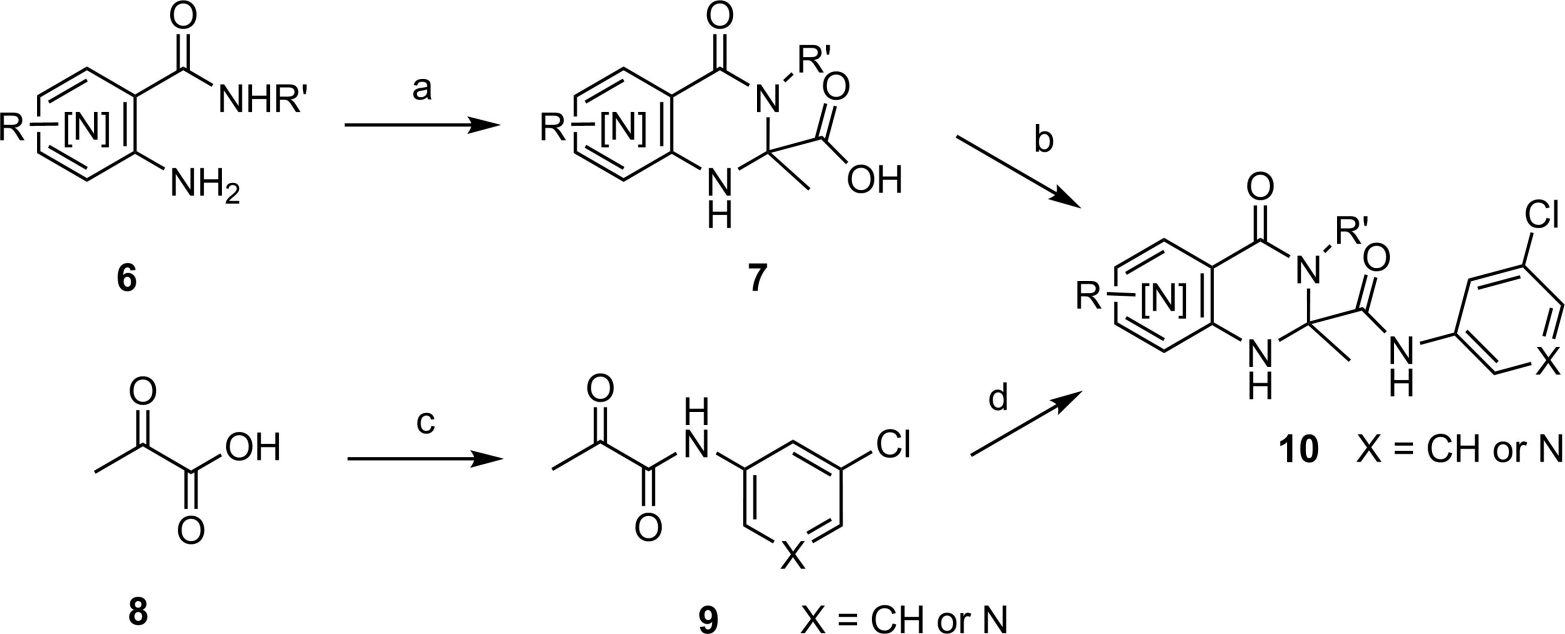
General synthetic pathway to access dihydroquinazolinone analogues. *Reagents and conditions*: a) pyruvic acid, AcOH, 135 °C; (b) R“NH_2_, HATU, DIPEA, DMF; c) i) SOCl_2_, DMF; ii) 3‐chloro aniline or 3‐chloro‐5‐amino pyridine, DMF. d) **6**, AcOH, 135 °C.

Two general pathways were undertaken for the synthesis of analogues (Scheme 1). In the first synthetic route, an anthranilamide synthon **6** was subjected to a cyclo‐condensation reaction using pyruvic acid at an elevated temperature to give the dihydroquinazolinone 2‐carboxylic acid **7**. Subsequent carboxamide formation using HATU as the coupling agent gave the carboxamide product **10**. The alternative pathway started with by conversion pyruvic acid **8** to the acid chloride and addition of the appropriate amino arene to give the α‐keto carboxamide **9**. The α‐keto carboxamide **9** was then used in a cyclo‐condensation reaction with a suitable anthranilamide **6** to yield the dihydroquinazolinone carboxamide **10**. Due to synthetic tractability, all analogues produced for this study were achiral at the 2‐carbon.

### Structure Activity Relationship

**Figure 2:**
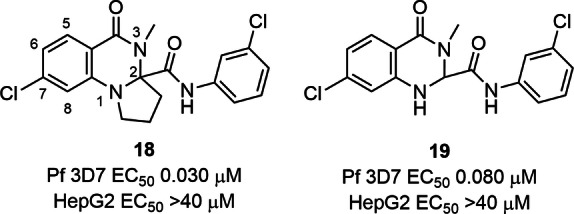
Activity of N1 and C2 iterations on the dihydroquinazolinone scaffold.

We began the SAR study by investigating the importance of the N1‐C2 lactam moiety by removing the carbonyl functionality (**18**) and found an approximate 4‐fold reduction in antimalarial activity (EC_50_ 0.030 μM) (Figure 2) compared to WJM921 **4** (Figure 1). Deletion of the N1‐C1 lactam (**19**) resulted in a further 2.5‐fold decrease in activity (EC_50_ 0.080 μM) signifying that either C2 or N1 substitution was important for activity. Adding a methyl group at C2 with no N1 substitution (**11**) gave a 4‐fold increase in activity (EC_50_ 0.018 μM) (Table 1). Confoundingly, the addition of a methyl group at N1 (**12**) significantly reduced antimalarial activity (EC_50_ 0.115 μM), while N‐ethyl substitution (**13**) also reduced activity by 4‐fold (EC_50_ 0.053 μM) relative to the unsubstituted analogue (**11**). This SAR was largely transferable to analogues with a 3‐endocyclic nitrogen on the exocyclic ring, although there was no difference in activity (EC_50_ 0.033 and 0.047 μM) between N1 methyl substituted and the N1 unsubstituted analogues **14** and **15**.

**Table 1:** Biological activities of bicyclic analogues.

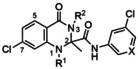										
Cmpnd	R^1^	R^2^	X	Pf 3D7 EC_50_ (SD) μM^[a]^	HepG2 CC_50_ (SD) μM^[b]^	PSA (Å^2^)	cLogD^[c]^ or eLogD^[d]^	HLM CL_int_ (μL/min/mg)	Rat Hep CL_int_ (μL/min/10^6^ cells)	Solubility (pH 7.4) (μM)
**11**	H	Me	CH	0.018 (0.005)	>40	61	3.2^[c]^	–	–	–
**12**	Me	Me	CH	0.115 (0.005)	>40	53	3.9^[d]^	122	124	2.6
**13**	Et	Me	CH	0.053 (0.024)	>40	53	3.9 ^c^	–	–	–
**14**	H	Me	N	0.033 (0.005)	>40	74	3.2^[d]^	60	25	5.2
**15**	Me	Me	N	0.047 (0.010)	>40	66	2.4^[c]^	–	–	–
**16**	H	CyPr	N	0.027 (0.005)	>40	74	3.3^[d]^	172	33	7.0
**17**	Me	CyPr	N	0.023 (0.006)	>40	65	3.2^[d]^	267	47	28

[a] EC_50_ data represent the mean and SD for 3 independent experiments measuring LDH activity of *P. falciparum* (Pf) 3D7 parasites at 72 h. [b] CC_50_ data represent the mean and SD for 4 replicate experiments measuring growth inhibition of HepG2 cells at 48 h using Cell Titre Glo. [c] *in silico* calculation using Domatics software. [d] shake‐flask method. HLM=human liver microsomes; Hep=hepatocytes.

*The in vitro* ADME of the truncated analogues **12** and **14** was then assessed. This analysis revealed both analogues had relatively low aqueous solubility (<5.2 μM), and that the N1‐methylated analogue **12** had low metabolic stability (human liver microsome CL_int_ 122 μL/min/mg and rat hepatocyte CL_int_ 124 μL/min/10^6^ cells), while the metabolic stability of analogue **14** without N1‐substitution was moderately improved (human liver microsome CL_int_ 60 μL/min/mg and rat hepatocyte CL_int_ 25 μL/min/10^6^ cells) (Table 1). A strategy to overcome metabolism is to replace substituents at predicted metabolically liable sites. To overcome N3‐demethylation we opted to replace the N‐methyl group with a cyclopropyl group, known to be the only other substituent to be tolerated in the N3‐position from previous work.^[10]^ Accordingly, the antimalarial activity (EC_50_ 0.027 and −0.023 μM) of the analogues **16** and **17** with an N3‐cyclopropyl group and with and without N1‐methyl group was comparable to the N3‐methyl counterparts (**14** and **15**). The inclusion of the N3‐cyclopropyl group did not rectify *in vitro* metabolism. Notably, analogue **17** with an N1‐methyl group had improved aqueous solubility (28 μM versus 7 μM) but had significantly lower metabolic stability (human liver microsome CL_int_ 172 versus 267 μL/min/mg) compared with the N1‐unsubstituted variant **16**.

We next considered whether a polar substituent could be installed in the N1‐position via an ethyl carboxamide linker to improve solubility and possibly reduce the overall lipophilicity that could be contributing to low metabolic stability. It was shown that the inclusion of these groups (**20–23**) was tolerated (EC_50_ 0.023–0.072 μM) with comparable antimalarial activity to the N1‐methyl equivalent **15** (Table 2). Surprisingly, the synthetic precursor **24** with a N‐Cbz protection group on the piperazine was also tolerated (EC_50_ 0.080 μM) suggesting the N1‐functional group was orientated toward solvent space when the scaffold is engaged with its molecular target PfATP4. The attachment of a polar group (**22** and **21**) gave a marked improvement in aqueous solubility (41 and 178 μM), although the solubility of the N‐methyl piperazine analogue **23** was unexpectedly modest (9.6 μM). The N1‐polar modification was found to significantly enhance rat hepatocyte stability (CL_int_ 7.7–23 μL/min/10^6^ cells) although there was no improvement in human microsome stability (CL_int_ 94–155 μL/min/mg).

**Table 2:** Biological activities of analogues with N1‐alkyl polar groups.

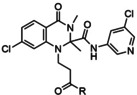								
Cmpnd	R	Pf 3D7 EC_50_ (SD) μM^[a]^	HepG2 CC_50_ (SD) μM^[b]^	PSA (Å^2^)	cLogD^[c]^ or eLogD^[d]^	HLM CL_int_ (μL/min/mg)	Rat Hep CL_int_ (μL/min/10^6^ cells)	Solubility (pH 7.4) (μM)
**20**	N,N‐dimethyl	0.058 (0.008)	>40	94	1.7^[c]^	–	–	–
**21**	N,N‐morpholine	0.072 (0.020)	>40	104	2.9^[d]^	95	23	41
**22**	N,N‐piperazine	0.023 (0.005)	>40	107	1.7^[d]^	94	7.7	178
**23**	N,N‐piperazine(N−Me)	0.024 (0.006)	>40	98	2.8^[d]^	155	16	9.6
**24**	N,N‐piperazine(N‐Cbz)	0.080 (0.041)	>40	124	3.1^[c]^	–	–	–

[a] EC_50_ data represent the mean and SD for 3 independent experiments measuring LDH activity of *P. falciparum* (Pf) 3D7 parasites at 72 h. [b] CC_50_ data represent the mean and SD for 4 replicate experiments measuring growth inhibition of HepG2 cells at 48 h using Cell Titre Glo. [c] *in silico* calculation using Domatics software. [d] shake‐flask method. HLM=human liver microsomes; Hep=hepatocytes.

To further enhance solubility and overcome the metabolic N1‐demethylation, we considered replacing the carboxamide motif on the dihydroquinazolinone framework with a suitable bioisostere that was synthetically viable. It was shown that imidazole was a suitable bioisostere as the analogue **27** showed equipotent antimalarial activity (EC_50_ 0.054 μM) relative to **14** (Table 3). Confoundingly, the dihydroimidazole analogue **28** was inactive (EC_50_ >10 μM). The aqueous solubility of both **27** and **28** was significantly improved (84 and >200 μM) attributed to the inclusion of the imidazole and dihydroimidazole systems. There was no improvement in metabolism (human liver microsome CL_int_ 110 μL/min/mg and rat hepatocyte CL_int_ 59 μL/min/10^6^ cells) of the imidazole analogue **27** compared to **14**, and while the metabolism of dihydroimidazole analogue **28** was improved (human liver microsome CL_int_ 37 μL/min/mg and rat hepatocyte CL_int_ 8.8 μL/min/10^6^ cells), **28** was devoid of antimalarial activity.

**Table 3:** Biological activities of carboxamide bioisostere analogues.

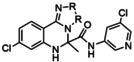								
Cmpnd	R	Pf 3D7 EC_50_ (SD) μM^[a]^	HepG2 CC_50_ (SD) μM^[b]^	PSA (Å^2^)	eLogD^[c]^	HLM CL_int_ (μL/min/mg)	Rat Hep CL_int_ (μL/min/10^6^ cells)	Solubility (pH 7.4) (μM)
**27**	CH	0.054 (0.006)	>40	72	2.7	110	59	84
**28**	CH_2_	>1.0	nd	70	2.5	37	8.8	>200

[a] EC_50_ data represent the mean and SD for 3 independent experiments measuring LDH activity of *P. falciparum* (Pf) 3D7 parasites at 72 h. [b] CC_50_ data represent the mean and SD for 4 replicate experiments measuring growth inhibition of HepG2 cells at 48 h using Cell Titre Glo. [c] shake‐flask method. HLM=human liver microsomes; Hep=hepatocytes.

**Figure 3:**
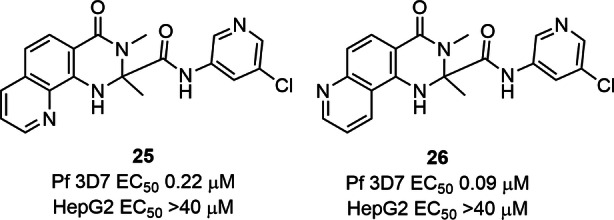
Biological activity of tricyclic analogues.

The 8‐position on the dihydroquinazolinone scaffold is known to be tolerant of a variety of substituents.[^10^, ^16^] We next reasoned that the 8‐position may be suitable for enhancing physicochemical properties, and therefore we formulated several iterations to probe this hypothesis. The first modification was the incorporation of a pyridyl ring system encompassing the 8‐ and 9‐positions of the dihydroquinazolinone system. Two pyridyl variations were synthetically tractable from commercially available building blocks to give analogues **25** and **26** (Figure 3). The antimalarial activity of the 9‐N pyridyl analogue **25** was 6‐fold less active (EC_50_ 0.22 μM) than **14**, while the 8‐N pyridyl analogue **26** 2.5‐fold less active (EC_50_ 0.09 μM). This activity was thought to be less than desirable, and therefore this fused ring system was not further pursued.

The next modification attempted was the incorporation of polar groups at the 8‐position. The polar groups were selected based on their antimalarial activity and *in vitro* ADME parameters from our previous study.^[16]^ It was found that 3‐pyridyl substitution (**29** and **30**) was tolerated in the 8‐position but was 4‐fold less potent (EC_50_ 0.148 and 0.143 μM), relative to **14**. Modification with 3‐pyrazole(N−Me) (**31**) was detrimental to antimalarial activity (EC_50_ 0.808 μM), but the 4‐pyrazole(N−Me) regioisomer (**32**) was preferred, but 6‐fold less potent (EC_50_ 0.198 μM) than **14**.

The N‐pyrazole variant (**33**) exhibited the most potent antiparasitic activity (EC_50_ 0.065 μM) of this analogue cohort. The SAR trends observed in the 8‐position are relatively consistent with that found for the scaffold with the lactam,^[16]^ but the lactam scaffold overall was more tolerant of variation and generally, 2‐ to 4‐fold more active. Notably, introduction of the 3‐pyrazole(N−Me) and the N‐pyrazole heterocycles (**32** and **33**) modestly improved the *in vitro* metabolic stability (human liver microsome CL_int_ 34 and 37 μL/min/mg and rat hepatocyte CL_int_ 5 and 14 μL/min/10^6^ cells), but surprisingly did not improve the aqueous solubility at pH 7.4 (7.0 and 2.6 μM).

The methoxy group was next canvased for inclusion in the 8‐position because it showed promising activity in our prior study.^[10]^ Accordingly, the 8‐methoxy substituent on the truncated scaffold (**34**) showed a similar level of potency (EC_50_ 0.074 μM) as the 8‐chloro substituent (**14**). The metabolic stability (human liver microsome CL_int_ 35 μL/min/mg and rat hepatocyte CL_int_ 16 μL/min/10^6^ cells) and the aqueous solubility (13.5 μM) were both slightly enhanced with the inclusion of the 8‐methoxy group (**34**). To further enhance properties a 6‐endocyclic nitrogen was combined with the 8‐methoxy substituent (**35**), although this led to a marked decrease in antimalarial activity (EC_50_ 0.458 μM). The addition of a 6‐fluoro with the 8‐methoxy group (WJM062, **36**) led to a 2‐fold improvement in antiparasitic activity (EC_50_ 0.021 μM). A marginal improvement in metabolic stability (human liver microsome CL_int_ 34 μL/min/mg and rat hepatocyte CL_int_ 8 μL/min/10^6^ cells) was also observed with **36**, but the addition of the 6‐fluoro increased lipophilicity resulting in low aqueous solubility (4.0 μM).

A selection of the analogues designed and synthesised including **14**, **33**, **36** were deemed to have suitable antiparasitic activity and *in vitro* ADME properties for further profiling in malaria and PfATP4 phenotypic assays. It was also noted that throughout the SAR study, none of the truncated analogues exhibited cytotoxicity toward human HepG2 cells (CC_50_ >40 μM) (Tables 1 −4; Figures 2 and 3) demonstrating the selectivity of the dihydroquinazolinone antimalarial chemotype.

**Table 4:** Biological activities of 8‐substituted analogues.

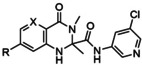									
Cmpnd	R	X	Pf 3D7 EC_50_ (SD) μM^[a]^	HepG2 CC_50_ (SD) μM^[b]^	PSA (Å^2^)	cLogD^[c]^ or eLogD^[d]^	HLM CL_int_ (μL/min/mg)	Rat Hep CL_int_ (μL/min/10^6^ cells)	Solubility (pH 7.4) (μM)
**14**	Cl	CH	0.033 (0.005)	>40	74	3.2^[d]^	60	25	5.2
**29**	3‐pyridyl	CH	0.148 (0.038)	>40	87	1.8^[c]^	–	–	–
**30**	3‐pyridyl(4‐OMe)	CH	0.143 (0.021)	>40	96	2.3^[c]^	–	–	–
**31**	3‐pyrazole(N−Me)	CH	0.808 (0.115)	>40	92	2.0^[c]^	–	–	–
**32**	4‐pyrazole(N−Me)	CH	0.198 (0.067)	>40	92	2.9^[d]^	34	5	7.0
**33**	N‐pyrazole	CH	0.065 (0.009)	>40	92	3.2^[d]^	37	14	2.6
**34**	OMe	CH	0.074 (0.001)	nd	84	2.7	35	16	13.5
**35**	OMe	N	0.458 (0.057)	>40	96	0.6^[c]^	–	–	–
**36** (WJM062)	OMe	CF	0.021 (0.007)	>40	84	2.6^[d]^	34	8	4.0

[a] EC_50_ data represent the mean and SD for 3 independent experiments measuring LDH activity of *P. falciparum* (Pf) 3D7 parasites at 72 h. [b] CC_50_ data represent the mean and SD for 4 replicate experiments measuring growth inhibition of HepG2 cells at 48 h using Cell Titre Glo. [c] *in silico* calculation using Domatics software. [d] shake‐flask method. HLM=human liver microsomes; Hep=hepatocytes.

### Activity Against Parasites with Mutations in PfATP4

To determine if the lactam truncation and modification of the dihydroquinazolinone scaffold adversely affected activity against the PfATP4 target, we evaluated representative analogues against parasite strains with mutations in PfATP4.

We previously generated three ‘compound 49’ (an analogue related to WJM921, Figure 1) resistant parasite populations.^[10]^ Subsequently, whole genome sequencing on each population was undertaken and uncovered an F156L mutation, a D425E mutation, and a 2.8‐fold amplification in PfATP4. It was also found that KAE609 and SJ733 show slightly reduced activity against ‘compound 49’ resistant strains.^[10]^ We evaluated selected truncated analogues **11**, **14**, **33**, and **36** (WJM062) against these PfATP4 mutant strains and found all these analogues were approximately 1.5‐ to 2‐fold less active against these strains (EC_50_ 0.036–0.126 μM) than against the Dd2 parental strain (EC_50_ 0.021–0.073 μM) (Table 5 and Figure S2). This fold‐change in activity was approximately the same for the related analogues, WJM921 **4** and WJM992 **5**,[^10^, ^16^] suggesting that PfATP4 is the molecular target of the truncated analogues.

**Table 5:** Evaluation of selected compounds against ‘compound 49’ drug‐ resistant *P. falciparum* strains.^[a]^

Cmpnd	Dd2^parental^ EC_50_ (SD) μM	Dd2^F156L^ EC_50_ (SD) μM	Dd2^D425E^ EC_50_ (SD) μM	Dd2^2.8 CNV^ EC_50_ (SD) μM
**11**	0.021 (0.005)	0.041 (0.031)	0.036 (0.001)	0.040 (0.021)
**14**	0.050 (0.011)	0.089 (0.031)	0.055 (0.004)	0.061 (0.006)
**33**	0.073 (0.008)	0.125 (0.009)	0.126 (0.023)	0.124 (0.031)
**36** (WJM062)	0.032 (0.004)	0.047 (0.002)	0.043 (0.014)	0.056 (0.030)
KAE609	0.002	0.004 (<0.001)	0.002 (<0.001)	0.003 (<0.001)
SJ733	0.068	0.135 (0.016)	0.080 (0.002)	0.129 (0.014)

[a] EC_50_ values are an average of 3 independent experiments in replicate against Dd2 parental and ‘compound 49’ resistant strains^[10]^ measuring LDH at 72 h. See Figure S2 for dose response curves.

WJM921 **4** and WJM992 **5** derived analogues show reduced activity against the laboratory‐derived PfATP4^I398F, P990R, D1247Y^ drug‐resistant strain, and a slight reduction in activity against the clinically relevant PfATP4^G358S^ drug‐resistant strain.[^10^, ^16^] The clinical candidates, KAE609 (**1**), SJ733 (**2**) and PA21 A050 (**3**) show significantly reduced activity against the PfATP4^G358S^ strain and slightly reduced activity against the PfATP4^I398F, P990R, D1247Y^ strain compared to the parental Dd2 parasite line.[^17^, ^18^, ^19^, ^24^] It was shown that analogues **11**, **14**, and **36** (WJM062) were between 1.5‐ to 2‐ fold less potent (EC_50_ 0.041–0.061 μM) against the PfATP4^G358S^ strain while the analogue **33** was 7‐fold less potent (EC_50_ 0.537 μM) relative to the Dd2 parental line. Analogues **11**, **14**, **33**, and **36** showed between 3‐ to 7‐fold reduced activity (EC_50_ 0.021–0.073 μM) against the PfATP4^I398F, P990R, D1247Y^ drug‐resistant strain (Table 6 and Figure S3) compared to the Dd2 line activity (EC_50_ 0.100–0.584 μM). The resistance profile exhibited by the truncated analogues is akin to WJM921 **4** and WJM992 **5** derived analogues,[^10^, ^16^] and provides further evidence the truncated analogues target PfATP4.

**Table 6:** Evaluation of selected compounds against PfATP4 drug resistant *P. falciparum* strains.^[a]^

Cmpnd	Dd2^parental^ EC_50_ (SD) μM	Dd2^triple^ EC_50_ (SD) μM	Dd2^G358S^ EC_50_ (SD) μM
11	0.021 (0.005)	0.140 (0.057)	0.044 (0.002)
14	0.050 (0.011)	0.160 (0.058)	0.061 (0.004)
33	0.073 (0.008)	0.584 (0.329)	0.537 (0.201)
36 (WJM062)	0.032 (0.004)	0.100 (0.045)	0.041 (0.003)
KAE609^[b]^	0.002	0.009	1.34
SJ733^[b]^	0.068	0.085	>11

[a] EC_50_ values are an average of 3 independent experiments against Dd2 parental, Dd2 PfATP4^triple^ (I398F, P990R, D1247Y)^[17]^ and PfATP4^G358S^ resistant strains^[18]^ measuring LDH at 72 h. See Figure S3 for dose response curves. [b] data taken from Ashton, Dans *et al*.^[10]^

### Activity Against PfATP4

**Figure 4:**
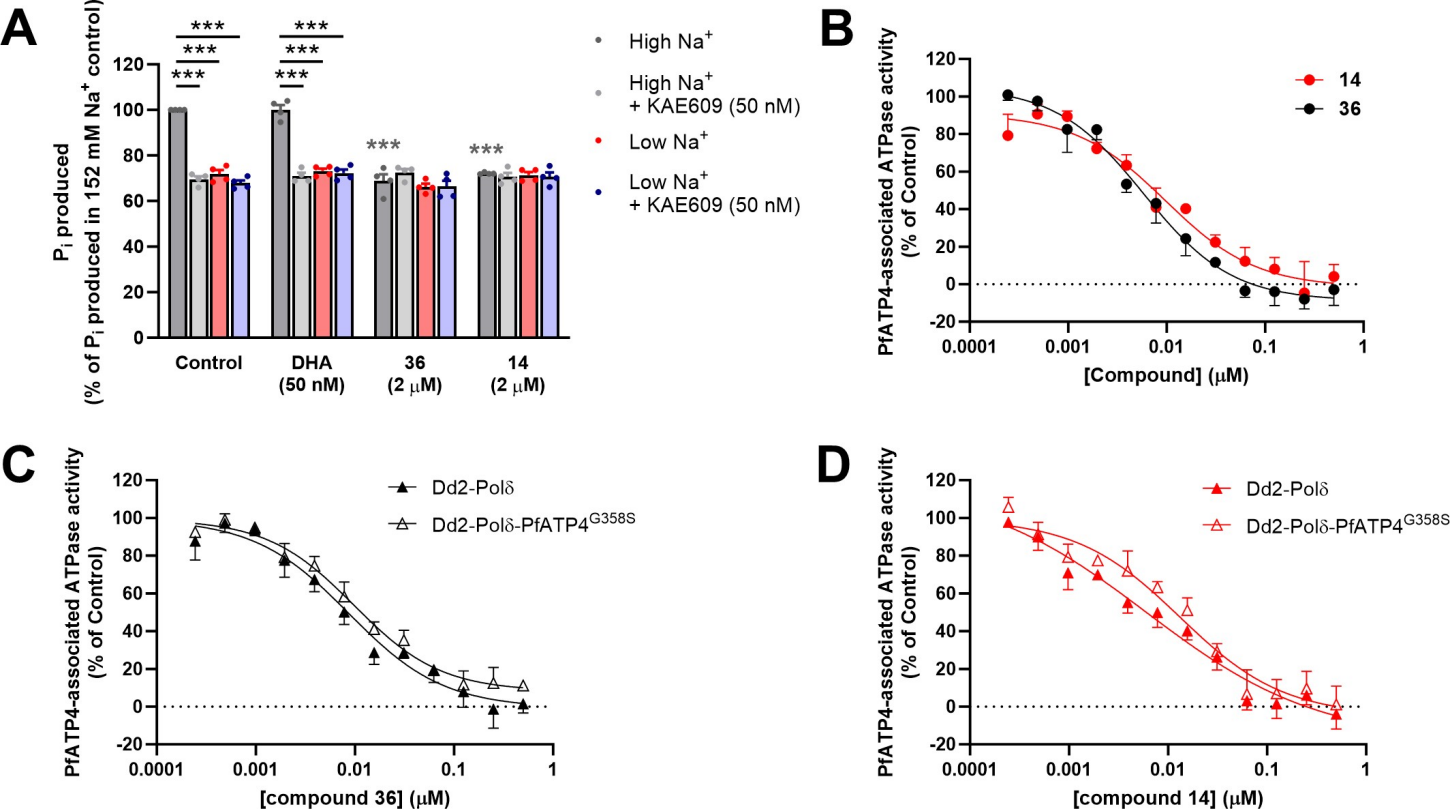
Dihydroquinazolinone analogs **14** and **36** (WJM062) inhibit Na^+^‐ATPase activity in membranes prepared from isolated *P. falciparum* parasites, consistent with them being PfATP4 inhibitors. (A) The effects of dihydroquinazolinone compounds (2 μM), dihydroartemisinin (DHA; 50 nM; negative control) and 0.2 % v/v DMSO (solvent‐only control) on membrane ATPase activity under high‐[Na^+^] conditions (152 mM Na^+^) and low‐[Na^+^] conditions (2 mM Na^+^; stemming from the addition of 1 mM Na_2_ATP) in the presence and absence of the PfATP4 inhibitor cipargamin (50 nM). The membranes were prepared from isolated trophozoite‐stage 3D7 parasites. The Pi produced is expressed as a percentage of that measured in the 152 mM Na^+^ Control. The data are from four independent experiments performed with different membrane preparations; the symbols show the data from individual experiments and the bars show the mean (+SEM). In individual experiments, the Pi produced in the 152 mM Na^+^ Control varied from 66 to 96 nmol per mg (total) protein per min. A repeated measures ANOVA with a post hoc Tukey test was used to compare the (pre‐normalised) data for different compounds and conditions. For comparisons between different conditions for the same test compound (or for the Control), significant differences are shown with black asterisks. For comparisons between the Control and a compound under the same test condition, significant differences are shown with coloured asterisks (in this case, grey asterisks, as the only significant differences were between the control and the dihydroquinazolinone compounds in the 152 mM Na^+^ condition). ***P<0.001. (B) Concentration‐dependence of the effects of dihydroquinazolinone compounds on PfATP4‐associated ATPase activity in membranes prepared from 3D7 parasites. The data are the mean (shown + or − SEM) obtained from four independent experiments performed with different membrane preparations, with the exception of the lowest and second‐lowest concentrations of the compounds, for which data are from two and three independent experiments, respectively. (C and D) Potency of dihydroquinazolinone compounds against PfATP4‐associated ATPase activity in membranes prepared from Dd2‐Polδ‐PfATP4^G358S^ parasites (open triangles) and their Dd2‐Polδ parents (closed triangles). The data are the mean (shown + or − SEM) from four (C) or three (D) independent experiments. In B−D, the Pi production measured in the low‐[Na^+^] (2 mM) condition was subtracted from that measured in high‐[Na^+^] (152 mM) in the presence of each of the different concentrations of dihydroquinazolinone compounds to calculate the PfATP4‐associated ATPase activity. The PfATP4‐associated ATPase activity is expressed as a percentage of that obtained for the high‐[Na^+^] (152 mM) Control.

PfATP4 functions as an ATP‐hydrolysing pump that is believed to extrude Na^+^ ions while importing H^+^ ions, and is essential for maintaining a low Na^+^ concentration (~5–10 mM) in the parasite cytosol.^[21]^ Previous studies have provided evidence that the Na^+^‐dependent ATPase activity measured in membranes prepared from isolated parasites corresponds to PfATP4 activity.[^21^, ^22^, ^25^] We tested representative analogs **14** and **36** (WJM062) for their effects on membrane ATPase activity under high‐[Na^+^] (152 mM) and low‐[Na^+^] (2 mM) conditions and in the presence and absence of the most extensively characterised PfATP4 inhibitor, cipargamin, at a concentration (50 nM) that completely inhibits Na^+^‐ATPase activity. When tested at 2 μM, derivatives **14** and **36** significantly inhibited ATPase activity in the high‐[Na^+^] condition when cipargamin was not present (Figure 4A), reducing ATPase activity to levels similar to those observed under low‐[Na^+^] conditions or when cipargamin was present. These data are consistent with both compounds giving rise to complete inhibition of PfATP4 at 2 μM, while having no effect on Na^+^‐independent ATPases active under the conditions of the experiment. In contrast, the antimalarial dihydroartemisinin (50 nM) had no effect on membrane ATPase activity under any of the conditions tested (Figure 4A). We determined the potency by which analogs **14** and **3** inhibited Na^+^‐ATPase activity in membranes prepared from 3D7 parasites (Figure 4B). The mean EC_50_ values obtained in these experiments were 7.2 and 8.6 nM for analogs **14** and **36**.

We investigated whether the G358S mutation in PfATP4, which confers a high level of resistance to cipargamin and some other PfATP4 inhibitors,^[22]^ affects the potency by which analogs **14** (Figure 4D) and **36** (Figure 4C) inhibit PfATP4‐associated ATPase activity. We prepared membranes from Dd2‐Polδ‐PfATP4^G358S^ parasites (generated previously^[22]^) and Dd2‐Polδ parental parasites^[26]^ to study the activity of Dd2‐PfATP4^WT^ (which has a G1128R mutation relative to 3D7‐PfATP4) and Dd2‐PfATP4^G358S^. The G358S mutation did not have a significant effect on the potency by which compounds inhibit PfATP4. Analog **36** inhibited the Na^+^‐ATPase activities of Dd2‐PfATP4^WT^ and Dd2‐PfATP4^G358S^ with mean EC_50_s of 7.8 and 11.6 nM respectively (*P*=0.15, paired t‐test). For analog **14**, the mean EC_50_s against PfATP4^WT^ and Dd2‐PfATP4^G358S^ were 8.4 and 12.1 nM respectively (*P*=0.35, paired t‐test).

### Activity Against *P. knowlesi* Parasites

PfATP4 inhibitors, including KAE609 and SJ733, show reduced activity against *P. knowlesi* parasites compared to *P. falciparum* parasites.^[27]^ This difference is attributed to the species differences in the ATP4 sequence that may alter inhibitor binding affinity. More recently, ATP4 from different *Plasmodium* species were expressed in *P. knowlesi* parasites confirming the sequence differences in ATP4 between species was responsible for the variation in inhibitor activity.^[28]^ To determine whether the truncated dihydroquinazolinone scaffold also has activity variation between species, we evaluated selected analogues **11**, **14**, **33**, and **36** (WJM062) against *P. knowlesi* YH1 parasites. It was shown that analogues **11**, **14**, **33**, and **36** were between 2‐ and 4‐fold less potent against *P. knowlesi* parasites (EC_50_ 0.033–0.101 μM) compared to *P. falciparum* 3D7 parasites (EC_50_ 0.010–0.045 μM) (Table 7 and Figure S4). SJ733 also showed a 6‐fold decrease in activity against *P. knowlesi* relative to *P. falciparum* 3D7 parasites, consistent with previous findings.^[27]^ These data highlight the differences between the ATP4 inhibitor binding site amino acids between *Plasmodium* species.

**Table 7:** Evaluation of selected analogues against *P. knowlesi* YH1 and multidrug resistant *P. falciparum* strains.

Cmpnd	*P. falciparum* parental EC_50_ (SD) μM^[a]^		*P. knowlesi* EC_50_ (SD) μM^[b]^	multidrug resistant *P. falciparum* strains EC_50_ (SD) μM^[a]^		
	3D7	Dd2	YH1	W2^mef^	7G8	CAM3.1
**11**	0.014 (0.005)	0.017 (0.004)	0.065 (0.042)	0.009 (0.001)	0.033 (0.009)	0.013 (0.001)
**14**	0.016 (0.002)	0.019 (0.001)	0.058 (0.041)	0.017 (0.003)	0.043 (0.006)	0.021 (0.003)
**33**	0.045 (0.003)	0.046 (0.009)	0.101 (0.053)	0.039 (0.08)	0.101 (0.006)	0.060 (0.013)
**36** (WJM062)	0.010 (0.003)	0.013 (0.001)	0.033 (0.021)	0.01 (0.002)	0.031 (0.007)	0.014 (0.003)
SJ733	0.043 (0.027)	‐	0.227 (0.022)	–	–	–

[a] EC_50_ data represent the mean and SD for 3 independent experiments measuring viability of *P. falciparum* parasites over 72 h using SYBR green. [b] EC_50_ data represent the mean and SD for 3 independent experiments measuring *P. knowlesi* YH1 parasite growth over 48 h using SYBR green. See Figures S4 and S5 for dose response curves.

### Activity Against Multidrug Resistant Parasites

To gauge the susceptibility of the truncated dihydroquinazolinone scaffold to common drug resistance mechanisms, we assessed selected analogues against parasite strains that are resistant to antimalarials such as chloroquine, mefloquine and piperaquine. These multidrug‐resistant parasite strains have genetic aberrations in genes that encode CRT and MDR that extrude antimalarials from their principal site of action resulting in decreased parasite activity. It was shown the analogues **11**, **14**, **33**, and **36** (WJM062) have the same activity against the multidrug‐resistant parasite strains compared to the wildtype 3D7 strain (Table 7 and Figure S5), signifying that the dihydroquinazolinone scaffold is not susceptible to mechanisms that confer resistance to common antimalarials.

### Activity Against Transmission Stage Gametes

PfATP4 inhibitors are known to block the transmission of parasites from humans to mosquitoes by interfering with both gametocytes and gamete development.^[29]^ We evaluated a subset of truncated analogues against male and female gametes using a previously described assay. In this assay, stage V gametocytes are treated with compounds and then xanthurenic acid is added and a drop in temperature is used to simulate the mosquito gut environment to induce gametogenesis. After 30 min of compound treatment, male gamete viability is determined by microscopy measuring exflagellation, and after 24 h of compound treatment, female viability is measured using a female gamete specific αPfs25 antibody.

It was shown that compounds **14** and **36** (WJM062) potently inhibited both male and female gamete development (male EC_50_ 0.13 and 0.11 μM) (female EC_50_ 0.12 and 0.21 μM) while the effect by **33** was more modest (male EC_50_ 0.46 μM) (female EC_50_ 1.0 μM) (Table 8 and Figure S6). The gamete activity observed is similar to the activity trend seen between analogues against asexual parasites (Table 4). The transmission phenotype shown by the dihydroquinazolinone analogues **14**, **33** and **36** was consistent with the effects shown by PfATP4 inhibitors, KAE609 and SJ733.[^18^, ^29^]

**Table 8:** *P. falciparum* NF54 gamete activity of analogs.

Cmpnd	Male EC_50_ μM^[a]^	Female EC_50_ μM^[a]^
**14**	0.13	0.12
**33**	0.46	1.0
**36** (WJM062)	0.11	0.21

[a] EC_50_ data represents the average of 43 replicate experiments against *P. falciparum* NF54 stage V gametocytes incubated with test compounds for 48 h prior to inducing gametogenesis. Male gamete formation was quantified 20 min after induction by microscopy measuring exflagellation and female gamete formation measured for 24 h after induction measuring fluorescence using an αPfs25 antibody. See Figure S6 for dose response curves and error.

### Efficacy in a *P. berghei* Mouse Model

Analogues **14** and **33** exhibited potent antimalarial activity, moderate *in vitro* metabolic stability and reasonable aqueous solubility and accordingly were thought to have suitable attributes for evaluation in a 4‐day *P. berghei* mouse model. In this model, mice are infected with *P. berghei* ANKA asexual blood stage parasites. Compounds are then administered by oral gavage at 20 mg/kg 2, 24, 48, and 72 hours after the parasite infection. Blood samples were taken on days 2, 3, and 4 and parasitemia was then determined. Mice treated with analogue **33** showed a moderate decrease in parasite levels (46 %) on day 4 compared to the untreated control group (Table 9 and Figure S7), while analogue **14** showed no decrease in parasitemia. Expectedly the known drug controls, chloroquine and artesunate, reduced parasitemia by >99 % and 96 % on day 4. It is not known whether the compounds **14** and **33** have the same level of activity against *P. berghei* ATP4 as they do for PfATP4, but other studies have shown that ATP4 inhibitors are typically less efficacious in a *P. berghei* than in a *P. falciparum* mouse model.[^18^, ^19^] It is likely that ATP4 species differentiation contributed to the low efficacy of analogues **14** and **33** observed in the *P. berghei* mouse model. Nonetheless, further optimisation of the parasite potency, metabolic stability and aqueous solubility of the truncated dihydroquinazolinone scaffold is required to improve efficacy in mouse models of malaria.

**Table 9:** Evaluation of compounds in a *P. berghei* 4‐day mouse model.

Cmpnd	p.o. dose mg/kg	% parasitemia^[a]^	% reduction in parasitemia^[b]^
**14**	20	100	0.0
**33**	20	12.8	46.7
chloroquine	10	0.02	99.9
artesunate	30	0.66	96

Compounds were administrated q.d. p.o. at the indicated dose at 2, 24, 48 and 72 h after infection with *P. berghei* ANKA parasites expressing GFP. Parasitemia was quantified by measuring bioluminescence. ^a^ Average % parasitemia for 3 mice on day 4. ^b^ Average % reduction in parasitemia versus the vehicle control for 3 mice on day 4. See plotted data and error in Figure S7.

## Conclusions

We deleted the N1‐C1 lactam from the dihydroquinazolinone scaffold and demonstrated that only the C1‐methyl group was required for antimalarial activity. Functionality from the prior studies that produced WJM921 **4** and WJM992 **5** (Figure 1)[^10^, ^16^] was largely transposable onto the truncated scaffold, with retention or a slight loss of antimalarial activity. It was found that aqueous solubility was generally lower with the truncated scaffold unless a polar heterocyclic was included at the N1‐position, but this change was detrimental to metabolic stability. Metabolic stability was enhanced by the inclusion of a polar group such as a heterocyclic or a methoxy group in the 8‐position, although solubility was not significantly improved. The metabolic stability and aqueous solubility and antimalarial activity were directly proportional to lipophilicity and polarity of the truncated scaffold and therefore it remains a challenge to enhance both metabolic stability and solubility while retaining potent antimalarial activity.

It was shown that truncation of the dihydroquinazolinone scaffold did not affect antimalarial phenotype or the molecular target previously shown with dihydroquinazolinone analogues WJM921 **4** and WJM992 **5**.[^10^, ^16^] PfATP4 was confirmed as the molecular target of the truncated analogues by assessing analogues against mutant PfATP4 parasite strains and in an assay that measures PfATP4 activity. In these assays, truncated dihydroquinazolinone analogs were typically shown to have only slightly reduced activity towards the PfATP4^G358S^ parasites compared to PfATP4^WT^ parasites in a growth assay and a PfATP4 Na^+^ assay which is an advantage the dihydroquinazolinone chemotype^10^ has in comparison to KAE609 **1** and SJ733 **2** ‐ which have a much higher loss in potency with this PfATP4 mutation.[^17^, ^18^] Corroborating the transmission stage activity of known PfATP4 inhibitors, the truncated analogues were shown to potently inhibit both male and female gamete formation suggesting that they may have potential in a transmission blocking therapy. Truncated analogues had decreased activity against *P. knowlesi* parasites, which is a common finding with PfATP4 inhibitors[^27^, ^28^] due to the sequence diversity of ATP4 between *Plasmodium* species. In addition to the moderate metabolic stability and aqueous solubility, the species differentiation may have contributed to the modest efficacy of truncated analogues observed in the *P. berghei* mouse model. The balance of physicochemical properties and antimalarial activity remains a challenge in the future optimisation of the dihydroquinazolinone scaffold. Optimising these attributes may see this scaffold join the arsenal of new chemotypes in preclinical development for the treatment of malaria.

## Experimental Section

### Chemistry Experimental

#### General Chemistry Methods

Solvents and reagents were obtained commercially and used without further purification. NMR spectra were recorded on either a Bruker Ascend 300 or a Bruker Ultrashield 400. Spectra were processed using MestReNova 14.3 software. Chemical shifts (δ) are recorded in parts per million (ppm) and referenced to the corresponding solvent signal. Coupling constants (*J*) are recorded in Hertz (Hz) and multiplicities are described by singlet (s), broad singlet (br s) doublet (d), triplet (t), quartet (q), doublet of doublets (dd), doublet of triplets (dt), doublet of doublet of doublets (ddd) and multiplet (m). Column chromatography (FCC) was conducted with silica gel using pre‐packed Phenomenex (particle size 40–60 μm) in combination with a CombiFlash NextGen or with pre‐packed SepaFlash columns (particle size 40–63 μm) in combination with a CombiFlash Rf 200, CombiFlash NextGen or Biotage Selekt. Reverse phase preparative HPLC was carried out using a YMC‐Actus Triart C18 column with a MeCN and aqueous 10 mM NH_4_OAc mobile phase. LCMS was recorded on an Agilent LCMS system composed of an AgilentG6120B Mass Detector, 1260 Infinity G1312B Binary pump, 1260 Infinity G1367E HiPALS autosampler, and 1260 Infinity G4212B Diode Array Detector. Final compounds were determined to be >95 % pure using this method, unless stated otherwise. HRMS was acquired through the Bio21 Mass Spectrometry and Proteomics Facility using a Thermo Scientific nano‐LC Q Exactive plus mass spectrometer.

#### Synthesis Procedures

General Procedure A: *Formation of dihydroquinazolinone scaffold via cyclo‐condensation with pyruvic acid*. To a stirred solution of the appropriate anthranilamide (1 eq) in glacial AcOH (5 mL) was added pyruvic acid (5 eq) and the mixture was heated at reflux for 2 h. The crude mixture was then concentrated *in vacuo*. Et_2_O (8 mL) was then added to the resultant residue and the mixture was stirred for 30 min. The formed precipitate was then collected by decantation and the residue was then washed with additional Et_2_O and dried under vacuum to afford corresponding dihydroquinazolinone.

General Procedure B: *HATU mediated amide coupling*. To a cooled solution of the appropriate carboxylic acid (1 eq) in DMF (5 mL) under N_2_ was added HATU (1.5 eq) and DIPEA (2 eq). The mixture was stirred for 10 min before the addition of the appropriate amine (1.3 eq), and then stirred at rt for an additional 3 h. Upon completion, the reaction mixture is diluted with EtOAc (20 mL) and then washed with cold water (2 x 20 mL), followed by brine (1 x 30 mL). The organic layer was then dried over anhyd. MgSO_4_, filtered and concentrated *in vacuo*. The crude product was then purified via FCC to afford the corresponding amide.

General Procedure C: *Formation of dihydroquinazolinone scaffold via ring opening of tetrahydropyrroloquinazolines*. To a stirred solution of the appropriate tetrahydropyrroloquinazoline (1 eq) in anhyd. THF was added to the respective secondary amine (10 eq) and the mixture was cooled to 0 °C. A solution of 2 M AlMe_3_ in toluene (10 eq) was then added and the reaction mixture was stirred at ambient temperature for 16 h. Upon completion, the reaction was quenched with 20 % aq. NaOH, filtered, and concentrated *in vacuo*. The crude product was then purified via FCC to afford the desired product.

*7‐Chloro‐N‐(3‐chlorophenyl)‐2,3‐dimethyl‐4‐oxo‐1,2,3,4‐tetrahydroquinazoline‐2‐carboxamide* (**11**). Compound **37** (200 mg, 0.785 mmol) was reacted with 3‐chloroaniline (109 μL, 1.18 mmol) under conditions in General Procedure B to afford compound **11** as a white solid (240 mg, 84 %).^1^H NMR (400 MHz, DMSO‐d_6_): δ 10.12 (s, 1H), 7.78 (s, 1H), 7.65 (d, *J*=8.4 Hz, 1H), 7.54 (d, *J*=8.0 Hz, 1H), 7.46 (s, 1H), 7.34 (t, *J*=8.0 Hz, 1H), 7.15 (d, *J*=8.0 Hz, 1H), 6.79–6.75 (m, 2H), 2.94 (s, 3H), 1.72 (s, 3H) ppm. ^13^C NMR (75 MHz, DMSO‐d_6_) δ 170.0, 161.9, 146.2, 139.8, 137.6, 133.0, 130.4, 129.6, 123.6, 119.4, 118.3, 118.1, 113.6, 113.5, 75.7, 28.9, 22.2 ppm. HRMS m/z: [M+H]^+^ calcd for C_17_H_15_Cl_2_N_3_O_2_ 364.0614; Found 364.0607.

*7‐Chloro‐1,2,3‐trimethyl‐4‐oxo‐1,2,3,4‐tetrahydroquinazoline‐2‐carboxylic acid* (**38**). 2‐Amino‐4‐chloro‐*N*‐methylbenzamide (800 mg, 4.03 mmol) was reacted according to General procedure A to afford compound **38** as a brown powder (603 mg, 56 %). ^1^H NMR (400 MHz, DMSO‐d_6_): *δ* 13.45 (br s, 1H), 7.71 (d, *J*=8.0 Hz, 1H), 6.92 (d, *J*=1.6 Hz, 1H), 6.86 (dd, *J*=8.0 Hz, 1.6 Hz, 1H), 3.00 (s, 3H), 2.95 (s, 3H), 1.78 (s, 3H) ppm. LCMS (ESI) m/z: 269.2 [M+H]^+^.

*7‐Chloro‐N‐(3‐chlorophenyl)‐1,2,3‐trimethyl‐4‐oxo‐1,2,3,4‐tetrahydroquinazoline‐2‐carboxamide* (**12**). Compound 38 (180 mg, 0.671 mmol) was reacted with 3‐chloroaniline (107 μL, 1.01 mmol) according to General procedure B to afford 12 (110 mg, 43 %). ^1^H NMR (400 MHz, DMSO‐d_6_): *δ* 10.74 (s, 1H), 7.87–7.84 (m, 1H), 7.78 (d, *J*=8.4 Hz, 1H), 7.68–7.63 (m, 1H), 7.38 (t, *J*=8.0 Hz, 1H), 7.18 (dd, *J*=8.0 Hz, 1.6 Hz, 1H), 6.93–6.88 (m, 2H), 2.82 (s, 3H), 2.72 (s, 3H), 1.46 (s, 3H) ppm. ^13^C NMR (75 MHz, DMSO‐d_6_) δ 168.2, 160.5, 147.4, 139.8, 138.6, 133.0, 130.4, 129.3, 123.8, 119.7, 118.6, 118.1, 114.0, 112.5, 82.3, 33.4, 29.3, 15.4 ppm. HRMS m/z: [M+H]^+^ calcd for C_18_H_17_Cl_2_N_3_O_2_ 378.0771; Found 378.0764.

*7‐Chloro‐N‐(3‐chlorophenyl)‐1‐ethyl‐2,3‐dimethyl‐4‐oxo‐1,2,3,4‐tetrahydroquinazoline‐2‐carboxamide* (**13**). 7‐Chloro‐1‐ethyl‐2,3‐dimethyl‐4‐oxo‐1,2,3,4‐tetrahydroquinazoline‐2‐carboxylic acid (190 mg, 0.672 mmol) was reacted with 3‐chloroaniline (107 μL, 1.01 mmol) according to General procedure B to afford compound 13 as a white solid (50 mg, 19 %). ^1^H NMR (400 MHz, DMSO‐d_6_) δ 10.75 (s, 1H), 7.85 (s, 1H), 7.78 (d, *J*=8.0 Hz, 1H), 7.64 (d, *J*=8.0 Hz, 1H), 7.38 (t, *J*=8.0 Hz, 1H), 7.18 (dd, *J*=8.0 Hz, 1.6 Hz, 1H), 6.92–6.86 (m, 2H), 3.37–3.30 (m, 1H), 3.10–3.01 (m, 1H), 2.82 (s, 3H), 1.51 (s, 3H), 1.06 (t, *J*=6.8 Hz, 3H) ppm. ^13^C NMR (75 MHz, DMSO‐d_6_) δ 168.7, 161.3, 146.7, 140.2, 139.0, 133.6, 131.0, 130.1, 124.4, 120.1, 119.0, 118.6, 115.3, 113.4, 82.5, 41.8, 29.8, 17.5, 13.0 ppm. HRMS m/z: [M+H]^+^ calcd for C_19_H_19_Cl_2_N_3_O_2_ 392.0927; Found 392.0918.

*7‐Chloro‐N‐(5‐chloro‐3‐pyridyl)‐2,3‐dimethyl‐4‐oxo‐1H‐quinazoline‐2‐carboxamide* (**14**). Compound **37** (121 mg, 0.475 mmol) was reacted with 5‐chloropyridin‐3‐amine (99 mg, 0.770 mmol) according to General procedure B to afford **14** as a tan powder (75 mg, 43 %). ^1^H NMR (300 MHz, DMSO‐d_6_) δ 10.43 (s, 1H), 8.75 (d, *J*=2.2 Hz, 1H), 8.36 (d, *J*=2.2 Hz, 1H), 8.22 (t, *J*=2.2 Hz, 1H), 7.66 (d, *J*=8.2 Hz, 1H), 7.50 (s, 1H), 6.95–6.62 (m, 2H), 2.93 (s, 3H), 1.72 (s, 3H) ppm. ^13^C NMR (75 MHz, DMSO‐d_6_) δ 170.4, 161.8, 146.1, 139.7, 137.7, 136.0, 130.5, 129.6, 126.2, 118.2, 113.7, 113.3, 75.8, 28.9, 22.0 ppm. HRMS m/z: [M+H]^+^ calcd for C_16_H_14_Cl_2_N_4_O_2_ 365.0567; Found 365.0561.

*N‐(5‐Chloropyridin‐3‐yl)‐2‐oxopropanamide* (**39**). To a stirred solution of pyruvic acid (1.37 g, 15.6 mmol) in DMF (12 mL) was added SOCl_2_ (1.13 mL, 15.6 mmol) dropwise at 0 °C under N_2_. The reaction mixture was stirred for 10 min at 0 °C and then 5‐chloropyridin‐3‐amine (1.0 g, 7.78 mmol) was added. The reaction was then stirred at ambient temperature for 16 h. The reaction mixture was then quenched with sat. NaHCO_3_ and extracted with EtOAc. The combined organic layers were washed with cold water and brine, dried over Na_2_SO_4_, filtered, and concentrated *in vacuo*. The crude product was purified via FCC to afford compound **39** as a white semi‐solid (305 mg, 20 %). ^1^H NMR (400 MHz, DMSO‐d_6_) δ 10.88 (s, 1H), 8.95 (d, *J*=2.0 Hz, 1H), 8.39 (d, *J*=2.0 Hz, 1H), 8.36 (d, *J*=2.0 Hz, 1H), 2.44 (s, 3H) ppm. LCMS (ESI) m/z: 197.0 [M−H]^−^.

*7‐Chloro‐N‐(5‐chloropyridin‐3‐yl)‐1,2,3‐trimethyl‐4‐oxo‐1,2,3,4‐tetrahydroquinazoline‐2‐carboxamide* (**15**). Compound **39** (168 mg, 0.846 mmol) was reacted with 4‐chloro‐*N*‐methyl‐2‐(methylamino)benzamide (112 mg, 0.564 mmol) according to General procedure A. The crude material was then purified via reverse phase preparative HPLC to afford compound **15** as a white solid (43 mg, 20 %). ^1^H NMR (400 MHz, DMSO‐d_6_) δ 10.98 (s, 1H), 8.83 (s, 1H), 8.39 (d, *J*=1.6 Hz, 1H), 8.29 (s, 1H), 7.79 (d, J=8.8 Hz, 1H), 6.94–6.91 (m, 2H), 2.83 (s, 3H), 2.73 (s, 3H), 1.48 (s, 3H) ppm. ^13^C NMR (75 MHz, DMSO‐d_6_) δ 168.8, 160.5, 147.3, 143.2, 139.9, 138.7, 136.0, 130.5, 129.3, 126.6, 118.2, 113.9, 112.6, 82.3, 33.5, 29.4, 15.3 ppm. HRMS m/z: [M+H]^+^ calcd for C_17_H_16_Cl_2_N_4_O_2_ 379.0723; Found 379.0713.

*7‐Chloro‐N‐(5‐chloropyridin‐3‐yl)‐3‐cyclopropyl‐2‐methyl‐4‐oxo‐1,2,3,4‐tetrahydroquinazoline‐2‐carboxamide* (**16**). 2‐Amino‐4‐chloro‐*N*‐cyclopropylbenzamide (272 mg, 1.29 mmol) was reacted with compound **39** (308 mg, 1.55 mmol) according to General procedure A. The crude material was then purified via reverse phase preparative HPLC to afford compound **16** (25 mg, 5 %). ^1^H NMR (400 MHz, DMSO‐d_6_) δ 10.28 (s, 1H), 8.69 (s, 1H), 8.35 (s, 1H), 8.18 (s, 1H), 7.65 (d, *J*=8.6 Hz, 1H), 7.49 (s, 1H), 6.82–6.75 (m, 2H), 1.86 (s, 3H), 0.89–0.71 (m, 3H), 0.65–0.56 (m, 1H) ppm. ^13^C NMR (75 MHz, DMSO‐d_6_) δ 171.6, 163.9, 146.1, 143.0, 139.6, 137.6, 135.9, 130.5, 129.8, 126.1, 118.5, 114.4, 114.0, 76.7, 25.8, 22.5, 8.8, 7.4 ppm. HRMS m/z: [M+H]^+^ calcd for C_18_H_16_Cl_2_N_4_O_2_ 391.0724; Found 391.0729.

*7‐Chloro‐N‐(5‐chloropyridin‐3‐yl)‐3‐cyclopropyl‐1,2‐dimethyl‐4‐oxo‐1,2,3,4‐tetrahydroquinazoline‐2‐carboxamide* (**17**). 4‐Chloro‐*N*‐cyclopropyl‐2‐(methylamino)benzamide (215 mg, 0.957 mmol) was reacted with compound **39** (285 mg, 1.44 mmol) according to General procedure A. The crude material was then purified via reverse phase preparative HPLC to afford compound **17** as a white solid (31 mg, 8 %). ^1^H NMR (400 MHz, DMSO‐d_6_) δ 10.73 (s, 1H), 8.79 (s, 1H), 8.37 (s, 1H), 8.24 (s, 1H), 7.77 (d, *J*=8.0 Hz, 1H), 6.99 (s, 1H), 6.93 (d, *J*=8.0 Hz, 1H), 2.79 (s, 3H), 1.61 (s, 3H), 0.86–0.71 (m, 3H), 0.61–0.57 (m, 1H) ppm. ^13^C NMR (75 MHz, DMSO‐d_6_) δ 169.1, 162.8, 147.9, 143.0, 139.8, 138.5, 136.0, 130.5, 129.5, 126.4, 119.0, 115.7, 114.2, 82.2, 33.9, 26.9, 17.9, 7.6 ppm. HRMS m/z: [M+H]^+^ calcd for C_19_H_18_Cl_2_N_4_O_2_ 405.0880; Found 405.0873.

*8‐Chloro‐4‐methyl‐1,5‐dioxo‐2,3,4,5‐tetrahydropyrrolo[1,2‐a]quinazoline‐3a(1H)‐carboxylic acid* (**40**). 2‐Amino‐4‐chloro‐*N*‐methylbenzamide (2.5 g, 13.6 mmol) was reacted according to General procedure A, to afford compound **40** (2.6 g, 65 %). ^1^H NMR (400 MHz, DMSO‐d_6_) δ 14.15 (br s, 1H), 8.28 (d, *J*=1.6 Hz, 1H), 7.92 (d, *J*=8.4 Hz, 1H), 3.36 (dd, *J*=8.4 Hz, 1.6 Hz, 1H), 3.06 (s, 3H), 2.81–2.52 (m, 4H) ppm. LCMS (ESI) m/z: 293.39 [M+H]^+^.

7‐Chloro‐2,3‐dimethyl‐4‐oxo‐1,2,3,4‐tetrahydroquinazoline‐2‐carboxylic acid (**37**). To a stirred solution of 2‐amino‐4‐chloro‐N‐methylbenzamide (0.400 g, 2.17 mmol) in glacial acetic acid (5 ml) was added pyruvic acid (0.763 ml, 10.8 mmol) and the mixture was heated to reflux for 2 h. On completion, the reaction mixture was cooled to room temperature and concentrated under reduced pressure. Diethyl ether (8 ml) was added to the crude mixture and stirred for 30 min. The solid precipitate thus formed was collected by decantation. The residue was washed with diethyl ether and dried under vacuum to afford **37** (0.300 g, 54 %) as off‐white powder. ^1^H NMR (400 MHz, DMSO‐d_6_): δ 13.24 (brs, 1H), 7.66 (s, 1H), 7.59 (d, *J*=8.0 Hz, 1H), 6.77 (d, *J*=1.6 Hz, 1H), 6.73 (dd, *J*=8.0 Hz, 1.6 Hz, 1H), 2.97 (s, 3H), 1.72 (s, 3H) ppm. LCMS (ESI) m/z: 255.28 [M+H]^+^.

*8‐Chloro‐N‐(5‐chloropyridin‐3‐yl)‐4‐methyl‐1,5‐dioxo‐2,3,4,5‐tetrahydropyrrolo[1,2‐a]quinazoline‐3a(1H)‐carboxamide* (**41**). Compound **40** (500 mg, 1.7 mmol) was reacted with 5‐chloropyridin‐3‐amine (262 mg, 2.04 mmol) according to General procedure B to afford compound **41** as an off‐white solid (130 mg, 19 %). ^1^H NMR (400 MHz, DMSO‐d_6_) δ 10.21 (s, 1H), 8.63 (d, *J*=1.6 Hz, 1H), 8.35 (d, *J*=2.0 Hz, 1H), 8.07 (s, 1H), 7.96 (d, *J*=1.6 Hz, 1H), 7.91 (t, *J*=8.0 Hz, 1H), 7.40 (dd, *J*=8.4 Hz, 1.6 Hz, 1H), 3.31 (s, 3H), 3.21 (s, 3H), 2.95–2.85 (m, 2H), 2.81–2.67 (m, 2H) ppm. LCMS (ESI) m/z: 405.03 [M+H]^+^.

*8‐Chloro‐N‐(3‐chlorophenyl)‐4‐methyl‐5‐oxo‐2,3,4,5‐tetrahydropyrrolo[1,2‐a]quinazoline‐3a(1H)‐carboxamide* (**18**). To a stirred solution of 2‐amino‐4‐chloro‐N‐methylbenzamide (0.500 g, 2.71 mmol) in AcOH (5 mL) was added methyl 5‐bromo‐2‐oxopentanoate (0.679 g, 3.25 mmol) and the mixture was refluxed for 4 h. The reaction mixture was cooled to room temperature and concentrated under reduced pressure. The residue was basified with 1 N NaOH solution, washed with ethyl acetate (2 x). The organic phase was discarded, and the aqueous phase was acidified with 1 N HCl and extracted with ethyl acetate (3 x). The combined organic phase was washed with brine, dried over anhydrous sodium sulfate to afford the intermediate 8‐chloro‐4‐methyl‐5‐oxo‐2,3,4,5‐tetrahydropyrrolo[1,2‐a]quinazoline‐3a(1H)‐carboxylic acid (0.330 g, 43 %) as off‐white solid. ^1^H NMR (400 MHz, DMSO‐d_6_): δ 13.39 (brs, 1H), 7.65 (d, *J*=8.0 Hz, 1H), 6.83–6.71 (m, 2H), 3.62–3.58 (m, 1H), 3.54–3.47 (m, 1H), 2.97 (s, 3H), 2.56–2.23 (m, 4H, overlapped with DMSO solvent peak), 2.20–2.02 (m, 2H); LCMS (ESI) m/z: 281.17 [M+H]^+^. To a stirred solution of 8‐chloro‐4‐methyl‐5‐oxo‐2,3,4,5‐tetrahydropyrrolo[1,2‐a]quinazoline‐3a(1H)‐carboxylic acid (0.300 g, 1.07 mmol in THF (7.0 mL) was added HATU (0.813 g, 2.14 mmol) and DIPEA (0.93 mL, 5.34 mmol) at room temperature. After 5 min, 3‐chloroaniline (0.169 mL, 1.02 mmol) was added and the mixture was heated at 60 °C overnight. The mixture was diluted with EtOAc and washed with water and brine. The organic layer was dried over anhydrous sodium sulfate and concentrated under reduced pressure. The crude residue was purified initially by flash column chromatography (25–40 % EtOAc in hexane) followed by reverse phase preparative HPLC [Column: YMC Actus Triart C18, Mobile phase: AcCN and 20 mM NH_4_HCO_3_ aqueous buffer] to afford compound **18** (0.010 g, 2 %) as a white solid. ^1^H NMR (400 MHz, DMSO‐d_6_): δ 9.46 (s, 1H), 7.67 (d, *J*=8.0 Hz, 1H), 7.63 (s, 1H), 7.47 (d, *J*=8.0 Hz, 1H), 7.31 (t, *J*=8.0 Hz, 1H), 7.15 (d, *J*=8.0 Hz, 1H), 6.97 (d, *J*=1.2 Hz, 1H), 6.85 (d, *J*=8.0 Hz, 1H), 4.10–4.05 (m, 1H), 3.45 (q, *J*=8.0 Hz, 1H), 3.16 (s, 3H), 2.68–2.54 (m, 2H), 2.15–2.01 (m, 2H); HPLC purity: 99.52 %; LCMS (Formic acid : ACN): m/z: 390.23 [M+H]^+^, t_R_=3.09 min.

*7‐Chloro‐N‐(3‐chlorophenyl)‐3‐methyl‐4‐oxo‐1,2,3,4‐tetrahydroquinazoline‐2‐carboxamide* (**19**). To a stirred solution of 2‐amino‐4‐chloro‐N‐methylbenzamide (0.400 g, 2.167 mmol) in glacial acetic acid (5 mL) was added glyoxylic acid (0.239 g, 2.6 mmol) and the mixture was heated to reflux for 4 h. On completion, the reaction mixture was cooled to room temperature and concentrated under reduced pressure and co‐evaporated with toluene (2 x) to get crude 7‐chloro‐3‐methyl‐4‐oxo‐1,2,3,4‐tetrahydroquinazoline‐2‐carboxylic acid. To a solution of the crude 7‐chloro‐3‐methyl‐4‐oxo‐1,2,3,4‐tetrahydroquinazoline‐2‐carboxylic acid (0.375 g, 42 % pure by LCMS, effectively 0.158 g, 0.66 mmol) in dry THF (8 mL) were added DIPEA (0.570 ml, 3.27 mmol), HATU (0.374 g, 0.983 mmol) and 3‐chloroaniline (0.139 ml, 1.31 mmol) and the reaction mixture was heated at 60 °C while stirring for 16 h. The reaction mixture was then cooled, water was added and extracted with ethyl acetate (3 x). The combined organic phase was dried over anhydrous sodium sulfate, filtered and concentrated under reduced pressure. Crude residue was purified by flash column chromatography (40–60 % ethyl acetate in hexane) followed by reverse phase Preparative HPLC [Column: YMC Actus Triart C18, Mobile phase: AcCN and 20 mM NH_4_HCO_3_ aqueous buffer] to afford compound **19** (0.070 g, 30 %). ^1^H NMR (400 MHz, DMSO‐d_6_): δ 10.42 (s, 1H), 7.78 (s, 1H), 7.60 (d, *J*=8.0 Hz, 1H), 7.45–7.38 (m, 2H), 7.36 (t, *J*=8.0 Hz, 1H), 7.15 (d, *J*=8.0 Hz, 1H), 6.75 (d, *J*=1.2 Hz, 1H), 6.71 (d, *J*=8.4 Hz, 1H), 5.28 (d, *J*=2.4 Hz, 1H), 2.91 (s, 3H) ppm; LCMS: m/z: 350.23 [M+H]+, t_R_=2.81 min. HRMS m/z: [M+H^+^] calcd for C_16_H_13_Cl_2_N_3_O_2_ 350.0459; Found 350.0460.

7‐Chloro‐N‐(5‐chloropyridin‐3‐yl)‐2‐(3‐(dimethylamino)‐3‐oxopropyl)‐3‐methyl‐4‐oxo‐1,2,3,4‐tetrahydroquinazoline‐2‐carboxamide (**20**). Compound **41** (100 mg, 0.247 mmol) was reacted with N,N‐dimethylamine (2 M in THF, 1.23 mL, 2.47 mmol) according to General procedure C to afford compound **20** as a white solid (25 mg, 22 %). ^1^H NMR (400 MHz, DMSO‐d_6_) δ 10.48 (s, 1H), 8.75 (s, 1H), 8.37 (s, 1H), 8.23 (s, 1H), 7.64 (d, J=8.8 Hz, 1H), 7.50 (s, 1H), 6.77–6.71 (m, 2H), 2.92 (s, 3H), 2.88 (s, 3H), 2.80 (s, 3H), 2.56–2.50 (m, 2H) ppm. ^13^C NMR (75 MHz, DMSO‐d_6_) δ 170.8, 170.1, 161.9, 146.2, 143.1, 139.8, 137.8, 135.9, 130.5, 129.4, 126.5, 117.7, 113.2, 112.5, 78.2, 36.6, 35.0, 30.1, 29.3, 26.8 ppm. HRMS m/z: [M+H]^+^ calcd for C_20_H_21_Cl_2_N_5_O_3_ 450.1094; Found 450.1084.

*7‐Chloro‐N‐(5‐chloropyridin‐3‐yl)‐3‐methyl‐2‐(3‐morpholino‐3‐oxopropyl)‐4‐oxo‐1,2,3,4‐tetrahydroquinazoline‐2‐carboxamide* (**21**). Compound **41** (200 mg, 0.494 mmol) was reacted with morpholine (129 mg, 1.48 mmol) according to General procedure C to afford compound **21** as a white solid (30 mg, 12 %). ^1^H NMR (400 MHz, DMSO‐d_6_) δ 10.47 (s, 1H), 8.75 (s, 1H), 8.37 (s, 1H), 8.23 (s, 1H), 7.65 (d, *J*=8.4 Hz, 1H), 7.48 (s, 1H), 6.78–6.67 (m, 2H), 3.59–3.43 (m, 4H), 3.42–3.30 (m, 4H), 2.92 (s, 3H), 2.55–2.50 (m, 1H), 2.49–2.30 (m, 3H) ppm. ^13^C NMR (75 MHz, DMSO‐d_6_) δ 170.0, 169.8, 161.9, 146.2, 143.1, 139.9, 137.8, 135.9, 130.5, 129.5, 126.5, 117.7, 113.2, 112.5, 78.2, 66.0, 45.3, 41.6, 30.0, 29.3, 26.5 ppm. HRMS m/z: [M+H]^+^ calcd for C_22_H_23_Cl_2_N_5_O_4_ 492.1200; Found 492.1189.

*tert‐Butyl 4‐(3‐(7‐chloro‐2‐((5‐chloropyridin‐3‐yl)carbamoyl)‐3‐methyl‐4‐oxo‐1,2,3,4‐tetrahydroquinazolin‐2‐yl)propanoyl)piperazine‐1‐carboxylate* (**42**). Compound **41** (400 mg, 0.987 mmol) was reacted with tert‐butyl piperazine‐1‐carboxylate (1.84 g, 9.87 mmol) according to General procedure C to afford compound **42** as an off‐white solid (120 mg, 21 %). ^1^H NMR (400 MHz, DMSO‐d_6_): δ 10.47 (s, 1H), 8.75 (s, 1H), 8.36 (d, *J*=1.6 Hz, 1H), 8.23 (s, 1H), 7.64 (d, *J*=8.8 Hz, 1H), 7.49 (s, 1H), 6.77–6.71 (m, 2H), 3.42–3.40 (m, 2H), 3.39–3.30 (m, 2H), 3.30–3.21 (m, 4H), 2.90 (s, 3H), 2.61–2.51 (m, 1H) ppm. LCMS: m/z: 591.60 [M+H]^+^.

*7‐Chloro‐N‐(5‐chloropyridin‐3‐yl)‐3‐methyl‐4‐oxo‐2‐(3‐oxo‐3‐(piperazin‐1‐yl)propyl)‐1,2,3,4‐tetrahydroquinazoline‐2‐carboxamide* (**22**). To a stirred solution of **42** (50 mg, 0.085 mmol) in EtOH (3 mL) at 0 °C was added an excess of 2 N HCl in Et_2_O, which was reacted for 3 h. Upon completion, volatiles were removed *in vacuo* and the resulting residue was triturated with EtOH:Et_2_O (3 : 7) to afford the mono‐hydrochloride salt of compound **22** as a green solid (40 mg, 90 %). ^1^H NMR (400 MHz, DMSO‐d_6_) δ 11.03 (s, 1H), 8.92 (br s, 2H), 8.42 (br s, 1H), 8.25 (s, 1H), 7.63 (d, *J*=8.4 Hz, 1H), 6.87 (s, 1H), 6.76 (dd, *J*=8.4 Hz, 1.6 Hz, 1H), 3.72–3.60 (m, 4H), 3.18–3.00 (m, 4H), 2.98 (s, 3H), 2.77–2.66 (m, 1H), 2.48–2.30 (m, 2H) ppm. HRMS m/z: [M+H]^+^ calcd for C_22_H_24_Cl_2_N_6_O_3_ 491.1361; Found 491.1362.

*7‐Chloro‐N‐(5‐chloropyridin‐3‐yl)‐3‐methyl‐2‐(3‐(4‐methylpiperazin‐1‐yl)‐3‐oxopropyl)‐4‐oxo‐1,2,3,4‐tetrahydroquinazoline‐2‐carboxamide* (**23**). Compound **41** (100 mg, 0.247 mmol) was reacted with 1‐methylpiperazine (247 mg, 2.47 mmol) according to General procedure C to afford compound **23** as a white solid (64 mg, 51 %). ^1^H NMR (400 MHz, DMSO‐d_6_) δ 10.47 (s, 1H), 8.75 (s, 1H), 8.36 (s, 1H), 8.23 (s, 1H), 7.64 (d, *J*=8.8 Hz, 1H), 7.47 (br s, 1H), 6.76–6.68 (m, 2H), 3.48–3.30 (m, 4H), 2.92 (s, 3H), 2.59–2.52 (m, 1H), 2.49–2.25 (m, 3H), 2.24–2.16 (m, 4H), 2.15 (s, 3H) ppm. ^13^C NMR (75 MHz, DMSO‐d_6_) δ 170.0, 169.4, 161.9, 146.2, 143.1, 139.9, 137.8, 135.9, 130.5, 129.5, 126.5, 117.7, 113.2, 112.5, 78.2, 54.6, 54.2, 45.6, 44.7, 41.1, 30.0, 29.3, 26.6 ppm. HRMS m/z: [M+H]^+^ calcd for C_23_H_26_Cl_2_N_6_O_3_ 505.1516; Found 505.1507.

*Benzyl 4‐(3‐(7‐chloro‐2‐((5‐chloropyridin‐3‐yl)carbamoyl)‐3‐methyl‐4‐oxo‐1,2,3,4‐tetrahydroquinazolin‐2‐yl)propanoyl)piperazine‐1‐carboxylate* (**24**). Compound **41** (410 mg, 1.01 mmol) was reacted with benzyl piperazine‐1‐carboxylate (1.96 mL, 10.1 mmol) according to General procedure C to afford compound **24** as a white solid (80 mg, 13 %). ^1^H NMR (400 MHz, DMSO‐d_6_) δ 10.47 (s, 1H), 8.75 (s, 1H), 8.36 (d, *J*=1.6 Hz, 1H), 8.23 (s, 1H), 7.64 (d, *J*=8.8 Hz, 1H), 7.49 (s, 1H), 7.39–7.29 (m, 5H), 6.76–6.71 (m, 2H), 5.09 (s, 2H), 3.49–3.30 (m, 8H), 2.92 (s, 3H), 2.60–2.28 (m, 4H) ppm. ^13^C NMR (75 MHz, DMSO‐d_6_) δ 170.0, 169.8, 161.9, 154.4, 146.2, 143.1, 139.9, 137.8, 136.8, 135.9, 130.5, 129.5, 128.4, 127.9, 127.6, 126.5, 117.7, 113.2, 112.5, 78.2, 66.3, 44.4, 43.4, 40.9, 30.0, 29.3, 26.7 ppm. HRMS m/z: [M+H]^+^ calcd for C_30_H_30_Cl_2_N_6_O_5_ 625.1729; Found 625.1736.

*N‐(5‐Chloropyridin‐3‐yl)‐2,3‐dimethyl‐4‐oxo‐1,2,3,4‐tetrahydropyrido[3,2‐h]quinazoline‐2‐carboxamide* (**25**). To a solution of 8‐aminoquinoline‐7‐carboxylic acid (0.200 g, 1.03 mmol) in DMF (8.0 mL) at 0 °C were successively added EDC.HCl (0.306 g, 1.59 mmol), HOBt (0.172 g, 1.28 mmol) and DIPEA (0.93 mL, 5.31 mmol). The mixture was stirred for 5 min under nitrogen, and thereafter methylamine hydrochloride (0.144 g, 2.13 mmol) was added and stirring was continued at room temperature overnight. The mixture was diluted with ethyl acetate, washed with cold water (3 x) and brine. The organic phase was then dried over anhydrous sodium sulfate, filtered and concentrated under reduced pressure. The residue was triturated with diethyl ether and pentane (3 : 7) to obtain the intermediate 8‐amino‐N‐methylquinoline‐7‐carboxamide (0.160 g, 75 %) as white solid. ^1^H NMR (400 MHz, DMSO‐d_6_): δ 8.78 (dd, *J*=4.0 Hz, 1.6 Hz, 1H), 8.29 (d, *J*=4.4 Hz, 1H), 8.20 (dd, *J*=8.0 Hz, 1.6 Hz, 1H), 6.68 (d, *J*=8.8 Hz, 1H), 7.58–7.55 (m, 3H), 7.0 (d, *J*=8.4 Hz, 1H), 2.79 (d, *J*=4.4 Hz, 3H); LCMS (ESI) m/z: 202.06 [M+H]^+^. To a stirred solution of 8‐amino‐N‐methylquinoline‐7‐carboxamide (0.080 g, 0.398 mmol) in AcOH (3.0 mL) was added N‐(5‐chloropyridin‐3‐yl)‐2‐oxopropanamide (0.095 g, 0.477 mmol) and the mixture was refluxed for 6 h. On completion, the reaction mixture was cooled to room temperature and concentrated under reduced pressure and co‐evaporated with toluene (2 x). The crude residue was purified by flash column chromatography (60–90 % EtOAc in hexane) followed by trituration with MeOH, DCM and *n*‐pentane (1 : 2 : 7) to afford compound **25** (0.026 g, 17 %) as white solid. 1H NMR (400 MHz, DMSO‐d_6_): δ 10.53 (s, 1H), 8.87 (d, *J*=3.2 Hz, 1H), 8.59 (d, *J*=1.8 Hz, 1H), 8.32–8.28 (m, 2H), 8.17 (d, *J*=1.8 Hz, 1H), 8.09 (s, 1H), 7.80 (d, *J*=8.6 Hz, 1H), 7.64 (dd, *J*=4.0 Hz, 8.4 Hz, 1H), 7.26 (d, *J*=8.6 Hz, 1H), 3.03 (s, 3H), 1.94 (s, 3H). HPLC purity: 99.98 %; LCMS m/z: 382.17 [M+H]^+^, t_R_=2.34 min.

*N‐(5‐Chloropyridin‐3‐yl)‐2,3‐dimethyl‐4‐oxo‐1,2,3,4‐tetrahydropyrido[2,3‐h]quinazoline‐2‐carboxamide* (**26**). Selenium powder (0.126 g, 1.594 mmol, 30 mol %) was taken into a nitrogen‐filled dry round bottom flask. Then 6‐methyl‐5‐nitroquinoline (1.0 g, 5.31 mmol), nitrobenzene (0.109 mL, 1.06 mmol, 20 mol %), water (0.19 mL, 10.63 mmol), MeOH (4 mL) and DMSO (0.8 mL) were added successively. The mixture was heated at 90 °C, NaOH (0.425 g, 10.63 mmol) was added. MeOH (4 mL) added dropwise over 30 min and heating was continued for another 3 h. Then the mixture was concentrated under reduced pressure, a solution of 2 N NaOH (aq., 50 mL) was added to the residue, washed with methyl t‐butyl ether (2 x). The aqueous layer was acidified by 10 % aqueous citric acid solution and then extracted with ethyl acetate (3 x). The combined organic phase was dried over anhydrous sodium sulfate, concentrated under reduced pressure to obtain the intermediate 5‐aminoquinoline‐6‐carboxylic acid (0.270 g, 27 %) as yellowish solid which was used for the next step without further purification. ^1^H NMR (400 MHz, DMSO‐d_6_): δ 12.49 (brs, 1H), 8.88 (d, *J*=2.8 Hz, 1H), 8.76 (d, *J*=8.6 Hz, 1H), 8.08–7.82 (m, 3H), 7.50–7.46 (m, 1H), 7.08 (d, *J*=9.2 Hz, 1H); LCMS (ESI) m/z: 189.07 [M+H]^+^. To a solution of 5‐aminoquinoline‐6‐carboxylic acid (0.750 g, 3.985 mmol) in DMF (10 mL) at 0 °C were successively added EDC.HCl (1.146 g, 5.97 mmol), HOBt (0.646 g, 4.78 mmol) and DIPEA (3.47 mL, 19.9 mmol). The mixture was stirred for 10 min under nitrogen, and thereafter methylamine hydrochloride (0.538 g, 7.97 mmol) was added and stirring was continued at room temperature overnight. The mixture was then diluted with EtOAc, washed with cold water (3 x) and brine. The organic phase was dried over anhydrous sodium sulfate, filtered and concentrated under reduced pressure. The residue was triturated with EtOAc and *n*‐pentane (3 : 7) to obtain the intermediate 5‐amino‐N‐methylquinoline‐6‐carboxamide (0.260 g, 32 %) as off‐white solid. ^1^H NMR (400 MHz, DMSO‐d_6_): δ 8.83 (d, *J*=2.8 Hz, 1H), 8.69 (d, *J*=8.6 Hz, 1H), 8.31 (brs, 1H), 7.85–7.72 (m, 3H), 7.44 (dd, *J*=8.4 Hz, 4.0 Hz, 1H), 7.10 (d, *J*=8.8 Hz, 1H), 2.78 (d, *J*=4.0 Hz, 3H); LCMS (ESI) m/z: 202.10 [M+H]^+^. To a stirred solution of 5‐amino‐N‐methylquinoline‐6‐carboxamide (0.070 g, 0.348 mmol) in AcOH (2.0 mL) was added N‐(5‐chloropyridin‐3‐yl)‐2‐oxopropanamide (0.069 g, 0.348 mmol, 1.0 eq.) and the mixture was refluxed for 3 h. The reaction mixture was cooled to room temperature and concentrated under reduced pressure and co‐evaporated with toluene (2 x). The crude residue was purified by flash column chromatography (70–100 % EtOAc in hexane) followed by trituration with MeOH, DCM and *n*‐pentane (1 : 2 : 7) to afford compound **26** (0.012 g, 9 %) as brown solid. ^1^H NMR (400 MHz, DMSO‐d_6_): δ 10.54 (s, 1H), 8.92 (d, *J*=4.0 Hz, 1H), 8.78 (d, *J*=8.0 Hz, 1H), 8.71 (s, 1H), 8.32 (s, 1H), 8.19 (s, 1H), 7.98 (d, *J*=8.6 Hz, 1H), 7.91 (s, 1H), 7.59–7.52 (m, 1H), 7.37 (d, *J*=8.8 Hz, 1H), 3.03 (s, 3H), 1.87 (s, 3H); HPLC purity: 92.15 %; LCMS m/z: 382.14 [M+H]^+^, t_R_=2.21 min.

*8‐Chloro‐5‐methyl‐5,6‐dihydroimidazo[1,2‐c]quinazoline‐5‐carboxylic acid* (**43**). 5‐Chloro‐2‐(1*H*‐imidazol‐2‐yl)aniline (30 mg, 0.155 mmol) was reacted according to General procedure A to afford compound **43** as an off‐white solid (20 mg, 49 %). ^1^H NMR (400 MHz, DMSO‐d_6_) δ 13.5 (br s, 1H), 7.70–7.57 (m, 2H), 7.42 (s, 1H), 7.06 (s, 1H), 6.90 (s, 1H), 6.80 (d, *J*=7.6 Hz, 1H), 1.95 (s, 3H) ppm. LCMS (ESI) m/z: 264.13 [M+H]^+^.

*8‐Chloro‐N‐(5‐chloropyridin‐3‐yl)‐5‐methyl‐5,6‐dihydroimidazo[1,2‐c]quinazoline‐5‐carboxamide* (**27**). Compound **43** (200 mg, 0.758 mmol) was reacted with 5‐chloropyridin‐3‐amine (117 mg, 0.91 mmol) according to General procedure B and the crude product was purified via reverse phase preparative HPLC to afford compound **27** as an off‐white solid (30 mg, 11 %). ^1^H NMR (400 MHz, DMSO‐d_6_): *δ* 10.39 (s, 1H), 8.71 (s, 1H), 8.36 (s, 1H), 8.17 (s, 1H), 7.68 (d, *J*=8.0 Hz, 1H), 7.57 (s, 1H), 7.44 (s, 1H), 7.12 (s, 1H), 6.94 (s, 1H), 6.89 (d, *J*=8.0 Hz, 1H), 2.00 (s, 3H) ppm. ^13^C NMR (75 MHz, DMSO‐d_6_) δ 169.6, 143.2, 141.6, 140.2, 139.6, 135.8, 134.1, 130.5, 127.6, 126.2, 124.5, 119.5, 117.5, 114.6, 111.9, 74.3, 23.7 ppm. HRMS m/z: [M+H]^+^ calcd for C_17_H_13_Cl_2_N_5_O 374.0571; Found 374.0575.

*8‐Chloro‐N‐(5‐chloropyridin‐3‐yl)‐5‐methyl‐2,3,5,6‐tetrahydroimidazo[1,2‐c]quinazoline‐5‐carboxamide* (**28**). 5‐Chloro‐2‐(4,5‐dihydro‐1H‐imidazol‐2‐yl)aniline (100 mg, 0.511 mmol) was reacted with compound **39** (122 mg, 0.613 mmol) according to General procedure A to afford compound **28** as a white solid (48 mg, 25 %). ^1^H NMR (400 MHz, DMSO‐d_6_) δ 10.57 (brs, 1H), 8.79 (s, 1H), 8.32 (s, 1H), 8.26 (t, *J*=1.8 Hz, 1H), 7.70 (brs, 1H), 7.64 (d, *J*=8.4 Hz, 1H), 6.78 (d, *J*=1.8 Hz, 1H), 6.72 (d, *J*=8.4 Hz, 1H), 3.81–3.72 (m, 2H), 3.40–3.29 (m, 2H), 1.62 (s, 3H) ppm. ^13^C NMR (75 MHz, DMSO‐d_6_) δ 170.6, 156.5, 144.8, 142.6, 139.8, 136.5, 136.1, 130.4, 127.7, 126.2, 117.7, 114.0, 110.8, 72.7, 52.9, 46.1, 20.7 ppm. HRMS m/z: [M+H]^+^ calcd for C_17_H_15_Cl_2_N_5_O 376.0728; Found 376.0733.

*7‐Bromo‐N‐(5‐chloropyridin‐3‐yl)‐2,3‐dimethyl‐4‐oxo‐1,2,3,4‐tetrahydroquinazoline‐2‐carboxamide* (**44**). 2‐Amino‐4‐bromo‐*N*‐methylbenzamide (500 mg, 2.52 mmol) was reacted with compound **39** (749 mg, 3.27 mmol) according to General procedure A to afford compound **44** as a light yellow solid (600 mg, 58 %). ^1^H NMR (400 MHz, DMSO‐d_6_) δ 10.41 (s, 1H), 8.75 (s, 1H), 8.36 (d, *J*=2.0 Hz, 1H), 8.22 (s, 1H), 7.59–7.56 (m, 1H), 7.48 (s, 1H), 6.97–6.84 (m, 2H), 2.93 (s, 3H), 1.72 (s, 3H) ppm. LCMS (ESI) m/z: 408.83 [M+H]^+^.

*N‐(5‐Chloropyridin‐3‐yl)‐2,3‐dimethyl‐4‐oxo‐7‐(pyridin‐3‐yl)‐1,2,3,4‐tetrahydroquinazoline‐2‐carboxamide* (**29**). To a solution of compound **44** (200 mg, 0.488 mmol) in 1,4‐dioxane and water (4:0.01) in a MW tube was added pyridin‐3‐ylboronic acid (90 mg, 0.732 mmol), K_3_PO_4_ (415 mg, 1.95 mmol). The mixture was degassed with N_2_ for 10 min, then PdCl_2_(dppf).DCM (8 mg, 0.01 mmol, 2 mol %) was added. The reaction was then heated under MW irradiation at 100 °C for 1 h. Upon completion the reaction mixture was cooled to rt, diluted with EtOAc, then washed with water and brine. The organic layer was then dried over anhyd. Na_2_SO_4_, filtered and concentrated *in vacuo*. The crude product was then purified via FCC to afford compound **29** as a grey solid (31 mg, 16 %). ^1^H NMR (400 MHz, DMSO‐d_6_) δ 10.41 (s, 1H), 8.84 (s, 1H), 8.74 (s, 1H), 8.59 (d, *J*=4.0 Hz, 1H), 8.34 (s, 1H), 8.23 (s, 1H), 8.02 (d, *J*=7.6 Hz, 1H), 7.78 (d, *J*=8.4 Hz, 1H), 7.53–7.47 (m, 1H), 7.39 (s, 1H), 7.12 (dd, *J*=8.0 Hz, 1H), 7.03 (s, 1H), 2.99 (s, 3H), 1.77 (s, 3H) ppm. ^13^C NMR (75 MHz, DMSO‐d_6_) δ 171.0, 162.4, 149.1, 147.6, 145.5, 142.9, 141.8, 139.6, 136.1, 135.0, 134.2, 130.5, 128.6, 126.1, 123.9, 117.1, 114.3, 112.6, 75.7, 28.9, 22.1 ppm. HRMS m/z: [M+H]^+^ calcd for C_21_H_18_ClN_5_O_2_ 408.1223; Found 408.1223.

*2‐Amino‐4‐(6‐methoxypyridin‐3‐yl)‐N‐methylbenzamide* (**45**). To a solution of 2‐amino‐4‐bromo‐*N*‐methylbenzamide (0.120 g, 0.524 mmol,) in 1,4‐dioxane and water (2:0.02) in a MW tube was added 6‐methoxypyridin‐3‐yl)boronic acid (0.120 g, 0.786 mmol), K_3_PO_4_ (415 mg, 1.95 mmol). The mixture was degassed with N_2_ for 10 min, then PdCl_2_(dppf).DCM (8 mg, 0.01 mmol, 2 mol %) was added. The reaction was then heated under microwave irradiation at 100 °C for 1 h. Upon completion the reaction mixture was cooled to rt, diluted with EtOAc, then washed with water and brine. The organic layer was then dried over anhyd. Na_2_SO_4_, filtered and concentrated *in vacuo*. The crude product was then purified via FCC to afford compound **45** as a brown gum (100 mg, 74 %). ^1^H NMR (400 MHz, DMSO‐d_6_): δ 8.42 (s, 1H), 8.20 (s, 1H), 7.93 (d, *J*=8.4 Hz, 1H), 7.62–7.48 (m, 2H), 6.95–6.86 (m, 2H), 6.80 (d, *J*=8.4 Hz, 1H), 6.61–6.59 (m, 2H), 3.89 (s, 3H), 2.74 (s, 3H) ppm. LCMS (ESI) m/z: 258.12 [M+H]^+^.

*N‐(5‐Chloropyridin‐3‐yl)‐7‐(6‐methoxypyridin‐3‐yl)‐2,3‐dimethyl‐4‐oxo‐1,2,3,4‐tetrahydroquinazoline‐2‐carboxamide* (**30**). Compound **45** (500 mg, 2.52 mmol) was reacted with compound **39** (749 mg, 3.27 mmol) according to General procedure A to afford compound **30** as an off‐white solid (26 mg, 15 %). ^1^H NMR (400 MHz, DMSO‐d_6_) δ 10.41 (s, 1H), 8.73 (s, 1H), 8.43 (s, 1H), 8.32 (s, 1H), 8.22 (s, 1H), 7.99–7.89 (m, 1H), 7.72 (d, *J*=4.8 Hz, 1H), 7.34 (s, 1H), 7.08–7.00 (m, 1H), 6.97–6.83 (m, 2H), 3.88 (s, 3H), 2.96 (s, 3H), 1.74 (s, 3H) ppm. ^13^C NMR (75 MHz, DMSO‐d_6_) δ 171.1, 163.4, 162.5, 145.5, 144.8, 142.9, 141.7, 139.5, 137.5, 136.2, 130.5, 128.6, 128.5, 126.0, 116.7, 113.8, 111.8, 110.7, 75.7, 53.3, 28.9, 22.1 ppm. HRMS m/z: [M+H]^+^ calcd for C_22_H_20_ClN_5_O_3_ 438.1329; Found 438.1329.

*N‐(5‐Chloropyridin‐3‐yl)‐2,3‐dimethyl‐7‐(1‐methyl‐1H‐pyrazol‐3‐yl)‐4‐oxo‐1,2,3,4‐tetrahydroquinazoline‐2‐carboxamide* (**31**). To a stirred solution of compound **44** (140 mg, 0.342 mmol) in 1,4‐dioxane (4 mL) in a MW tube was added (1‐methyl‐1*H*‐pyrazol‐3‐yl)boronic acid (84 mg, 0.411 mmol), K_3_PO_4_ (145 mg, 0.685 mmol). The mixture was degassed with N_2_ for 10 min, then PdCl_2_(dppf).DCM (14 mg, 0.017 mmol, 5 mol %) was added. The reaction was then heated under MW irradiation at 100 °C for 1 h. Upon completion the reaction mixture was cooled to rt, diluted with EtOAc, then washed with water and brine. The organic layer was then dried over anhyd. Na_2_SO_4_, filtered and concentrated *in vacuo*. The crude product was then purified via FCC to afford compound **31** as an off‐white solid (50 mg, 25 %). ^1^H NMR (400 MHz, DMSO‐d_6_): *δ* 10.40 (s, 1H), 8.74 (s, 1H), 8.34 (s, 1H), 8.23 (s, 1H), 7.74 (s, 1H), 7.67 (d, *J*=8.0 Hz, 1H), 7.25–7.17 (m, 3H), 6.64 (s, 1H), 3.84 (s, 3H), 2.99 (s, 3H), 1.76 (s, 3H) ppm. ^13^C NMR (75 MHz, DMSO‐d_6_) δ 171.2, 162.5, 149.2, 145.3, 142.9, 139.5, 137.9, 136.1, 132.5, 130.5, 128.1, 126.1, 115.6, 113.8, 110.5, 103.1, 75.7, 38.7, 28.8, 22.0 ppm. HRMS m/z: [M+H]^+^ calcd for C_20_H_19_ClN_6_O_2_ 411.1332; Found 411.1332.

*N‐(5‐Chloropyridin‐3‐yl)‐2,3‐dimethyl‐7‐(1‐methyl‐1H‐pyrazol‐4‐yl)‐4‐oxo‐1,2,3,4‐tetrahydroquinazoline‐2‐carboxamide* (**32**). To a solution of compound **44** (120 mg, 0.293 mmol) in 1,4‐dioxane and water (9 : 1) in a MW tube was added (1‐methyl‐1*H*‐pyrazol‐4‐yl)boronic acid (44 mg, 0.352 mmol), K_3_PO_4_ (187 mg, 0.88 mmol). The mixture was degassed with N_2_ for 10 min, then PdCl_2_(dppf).DCM (12 mg, 0.015 mmol, 5 mol %) was added. The reaction was then heated under microwave irradiation at 100 °C for 1 h. Upon completion the reaction mixture was cooled to rt, diluted with EtOAc, then washed with water and brine. The organic layer was then dried over anhyd. Na_2_SO_4_, filtered and concentrated *in vacuo*. The crude product was then purified via FCC to afford compound **32** as an off‐white solid (35 mg, 29 %). ^1^H NMR (400 MHz, DMSO‐d_6_) δ 10.40 (s, 1H), 8.74 (s, 1H), 8.34 (s, 1H), 8.23 (s, 1H), 8.11 (s, 1H), 7.81 (s, 1H), 7.64 (d, 1H, *J*=8.0 Hz), 7.20 (s, 1H), 6.98 (d, 1H, *J*=8.0 Hz), 6.86 (s, 1H), 3.86 (s, 3H), 2.95 (s, 3H), 1.73 (s, 3H) ppm. ^13^C NMR (75 MHz, DMSO‐d_6_) δ 171.2, 162.5, 145.5, 142.9, 139.5, 137.4, 136.3, 136.1, 130.5, 128.4, 128.4, 126.0, 121.2, 115.6, 112.7, 110.0, 75.7, 38.7, 28.8, 22.0 ppm. HRMS m/z: [M+H]^+^ calcd for C_20_H_19_ClN_6_O_2_ 411.1332; Found 411.1335.

*N‐(5‐Chloropyridin‐3‐yl)‐2,3‐dimethyl‐4‐oxo‐7‐(1H‐pyrazol‐1‐yl)‐1,2,3,4‐tetrahydroquinazoline‐2‐carboxamide* (**33**). To a stirred solution of compound **44** (200 mg, 0.488 mmol) in 1,4‐dioxane (4 mL) was added Cs_2_CO_3_ (318 mg, 0.976 mmol) and 1*H*‐pyrazole (40 mg, 0.586 mmol). The mixture was then degassed with N_2_ for 10 min and then *t*BuXPhos‐Pd−G3 (236 mg, 0.111 mmol) was added. The vessel was sealed and heated at 110 °C for 5 h. Upon completion, the reaction mixture was cooled to rt, diluted with EtOAc and washed with water and brine. The organic phase was then dried over anhyd. Na_2_SO_4_, filtered and concentrated *in vacuo*. The crude residue was purified via FCC followed by reverse phase preparative HPLC to afford compound **33** as a white solid (20 mg, 10 %). ^1^H NMR (400 MHz, DMSO‐d_6_): *δ* 10.45 (s, 1H), 8.75 (s, 1H), 8.47 (s, 1H), 8.35 (s, 1H), 8.23 (s, 1H), 7.77–7.74 (m, 2H), 7.50 (s, 1H), 7.26–7.22 (m, 2H), 6.55 (s, 1H), 2.96 (s, 3H), 1.74 (s, 3H) ppm. ^13^C NMR (75 MHz, DMSO‐d_6_) δ 170.8, 162.1, 146.1, 143.2, 143.0, 141.5, 139.6, 136.1, 130.5, 129.3, 128.0, 126.1, 112.4, 108.3, 108.2, 103.6, 75.8, 28.8, 22.0 ppm. HRMS m/z: [M+H]^+^ calcd for C_19_H_17_ClN_6_O_2_ 397.1175; Found 397.1179.

*N‐(5‐Chloropyridin‐3‐yl)‐7‐methoxy‐2,3‐dimethyl‐4‐oxo‐1,2,3,4‐tetrahydroquinazoline‐2‐carboxamide* (**34**). A mixture of 2‐amino‐4‐methoxy‐*N*‐methyl‐benzamide (87 mg, 0.483 mmol) and compound **39** (19.1 mg, 0.0962 mmol) in Eaton's reagent (180 mg, 0.096 mmol) was heated to 70 °C for 35 min. The reaction mixture was then diluted with EtOAc, washed with sat. NaHCO_3_ and brine, dried over MgSO_4_, filtered and concentrated *in vacuo*. The crude product was then purified via FCC to afford compound **34** as a white powder (9 mg, 26 %). ^1^H NMR (300 MHz, DMSO‐d_6_) δ 10.42 (s, 1H), 8.74 (d, *J*=2.1 Hz, 1H), 8.34 (d, *J*=2.2 Hz, 1H), 8.23 (t, *J*=2.2 Hz, 1H), 7.58 (d, *J*=8.7 Hz, 1H), 7.27 (s, 1H), 6.35 (dd, *J*=8.7, 2.4 Hz, 1H), 6.23 (d, *J*=2.4 Hz, 1H), 3.74 (s, 3H), 2.93 (s, 3H), 1.69 (s, 3H) ppm. ^13^C NMR (75 MHz, DMSO‐d_6_) δ 171.2, 163.3, 162.5, 146.7, 142.9, 139.5, 136.1, 130.5, 129.6, 126.0, 108.2, 105.8, 98.1, 75.7, 55.1, 28.7, 21.9 ppm. HRMS m/z: [M+H]^+^ calcd for C_17_H_17_ClN_4_O_3_ 361.1062; Found 361.1055.

*N‐(5‐Chloropyridin‐3‐yl)‐7‐methoxy‐2,3‐dimethyl‐4‐oxo‐1,2,3,4‐tetrahydropyrido[3,2‐d]pyrimidine‐2‐carboxamide* (**35**). To a stirred solution of 3‐amino‐5‐methoxy‐*N*‐methylpicolinamide (140 mg, 0.772 mmol) in PhMe (6 mL) were added compound **39** (184 mg, 0.927 mmol) and *p*TsOH (66 mg, 0.386 mmol). The mixture was heated at reflux for 16 h, and upon completion the reaction mixture was concentrated *in vacuo*. The resultant reside was dissolved in EtOAc, washed with water and brine, dried over Na_2_SO_4_, filtered and concentrated *in vacuo*. The crude product was purified via FCC and then further purified via reverse phase preparative HPLC to afford compound **35** as a white solid (54 mg, 19 %). ^1^H NMR (400 MHz, DMSO‐d_6_) δ 10.44 (s, 1H), 8.36 (s, 1H), 8.23 (s, 1H), 7.75 (s, 1H), 7.44 (s, 1H), 6.61 (s, 1H) 3.82 (s, 3H), 2.93 (s, 3H), 1.71 (s, 3H) ppm. ^13^C NMR (75 MHz, DMSO‐d_6_) δ 170.7, 161.3, 158.1, 143.4, 143.0, 139.7, 136.1, 130.5, 130.2, 126.2, 124.8, 104.0, 75.7, 55.5, 29.0, 22.1 ppm. HRMS m/z: [M+H]^+^ calcd for C_16_H_16_ClN_5_O_3_ 362.1016; Found 362.1019.

*N‐(5‐Chloropyridin‐3‐yl)‐5‐fluoro‐7‐methoxy‐2,3‐dimethyl‐4‐oxo‐1,2,3,4‐tetrahydroquinazoline‐2‐carboxamide* (**36**). 2‐Amino‐6‐fluoro‐4‐methoxy‐*N*‐methylbenzamide (200 mg, 1.01 mmol) was reacted with compound **39** (200 mg, 1.01 mmol) according to General procedure and purified via reverse phase preparative HPLC to afford compound **36** as a white solid (18 mg, 5 %). ^1^H NMR (400 MHz, DMSO‐d_6_) δ 10.32 (s, 1H), 8.73 (d, *J*=1.6 Hz, 1H), 8.35 (d, *J*=2.0 Hz, 1H), 8.22 (s, 1H), 7.49 (s, 1H), 6.17 (d, *J*=9.2 Hz, 1H), 6.09 (s, 1H), 3.74 (s, 3H), 2.91 (s, 3H), 1.70 (s, 3H) ppm. ^13^C NMR (75 MHz, DMSO‐d_6_) δ 170.9, 163.4 (d, *J*=14.7 Hz), 163.1 (d, *J*=257.7 Hz), 159.6 (d, *J*=3.6 Hz), 148.2 (d, *J*=5.9 Hz), 143.0, 139.6, 136.0, 130.5, 126.2, 75.1, 55.5, 28.5, 21.9 ppm. ^19^F NMR (282 MHz, DMSO‐d_6_) δ −110.0 ppm. HRMS m/z: [M+H]^+^ calcd for C_17_H_16_ClFN_4_O_3_ 379.0969; Found 379.0970.

### Biology Experimental

#### P. falciparum LDH Viability Assay

This assay is a platform assay conducted at the Walter and Eliza Hall Institute and uses a protocol previously decsribed.[^30^, ^31^]

#### Human HepG2 Cytotoxicity Assay

This assay uses Cell TitreGlo as a metabolic maker for human HepG2 cell growth and uses a protocol previously described.^[32]^

#### In Vitro ADME

Human liver microsome stability, rat hepatocyte stability, aqueous solubility and eLogD are platform assays conducted by TCG Lifesciences and undertaken using previously described protocols.^[10]^

#### PfATP4 Drug Resistant Parasite Viability Assays

These assays were performed according to a previously described protocol.^[16]^ Briefly, *P. falciparum* Dd2 strains treated with compound were incubated for 72 h at 37 °C. After incubating for 72 h, plates were frozen at −80 °C and then thawed at room temperature for at least 2 h. LDH activity, a surrogate for parasite growth was measured by the addition of the Malstst reagent mix, followed by incubation for 30 min and absorbance read using a ClarioStar Microplate reader at 650 nm. Values were normalized to DMSO control as a percentage and EC_50_ and dose response curves generated using GraphPad Prism.

#### PfATP4 Na^+^ Assay

Membrane ATPase assays were performed using a PiColorLock Gold Phosphate Detection System (Innova Biosciences), essentially as described previously.^[25]^ Membranes were prepared either from isolated 3D7 trophozoites or from isolated Dd2‐Polδ‐PfATP4^G358S^ ^[22]^ and Dd2‐Polδ trophozoites.^[26]^ Reactions were performed at 37 °C for 10 min, and the final reaction mixtures had a pH of 7.2 and consisted of: 150 mM NaCl or choline chloride, 20 mM KCl, 2 mM MgCl_2_, 50 mM Tris, 50 μg/mL (total) protein, 1 mM Na_2_ATP.3H_2_O (MP Biomedicals), and the compound(s) of interest or solvent (DMSO) alone. The final concentration of DMSO in the reactions did not exceed 0.2 % v/v.

#### P. knowlesi and P. falciparum Multidrug‐Resistant Parasite Viability Assay

These assays were performed as previously described.^[16]^ *P. falciparum* 3D7 parasites and resistant lines Dd2, W2mef, 7G8, and artemisinin‐resistant Cambodian isolate (Cam3.I (2539T)), and *P. knowlesi* YH1, were cultured in human O^+^ erythrocytes (RBCs) (Australian Red Cross Blood Service), in RPMI‐HEPES culture medium (pH 7.4, 50 μg/mL hypoxanthine, 25 mM NaHCO_3_, 20 μg/mL gentamicin, 0.5 % Albumax II (GibcoBRL)) (Thermo Fisher Scientific) and maintained in an atmosphere of 1 % O_2_, 4 % CO_2_ and 95 % N_2_. Parasites were synchronised with 5 % (w/v) sorbitol (Sigma‐Aldrich) treatments for ring stages. Growth assays for measuring drug inhibition of in‐cycle, ring to schizont stage parasites have been described previously. Parasites were grown in 1 % haematocrit, at 1 % and 2 % parasitaemia for *P. falciparum* and *P. knowlesi* respectively. Drugs were added at ring stages, and parasite growth was measured at late trophozoite/schizont stages (44–48 h post‐invasion for *P. falciparum*; 24–30 h post‐invasion for *P. knowlesi*). Parasitaemia was measured using a SYBR DNA staining assay on a fluorometer.^[33]^ After the incubation period, supernatants were removed and well contents were resuspended in PBS. An equal volume of SYBR Safe Stain (0.02 % (v/v) in SYBR Lysis buffer (pH 7.5, 20 mM TRIS, 5 mM EDTA, 0.008 % Saponin (w/v), 0.08 % Triton X100 (v/v)) was added to wells and mixed. After incubating for 30 min to 1 h, plates were read on a fluorometer (BMG LabTech PHERAstar FS) (excitation, 485 nm; emission, 520 nm). The background (non‐infected RBCs) was subtracted from all samples and drug treatments were normalised against untreated parasites to calculate the percent survival of drug‐treated parasites. IC_50_s were determined for each drug using GraphPad Prism (GraphPad Software) according to the recommended protocol for non‐linear regression of a log‐(inhibitor)‐versus‐response curve.

#### Dual Gamete Formation Assay

The compounds were evaluated as previously described.^[34]^ Briefly, mature *P. falciparum* NF54 stage V gametocytes were exposed to compounds for 48 h at 37 °C in 384 well plates in gametocyte culture medium (RPMI 1640 supplemented with 25 mM HEPES, 50 μg mL^−1^ hypoxanthine, 4.8 g L^−1^ NaHCO_3_, 2 mM L‐glutamine, 5 % pooled type AB serum, 0.5 % Albumax II (Gibco)) under a 1 % O_2_/ 3 % CO_2_/ 96 % N_2_ environment. Gametogenesis was then triggered by the addition of 10 μL ookinete medium (gametocyte culture medium supplemented with 100 μM xanthurenic acid and 0.27 μg mL^−1^ Cy3‐labelled anti‐Pfs25 antibody) to each well at room temperature. Plates were then cooled on a metal block at 4 °C for four min to ensure even cooling and then stabilised for a further 4 min at 28 °C. At 20 min post‐induction, male gametogenesis was recorded in each well by automated brightfield microscopy using a x4 objective lens and 1.5x magnifier (x6 effective magnification). Afterward, plates were incubated in the dark at room temperature for 24 h and then female gametogenesis was recorded in each well by automated fluorescence microscopy (anti‐Pfs25‐positive cells). All experiments were performed in quadruplicate with DMSO and Cabamiquine as negative and positive controls respectively. All data was evaluated in comparison to the positive and negative controls to calculate percentage inhibition of male and female gametocytes, and dose response analysis and EC_50_ calculation were performed using GraphPad Prism.

#### P. berghei Mouse Model

For the model conducted on compound **14**, rodents were performed in strict accordance with the recommendations of the Australian Government and National Health and Medical Research Council Australian code of practice for the care and use of animals for scientific purposes. The protocols were approved by the Animal Welfare Committee at Deakin University (approval no. G14/2020).

Biological assessment of the *in vivo* antimalarial efficacy of compound **14** was assessed using the *P. berghei* rodent malaria 4‐day suppressive test which was conducted as previously described.^[16]^ A stock solution of compound **14** (10×) was made by dissolving the compound in 70 % (v/v) Tween 80 and 30 % (v/v) ethanol in distilled sterile water. Each day, a fresh aliquot of the compound stock was diluted 1/10 in water for administration to mice. 8 week old female mice (Ozgene) on day 0 were infected with 2×10^7^ *P. berghei* ANKA‐infected erythrocytes. At 2 h and days 1, 2, and 3 post‐infection, mice were administered by oral gavage with 20 mg/kg of compound **14** (4 mice) or vehicle control (4 mice). 2 mice were also dosed with artesunate using the same regime at 30 mg/kg. The parasitemia of mice was assessed by visualizing Giemsa‐stained thin blood smears by microscopy and a minimum of 1000 RBCs were counted. To calculate the percent antimalarial activity the following formula was used: 100 ‐ (mean parasitemia treated day 4 post‐infection/mean parasitemia vehicle control day 4 post‐infection)×100. Mice were culled the day following the last oral gavage.

For the model used to evaluate compound **33**, the use of animals was approved by the Walter and Eliza Hall Institute of Medical Research Animal Ethics Committee under approval number 2020.036 and all procedures were conducted in compliance with the animal ethics guidelines to promote the wellbeing of animals used for scientific purposes, National Health and Medical Research Council Australian code for the care and use of animals for scientific purposes, NHMRC 8th Edition 2013. ASMU:Swiss outbred (pathogen‐free), females, 4 weeks, 15–18 g were kept in exhausted ventilated cages (EVC) with corncob bedding, under standard conditions (21 °C +/−3 °C, 40–70 % relative humidity, 12 h/12 h 6 am‐6 pm light/dark cycle) with food and water ad libitum.

The mouse model for compound **33** was conducted as previously described.^[35]^ Briefly, ‘donor’ female Swiss mice were infected intraperitoneally (IP) with blood stage *P. berghei* parasites constitutively expressing GFP (*P. berghei* ANKA GFPcon 259cl2).^[36]^ Three days later, groups of 3 ‘acceptor’ Swiss mice were infected intravenously with 1 x 10^7^ parasitised red blood cells from the ‘donor mice’. Compound **33** was prepared in a vehicle consisting of 0.5 % carboxymethylcellulose/0.5 % benzylalcohol/0.4 % polysorbate 80 in water. 2 h post‐infection, mice were treated orally once daily on 4 consecutive days (q.d. regimen, once a day) with a dose of compound **33** (20 mpk) or chloroquine (10 mpk). Control mice were left untreated. Peripheral blood samples were taken 24 h after administration of the last dose, and parasitemia was measured by flow cytometry (proportion of GFP‐positive cells in 100,000 recorded events using Attune Nxt, ThermoFisher) and microscopic analysis of Giemsa‐stained blood smears. Parasitemia values were averages of 3 mice per group.

## Supporting Information Summary

Supporting Information contains asexual and sexual, and drug resistant parasite dose response data, *P. berghei* mouse model data, and compound spectra.

## Conflict of Interests

The authors declare no conflict of interest.

1

## Supporting information

As a service to our authors and readers, this journal provides supporting information supplied by the authors. Such materials are peer reviewed and may be re‐organized for online delivery, but are not copy‐edited or typeset. Technical support issues arising from supporting information (other than missing files) should be addressed to the authors.

Supporting Information

## Data Availability

The data that support the findings of this study are available from the corresponding author upon reasonable request.
