# Supplementary material for: Lactam Truncation Yields a Dihydroquinazolinone Scaffold with Potent Antimalarial Activity that Targets PfATP4
Source: ChemMedChem. 2024 Oct 29;19(24):e202400549. doi: 10.1002/cmdc.202400549 (PMC11648822; doi:10.1002/cmdc.202400549)
Supplement: Supplementary file 1 — Supporting Information [file CMDC-19-e202400549-s001.pdf]

ChemMedChem

Supporting Information

Lactam Truncation Yields a Dihydroquinazolinone Scaffold with Potent Antimalarial Activity that Targets PfATP4

Trent D. Ashton, Petar P. S. Calic, Madeline G. Dans, Zi Kang Ooi, Qingmiao Zhou, Katie Loi, Kate E. Jarman, Josephine Palandri, Deyun Qiu, Adele M. Lehane, Bikash Maity, Nirupam De, Mufuliat T. Famodimu, Michael J. Delves, Emma Y. Mao, Maria R. Gancheva, Danny W. Wilson, Mrittika Chowdury, Tania F. de Koning-Ward, Delphine Baud, Stephen Brand, Paul F. Jackson, Alan F. Cowman, and Brad E. Sleebs*

SUPPORTING INFORMATION FOR

Lactam Truncation Yields a Dihydroquinazolinone Scaffold with Potent Antimalarial Activity that Targets PfATP4

Trent D. Ashton,[a,b] Petar P. S. Calic,[a,b] Madeline G. Dans,[a,b] Zi Kang Ooi,[a] Qingmiao Zhou,[a] Katie Loi,[a,b] Kate E. Jarman,[a,b] Josephine Palandri,[a,b] Deyun Qiu,[c] Adele M. Lehane,[c] Bikash Maity,[d] Nirupam De,[d] Mufuliat T. Famodimu,[e] Michael J. Delves,[e] Emma Y. Mao,[f] Maria R. Gancheva,[f] Danny W. Wilson,[f] Mrittika Chowdury,[g,h] Tania F. de Koning-Ward,[g,h] Delphine Baud,[g] Stephen Brand,[g] Paul F. Jackson,[h] Alan F. Cowman,[a,b] and Brad E. Sleebs.[a,b,*]

[a] The Walter and Eliza Hall Institute of Medical Research, Parkville 3052, Australia.

[b] Department of Medical Biology, The University of Melbourne, Parkville 3010, Australia.

[c] Research School of Biology, Australian National University, Canberra, 2601, Australia.

[d] TCG Lifesciences, Kolkata, West Bengal, 700091, India.

[e] Department of Infection Biology, London School of Hygiene and Tropical Medicine, London, WC1E 7HT, UK.

[f] Research Centre for Infectious Diseases, School of Biological Sciences, University of Adelaide, Adelaide, Australia.

[g] School of Medicine, Deakin University, Waurn Ponds, Victoria 3216, Australia

[h] Institute for Mental and Physical Health and Clinical Translation, Deakin University, Geelong, Victoria, 3216, Australia.

[i] Medicines for Malaria Venture, ICC, Route de Pré-Bois 20, 1215 Geneva, Switzerland.

[j] Emerging Science & Innovation, Discovery Sciences, Janssen R&D LLC, La Jolla, California 92121, USA.

[*] Correspondence to:
Brad E. Sleebs
The Walter and Eliza Hall Institute of Medical Research
1G Royal Parade, Parkville 3052, Victoria, Australia
Email: sleebs@wehi.edu.au

Index

Page

S3	Figure S1	*P. falciparum* 3D7 asexual dose response curves.
S4	Figure S2	**49** resistant *P. falciparum* dose response curves.
S5	Figure S3	Mutant PfATP4 strain dose response curves.
S6	Figure S4	*P. knowlesi* YH1 dose response curves.
S7	Figure S5	Multidrug resistant strain dose response curves.
S8	Figure S6	Daul gamete formation assay dose response curves.
S9	Figure S7	*P. berghei* mouse model data.
S10		HPLC trace of final compounds.
S23		^{1}H-NMR spectra of final compounds.

Compound **11**

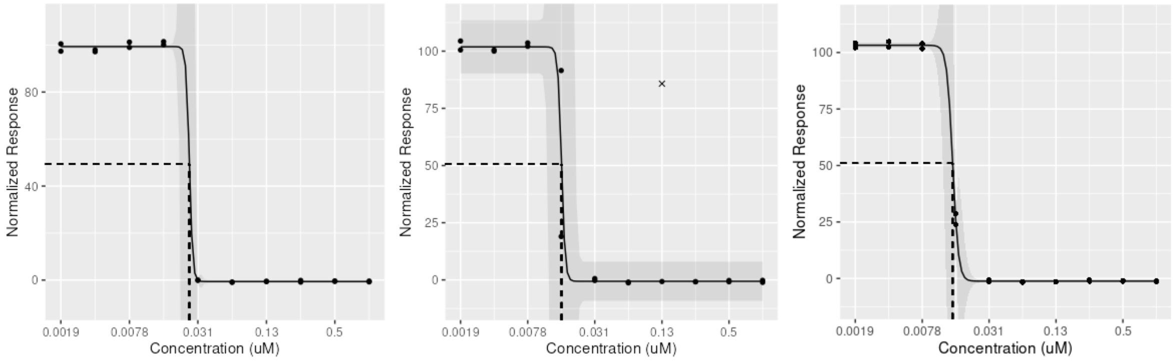

Compound **14**

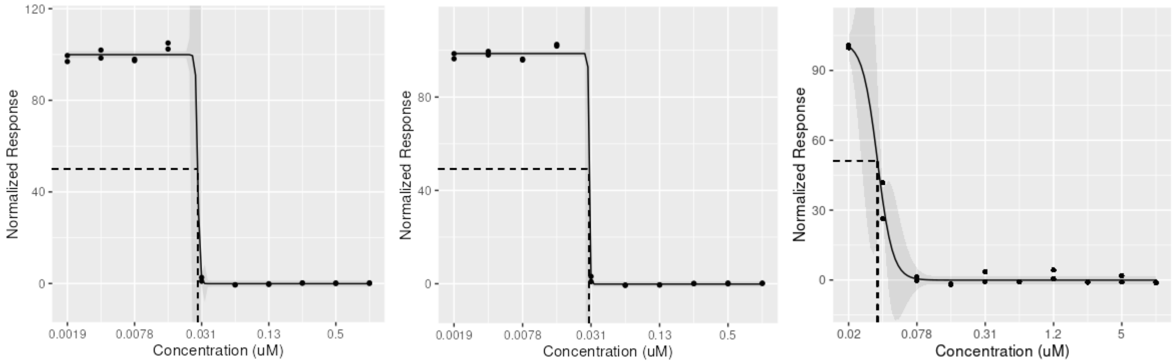

Compound **33**

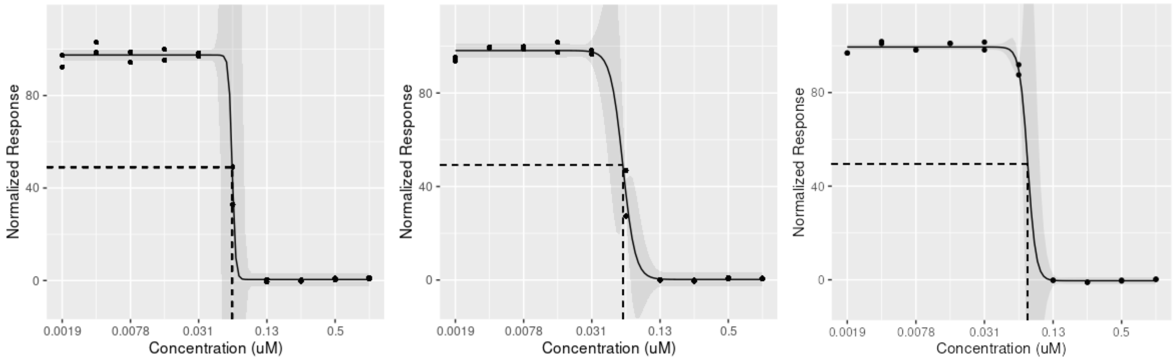

Compound **36** (WJM062)

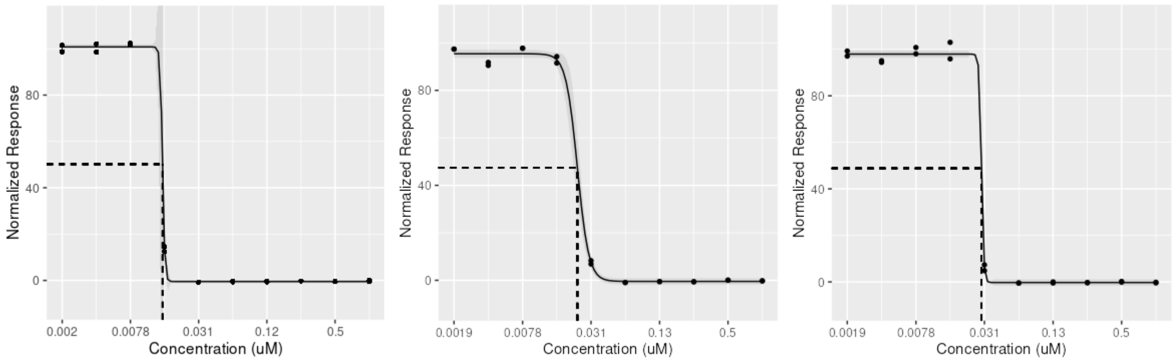

Figure S1. Dose response curves of selected compounds against *P. falciparum* 3D7 parasites each represent one independent experiment (in replicate) measuring the LDH activity of *P. falciparum* 3D7 parasites following exposure to compounds for 72 h.

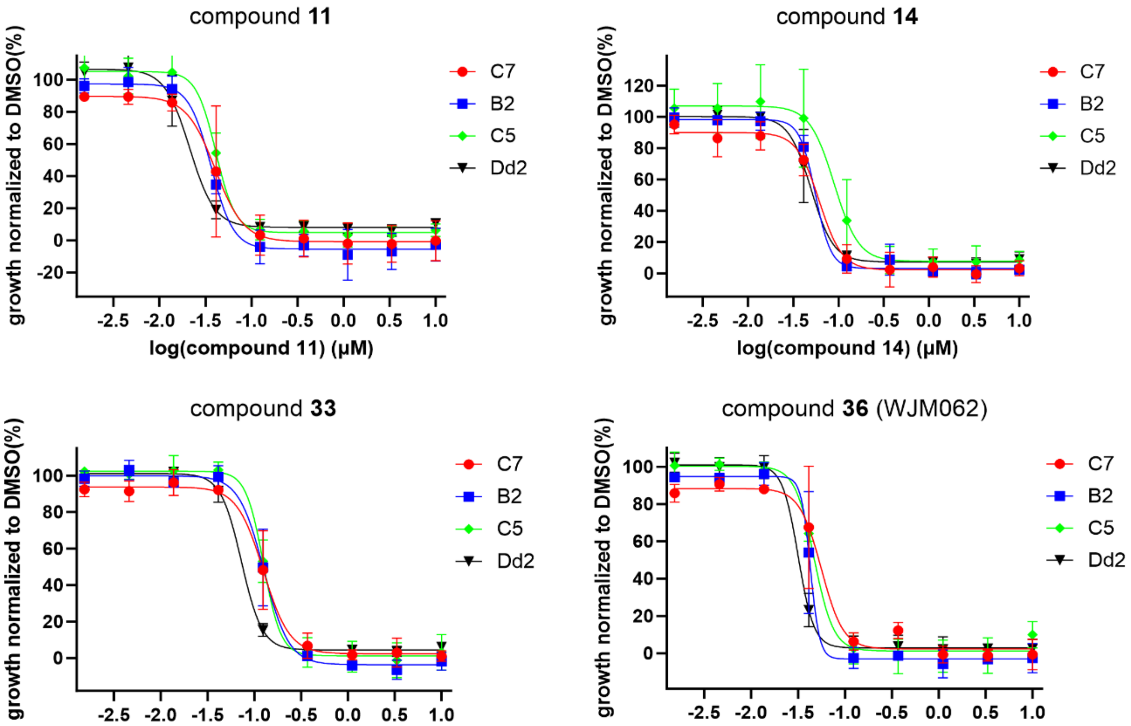

Figure S2. Dose response curves of compounds against **49** resistant *P. falciparum* Dd2 populations from 3 biological replicates in a 72 h Pf LDH assay. Error bars are SD. *P. falciparum* Dd2 **49** resistant populations have the following genetic aberrations: C5: 2.8x CNV in PfATP4; C7: $PfATP4^{F156L}$; B2: $PfATP4^{D425E}$.

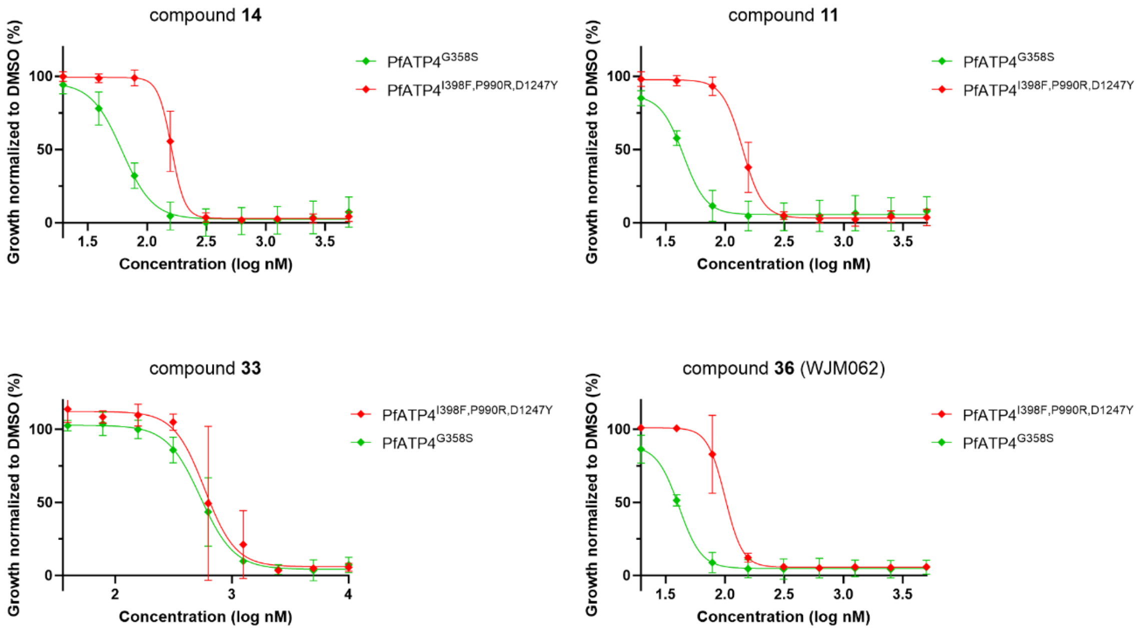

Figure S3. Dose response curves of compounds against *P. falciparum* Dd2 PfATP4$^{I398F, P990R, D1247Y}$ and PfATP4^{G358S} parasites from 3 biological replicates in a 72 h Pf LDH assay. Error bars are SD.

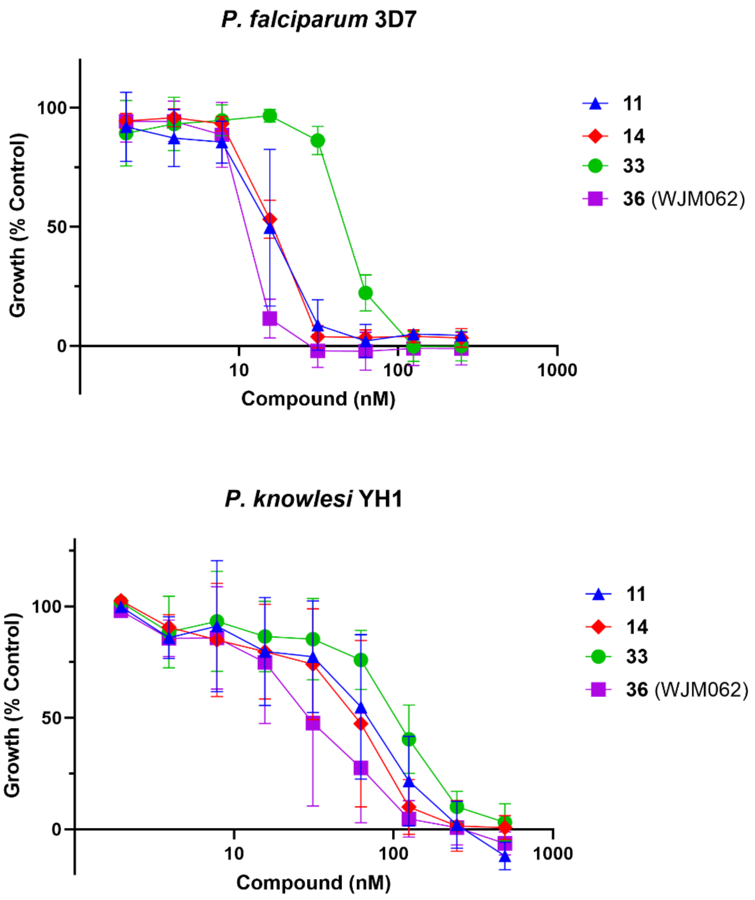

Figure S4. EC_{50} data represent the mean and SD for 3 independent experiments measuring growth of *P. falciparum* parasites over 72 h using SYBR green (top panel) and *P. knowlesi* YH1 parasite growth over 48 h using SYBR green (bottom panel). Error bars are SD.

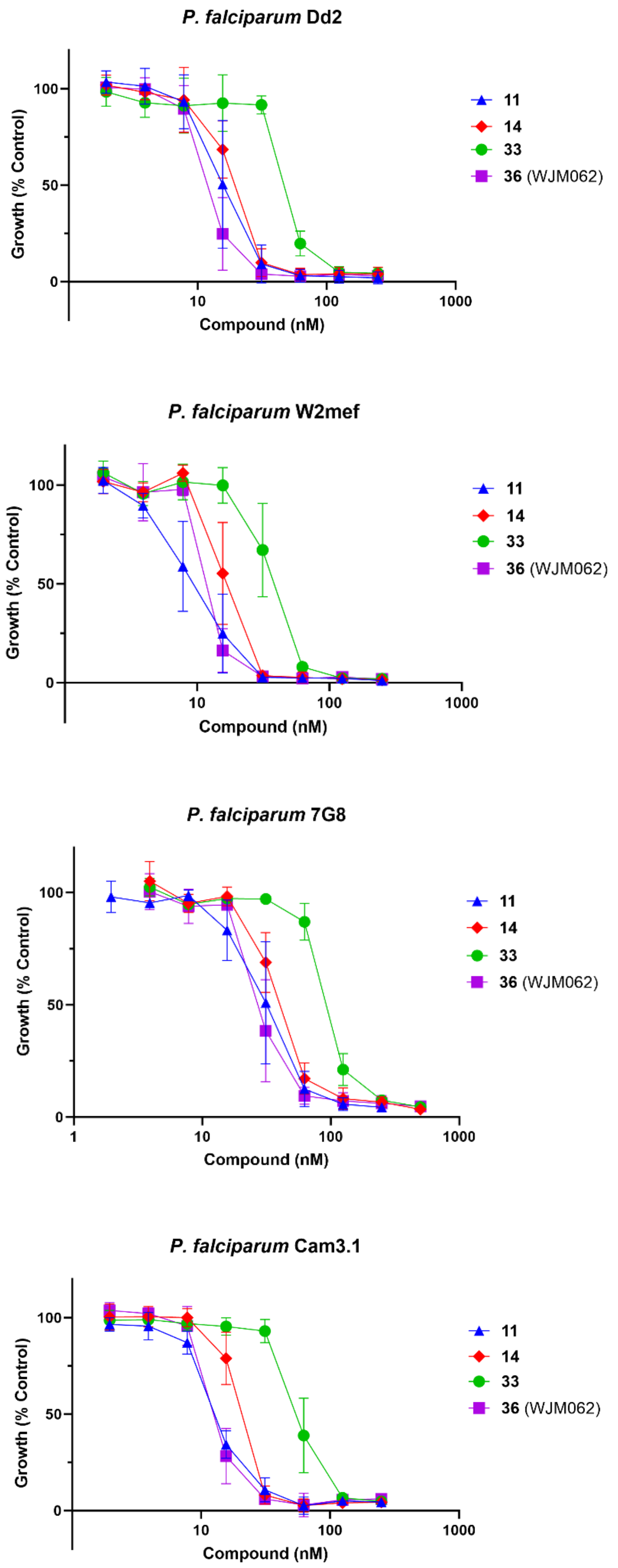

Figure S5. Dose response curves of compounds against *P. falciparum* multidrug resistant strains from 3 biological replicates over 72 h using SYBR green. Error bars are SD.

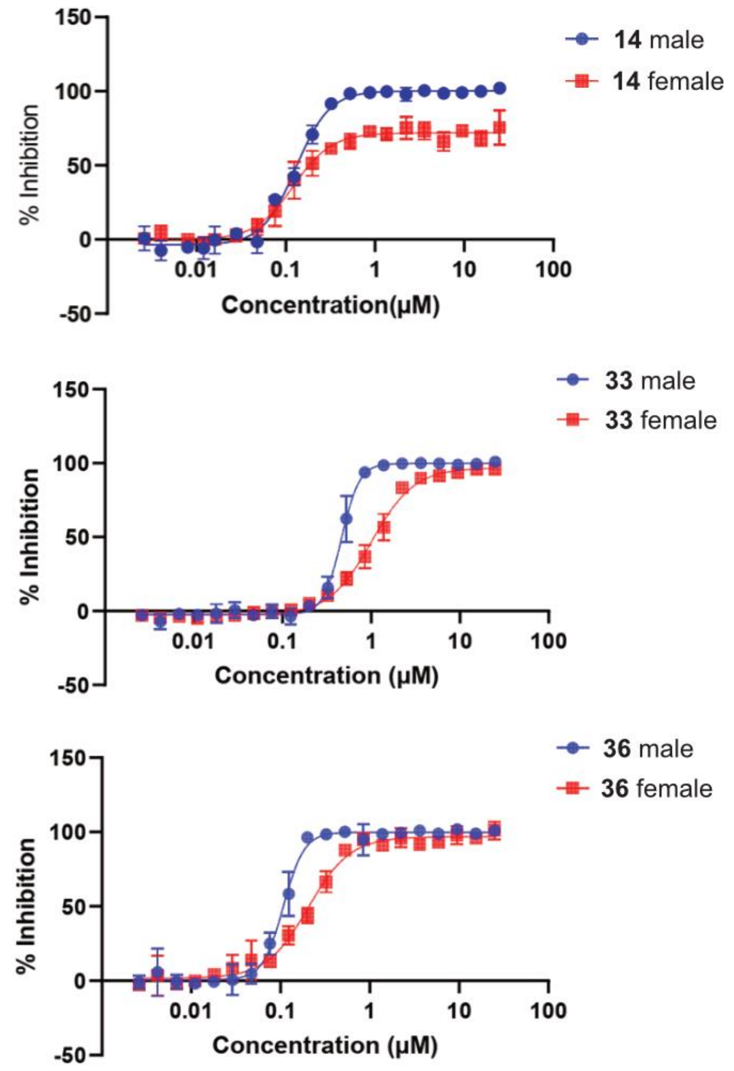

Figure S6. Dose response curves of selected compounds against *P. falciparum* NF54 gamete formation. EC_{50} data represent means and SDs for four replicate experiments following gametocyte exposure to compounds for 48 h prior to inducing gametogenesis. Male gametogenesis was measured 20 min after induction and female gametogenesis was measured 24 h after induction. The viability of male gametes was quantified by automated microscopy measuring exflagellation and for female gametes measuring fluorescence using an αPfs25 antibody.

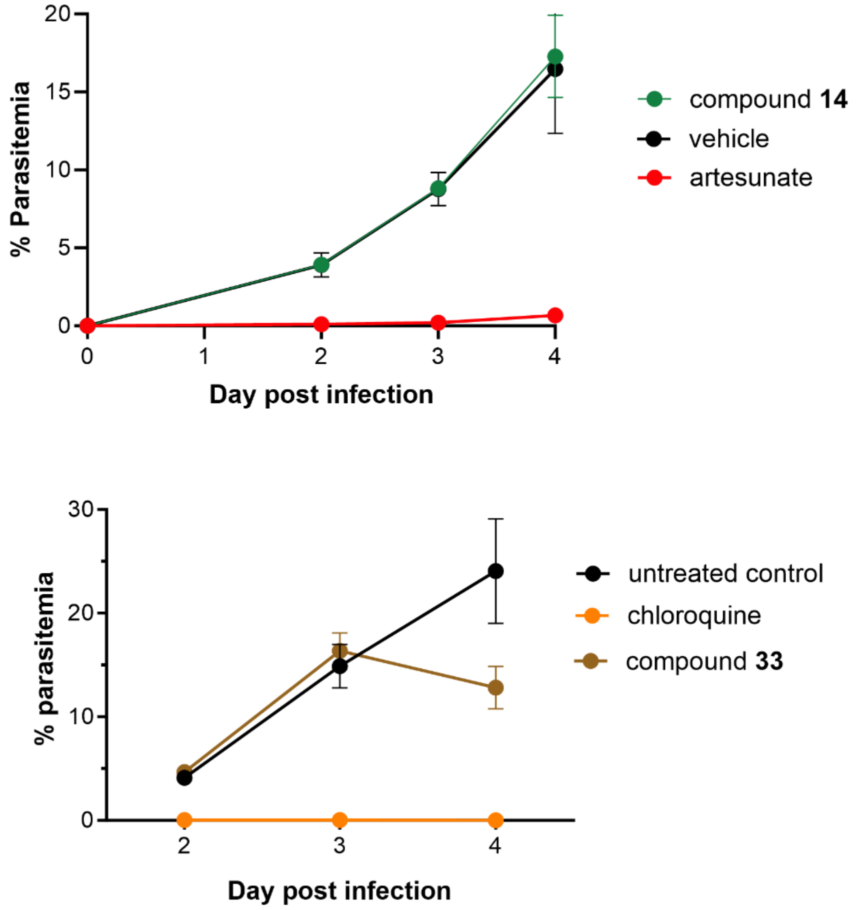

Figure S7. Evaluation of compounds **14** and **33** in two independent *P. berghei* 4 day mouse experiments (top and bottom panels). *P. berghei* ANKA parasites were injected into the tail vein to infect mice on day 0. Compounds **14** and **33** were administered by oral gavage at 20 mg/kg (chloroquine at 10 mg/kg; artesunate at 30 mg/kg) 2 h after infection (day 0) and then on days 1, 2, and 3. Parasitemia was measured on days 2, 3 and 4. Error values are SD.

LC spectra of final compounds.

Compound 11

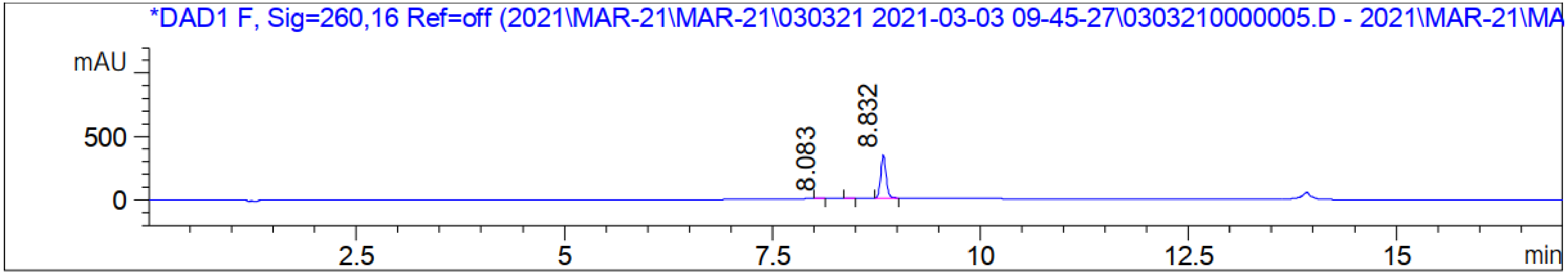

Compound 12

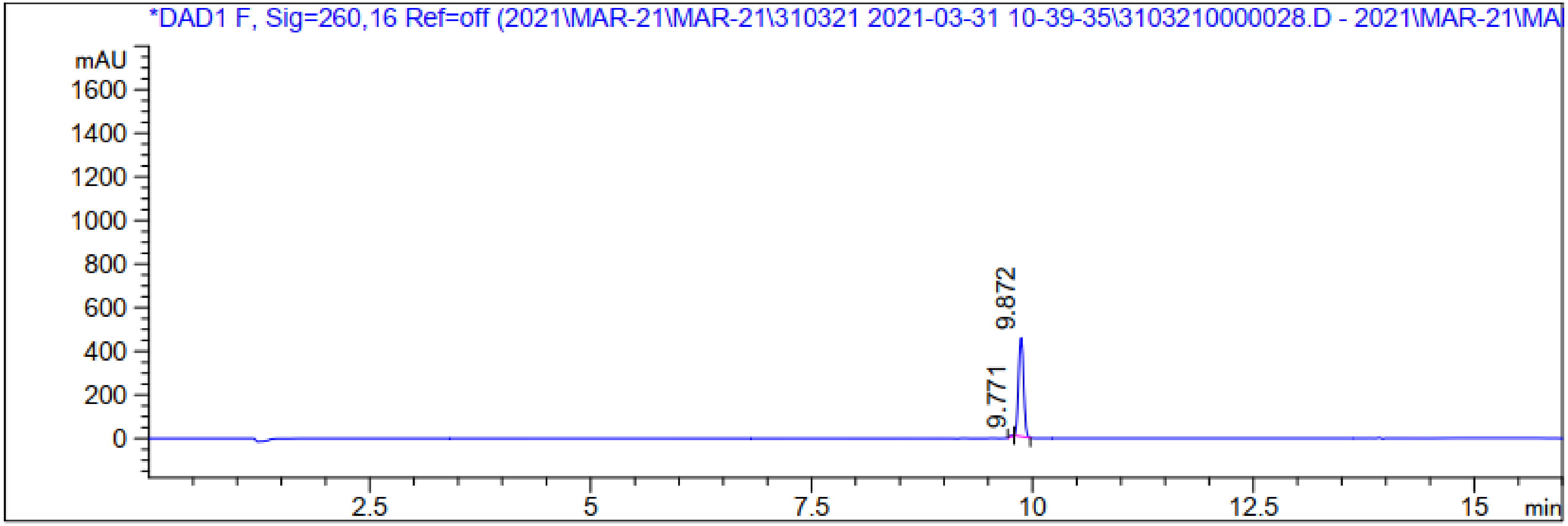

Compound 13

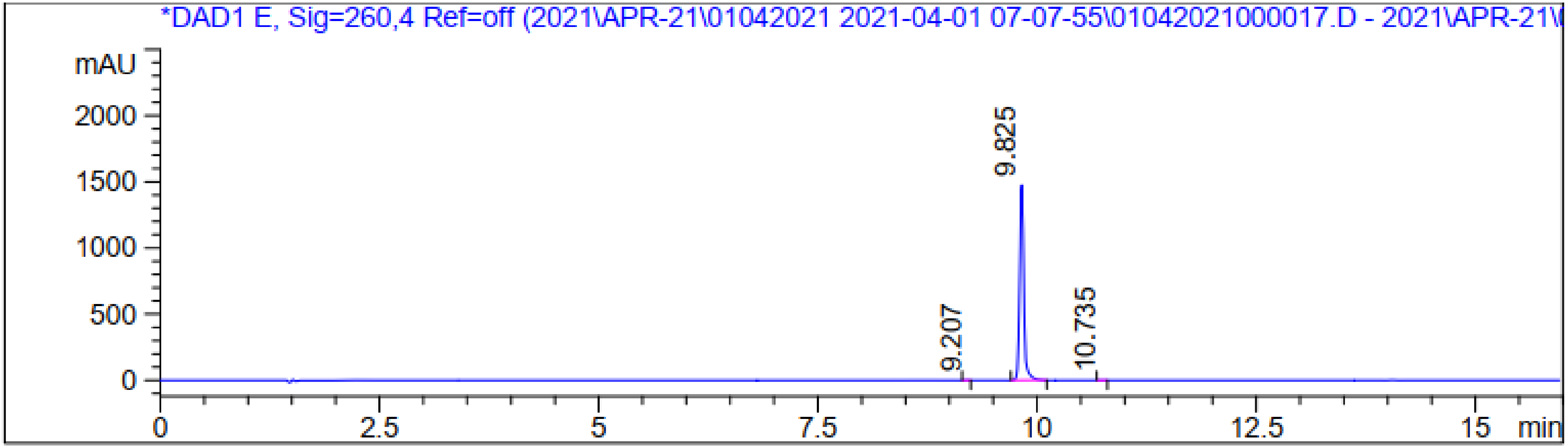

Compound 14

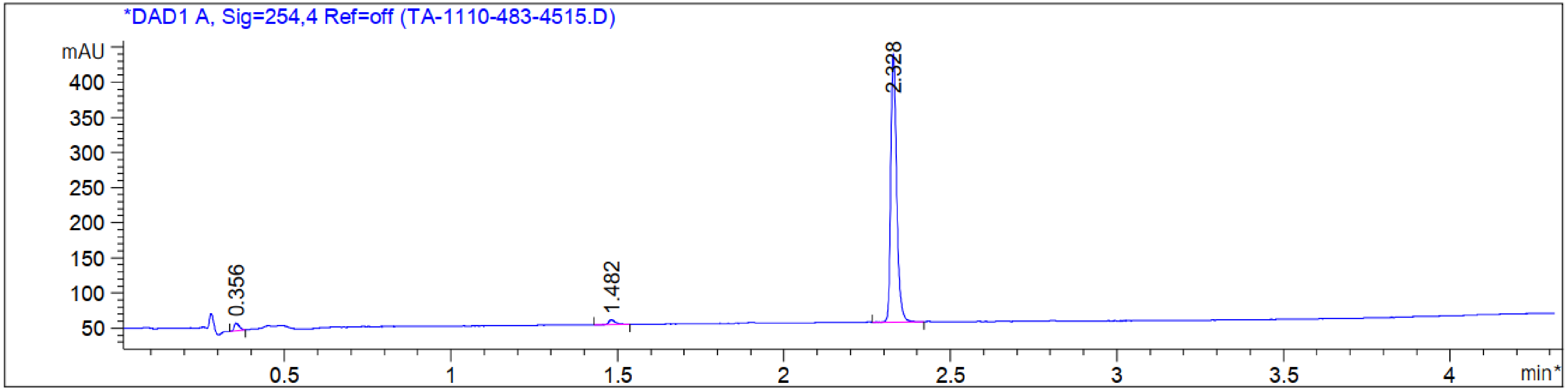

Compound 15

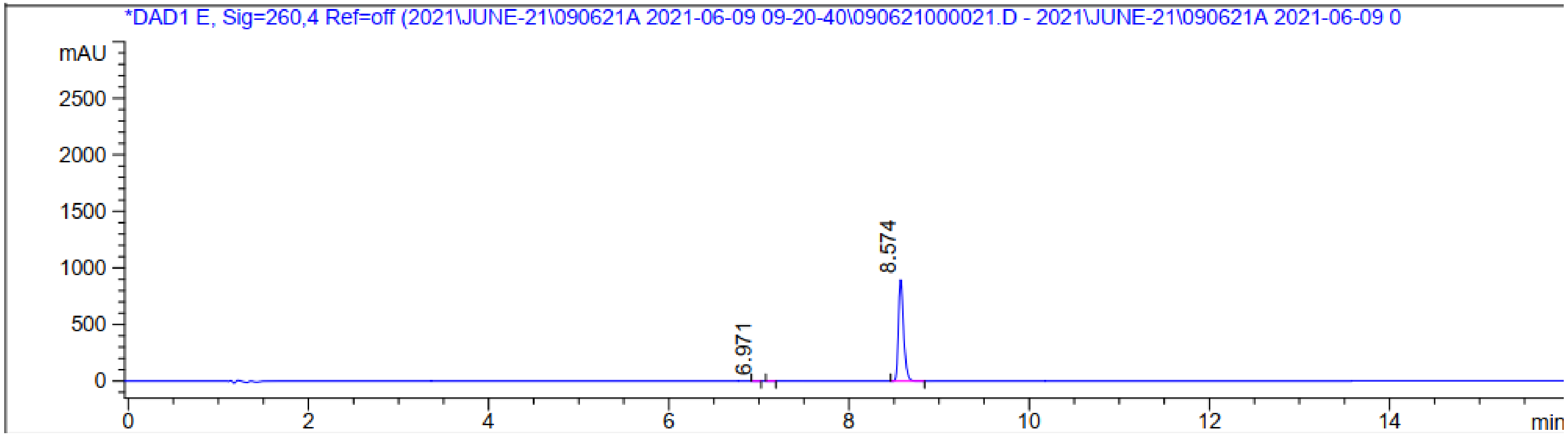

Compound 16

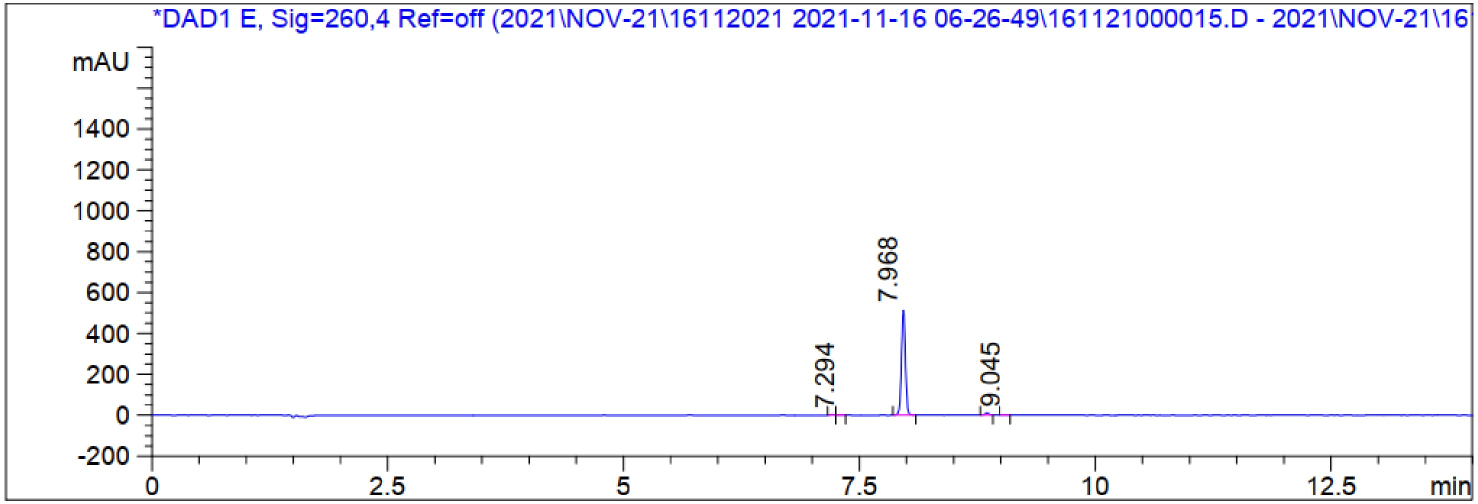

Compound 17

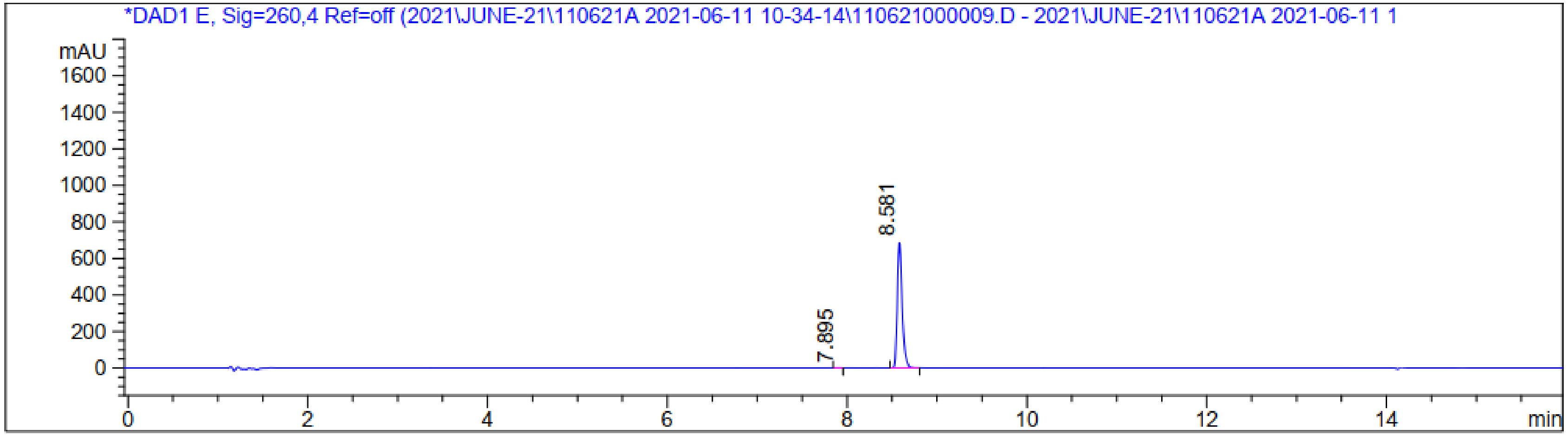

Compound 18

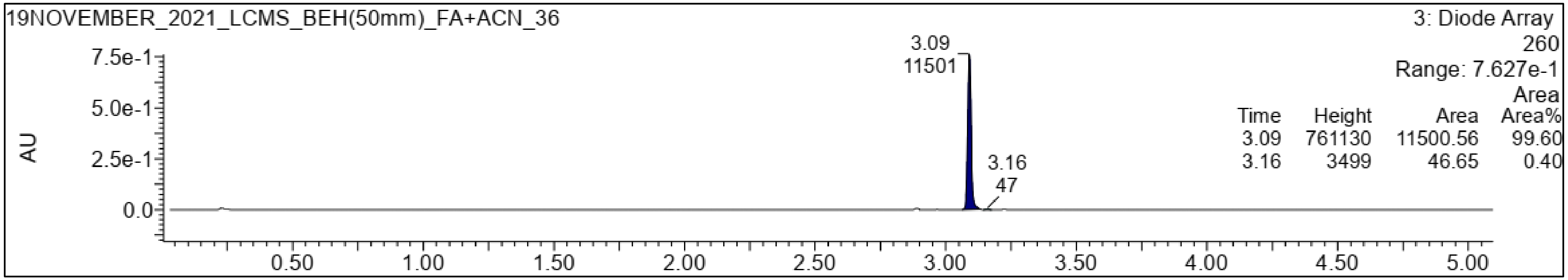

Compound 19

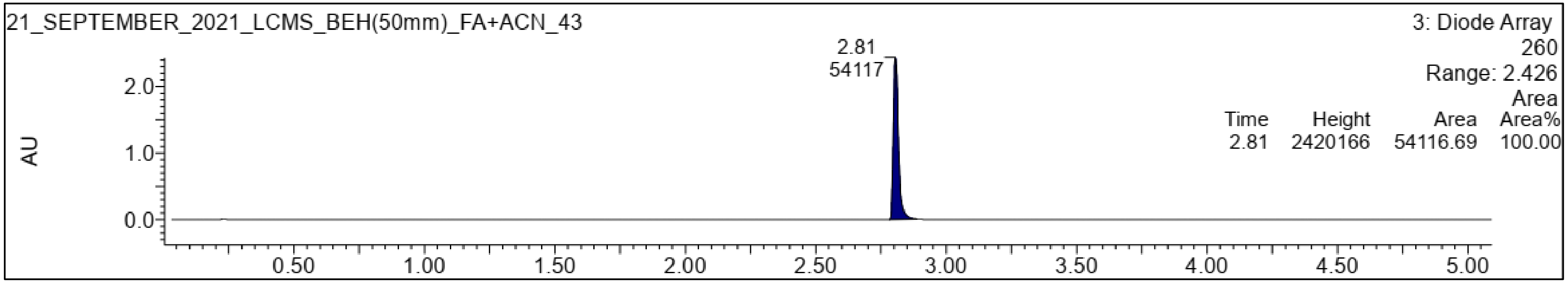

Compound 20

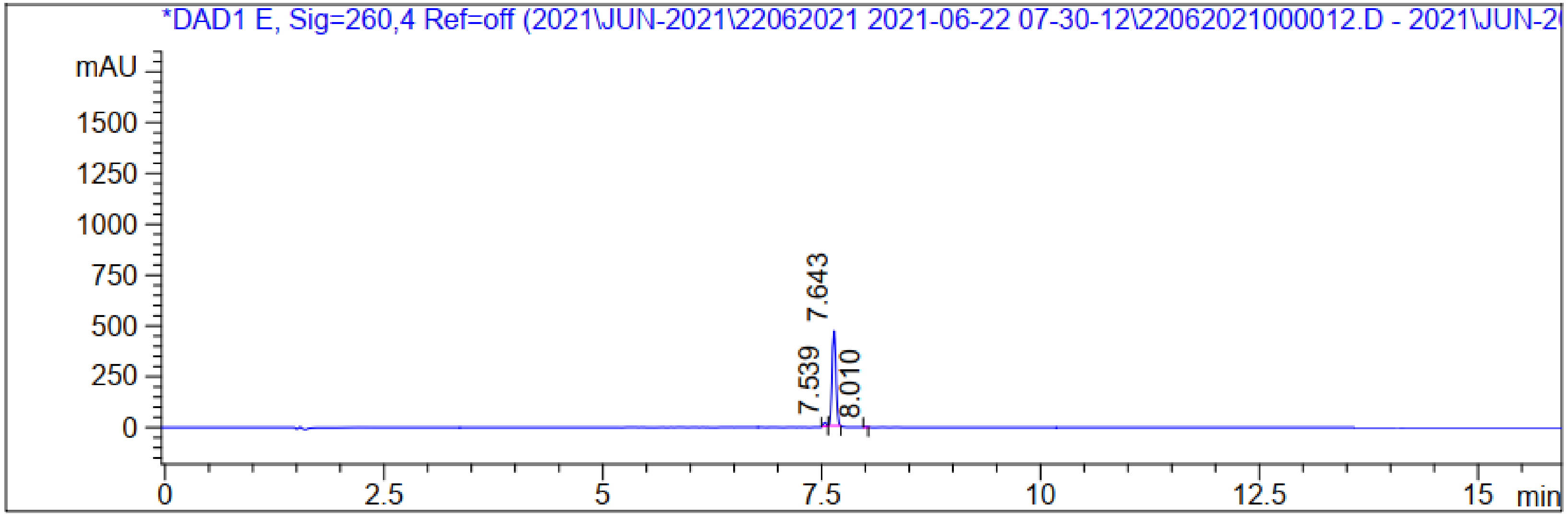

Compound 21

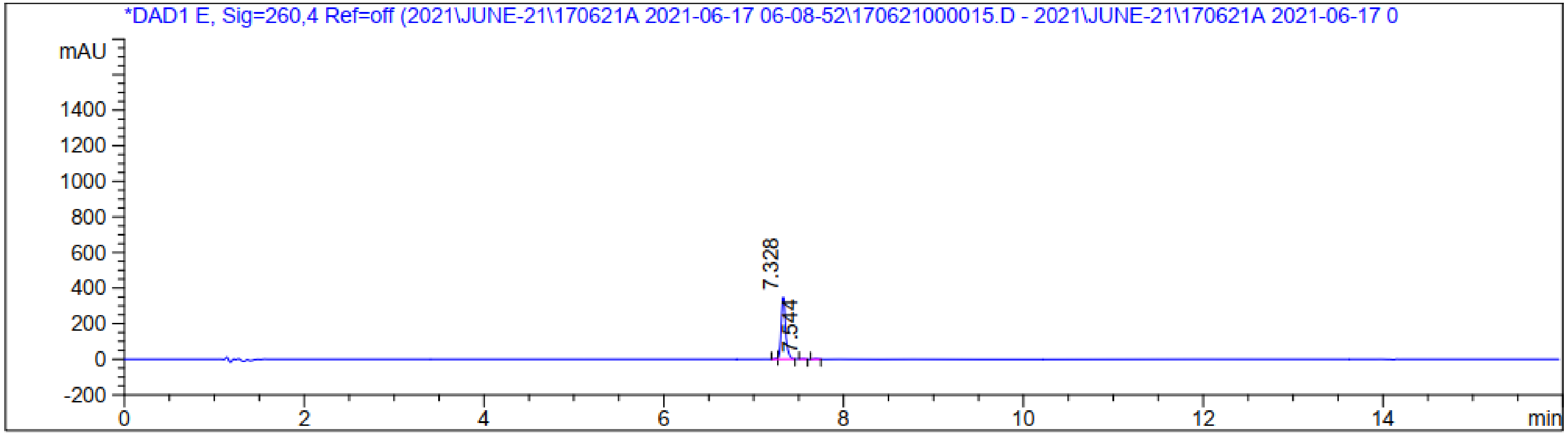

Compound 22

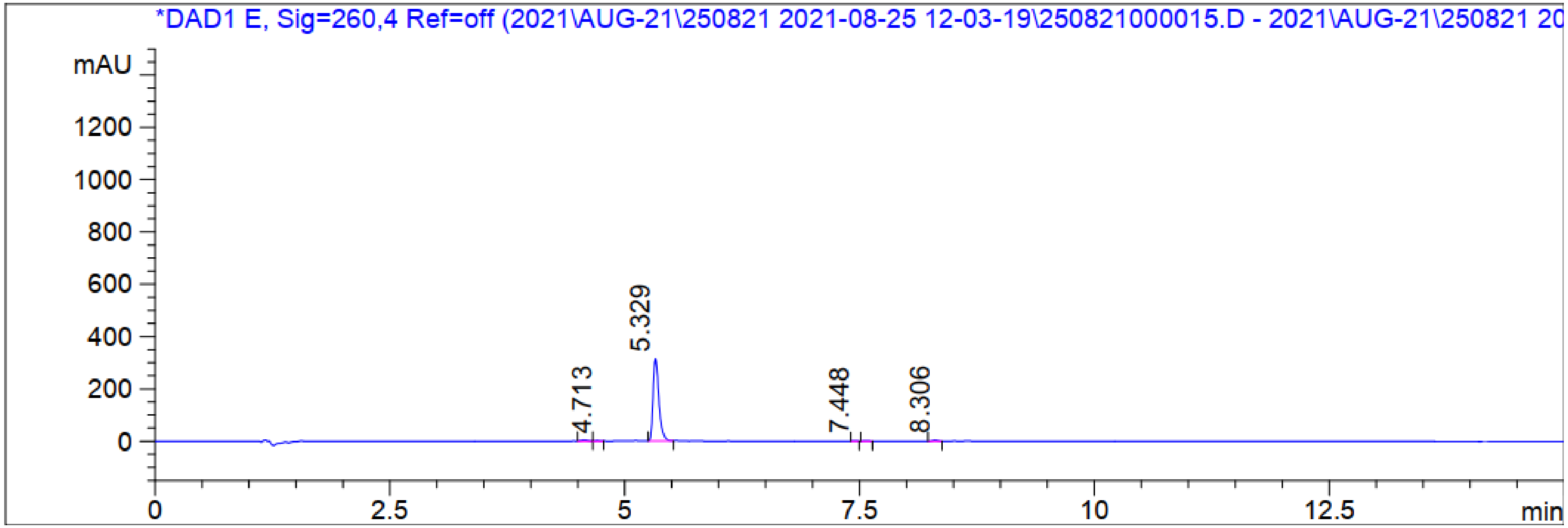

Compound 23

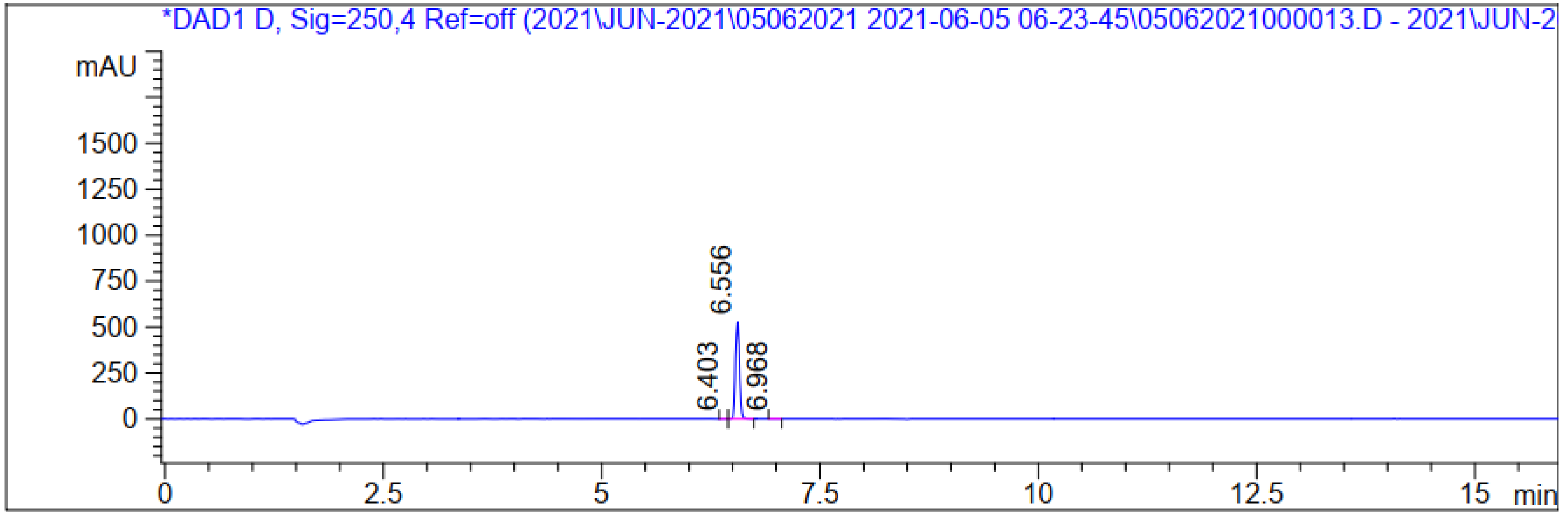

Compound 24

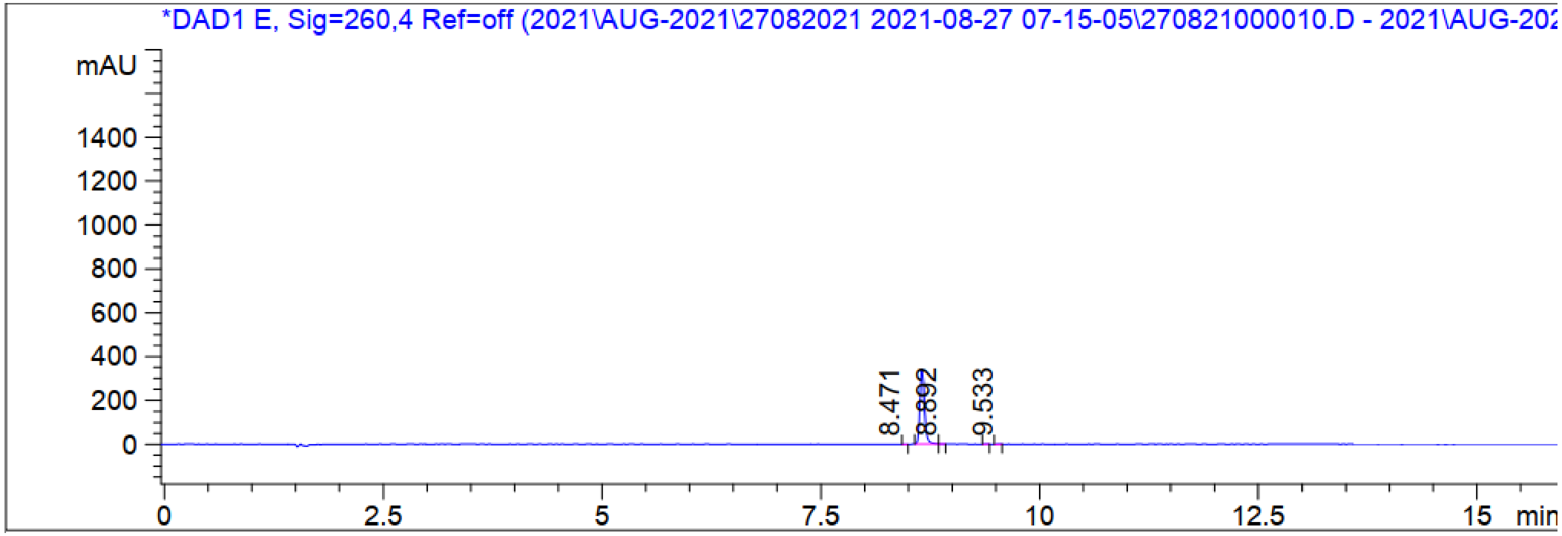

Compound 25

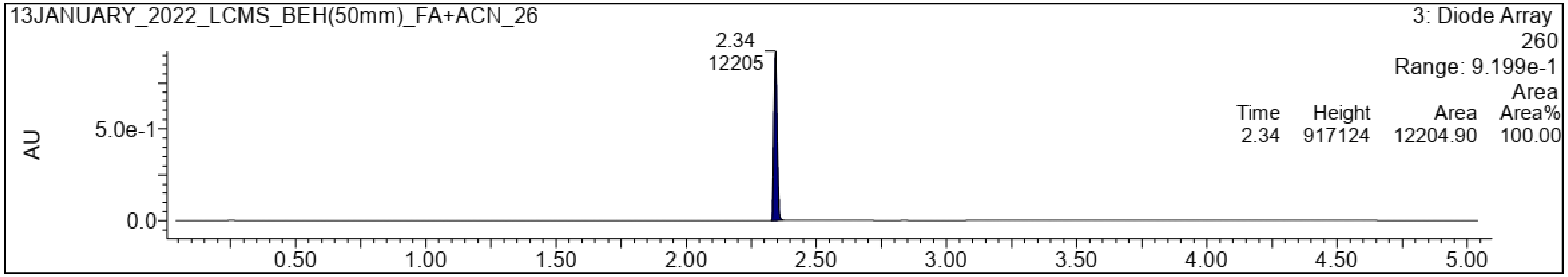

Compound 26

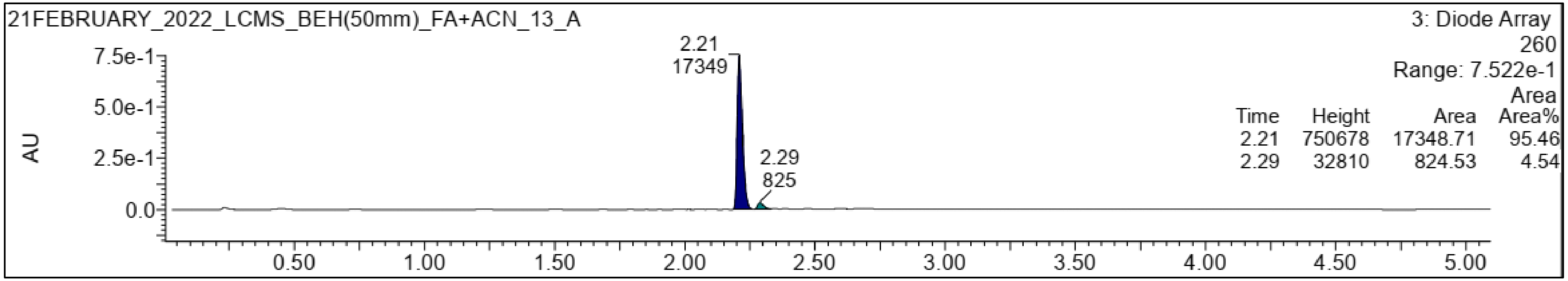

Compound 27

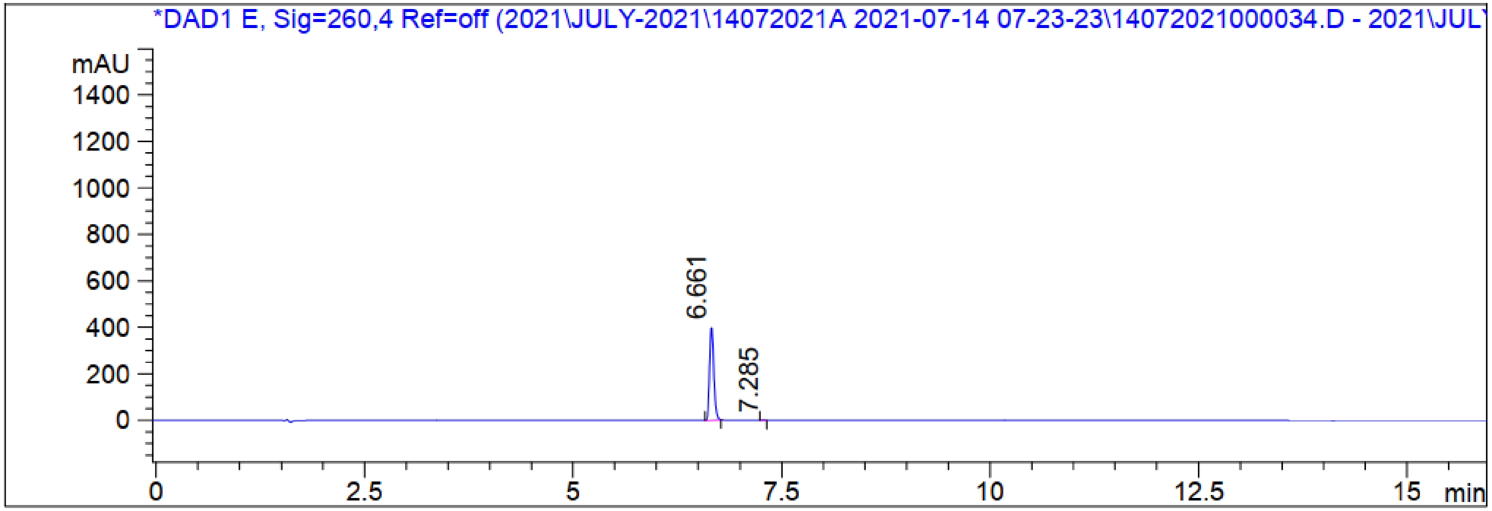

Compound 28

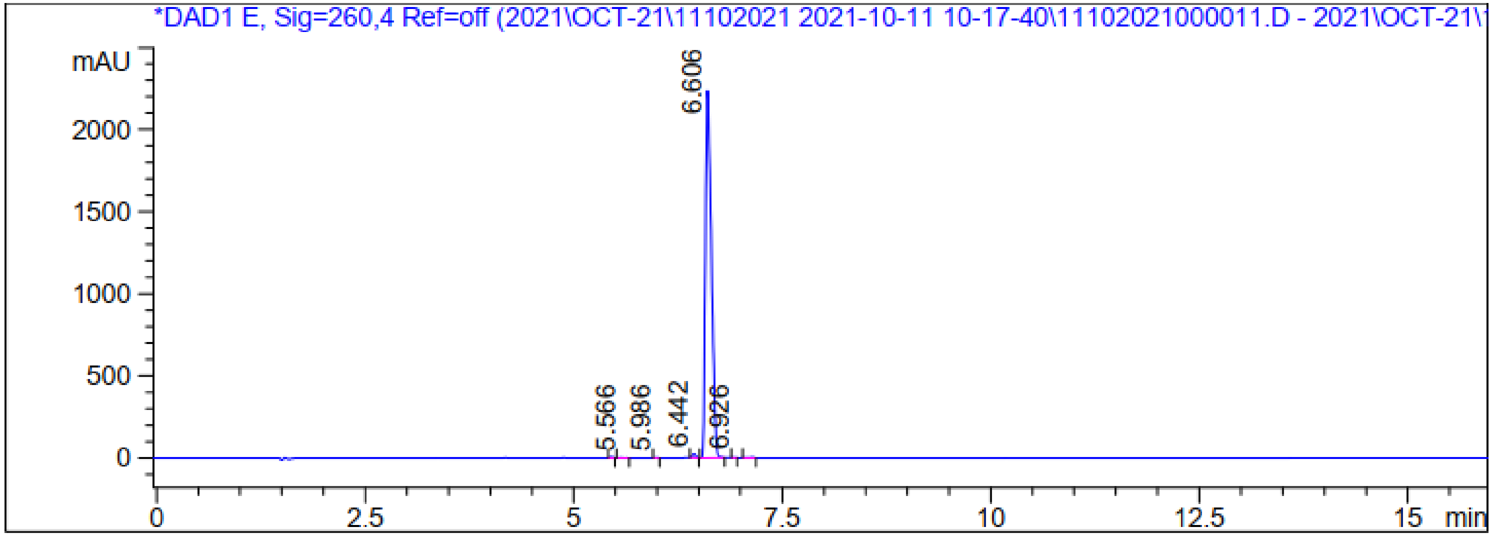

Compound 29

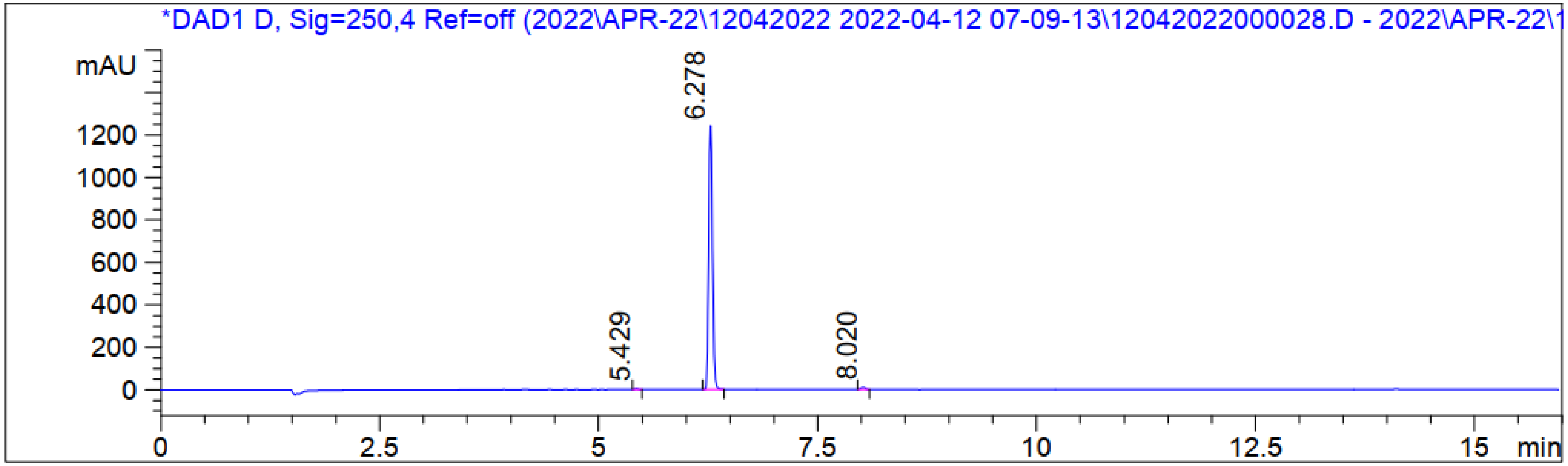

Compound 30

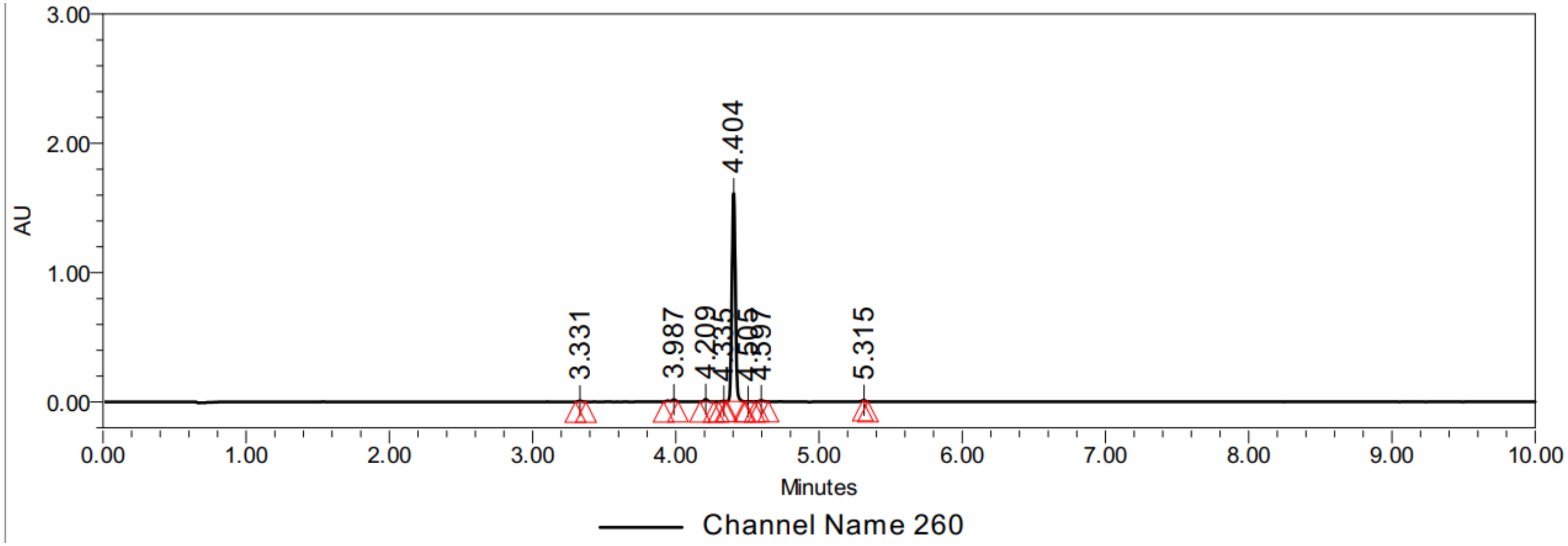

Compound 31

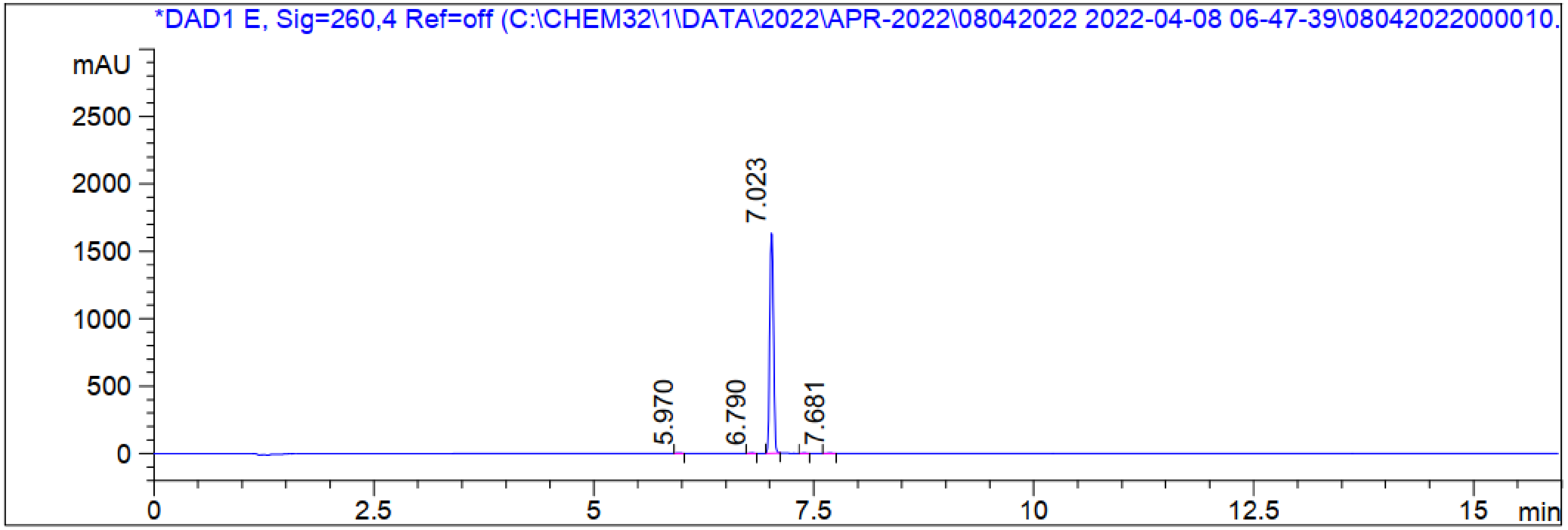

Compound 32

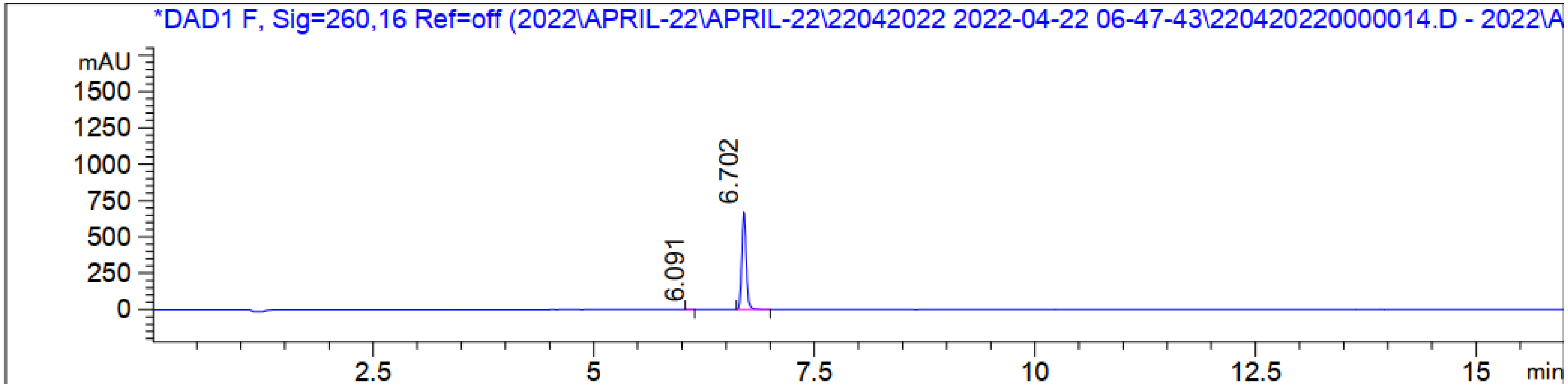

Compound 33

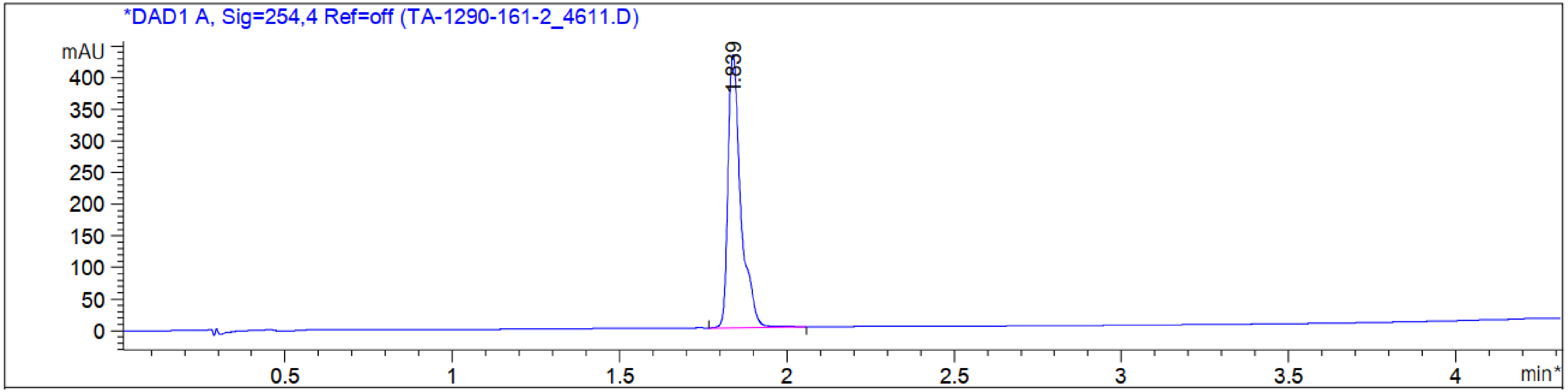

Compound 34

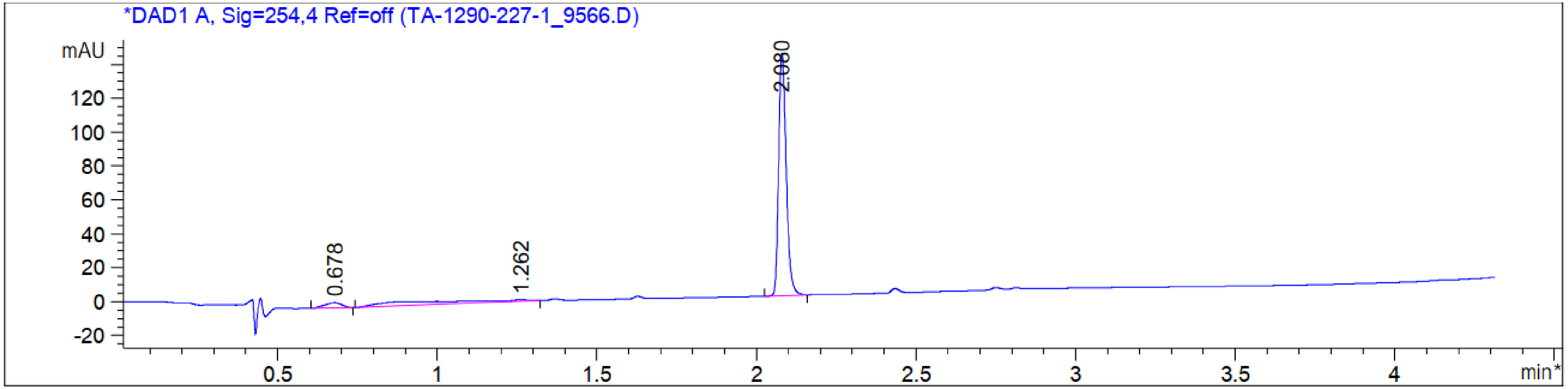

Compound 35

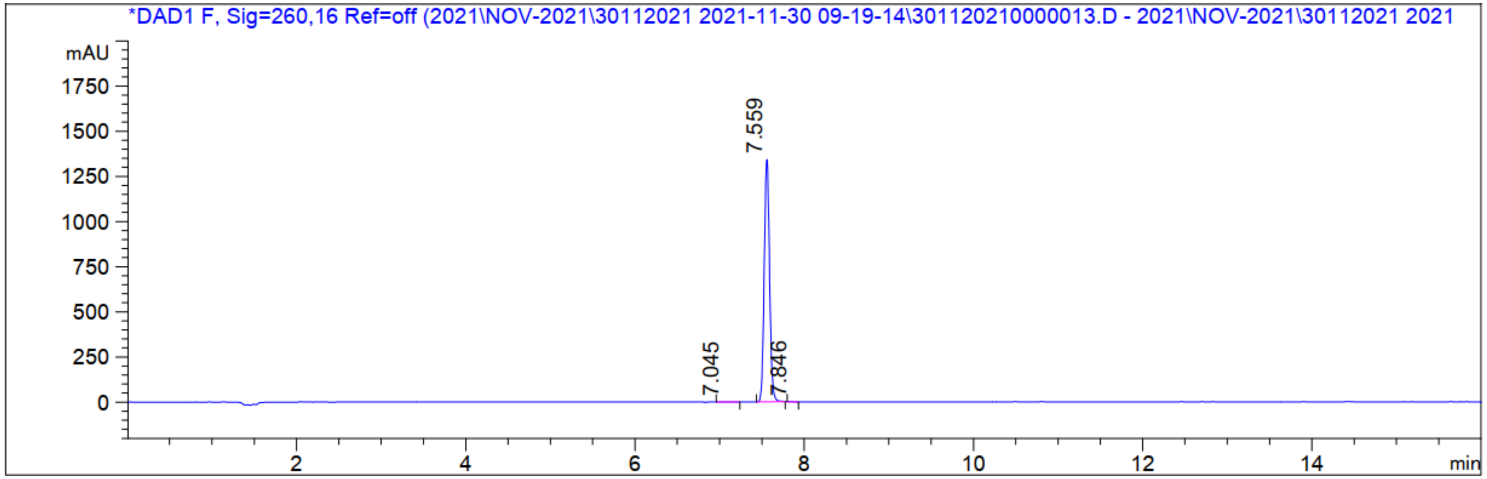

Compound 36

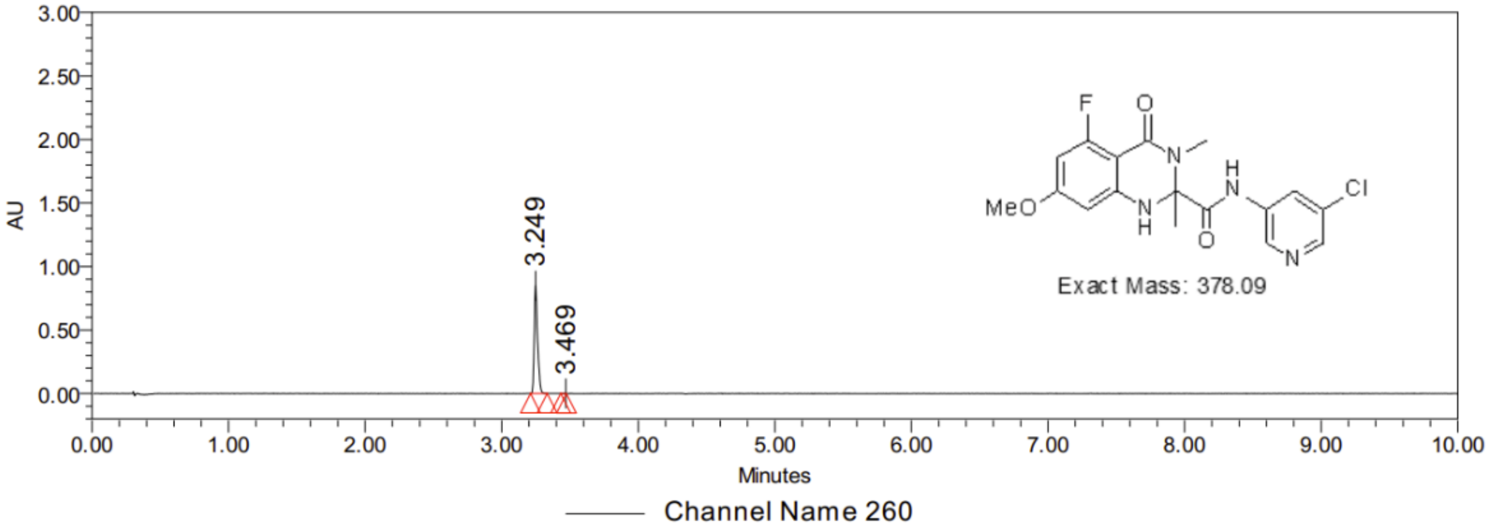

^{1}H-NMR of final compounds.

Compound 11

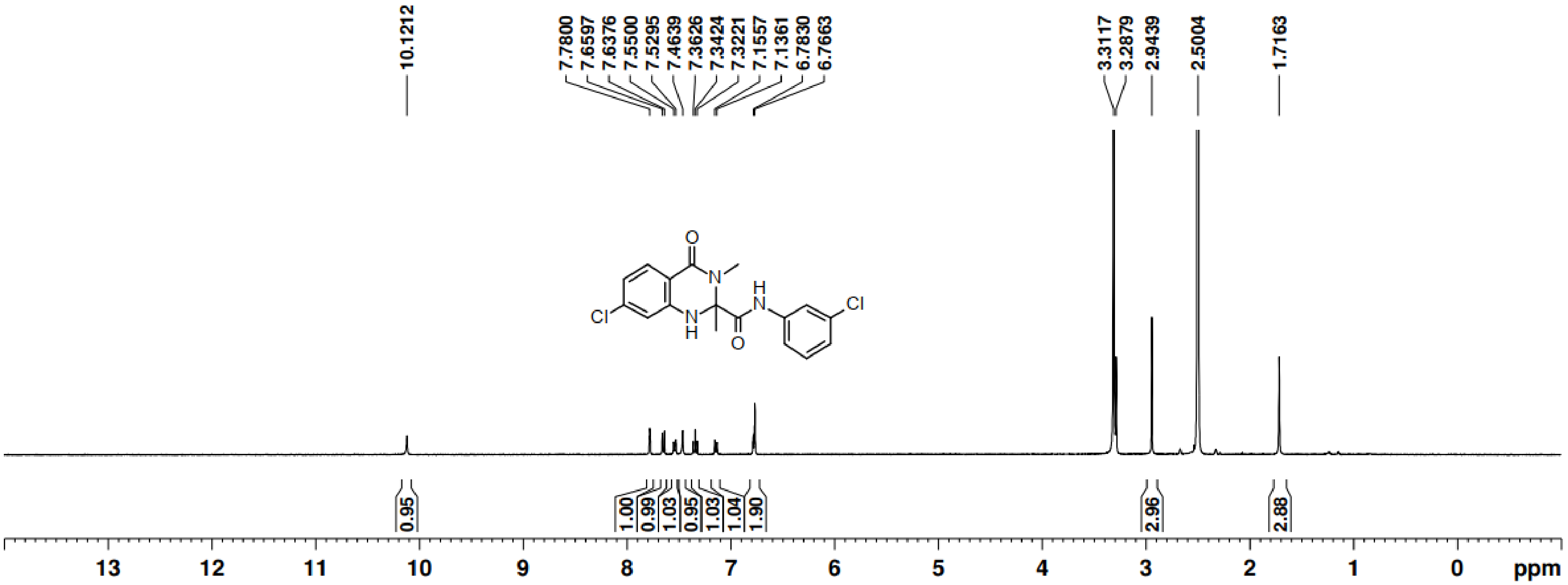

Compound 12

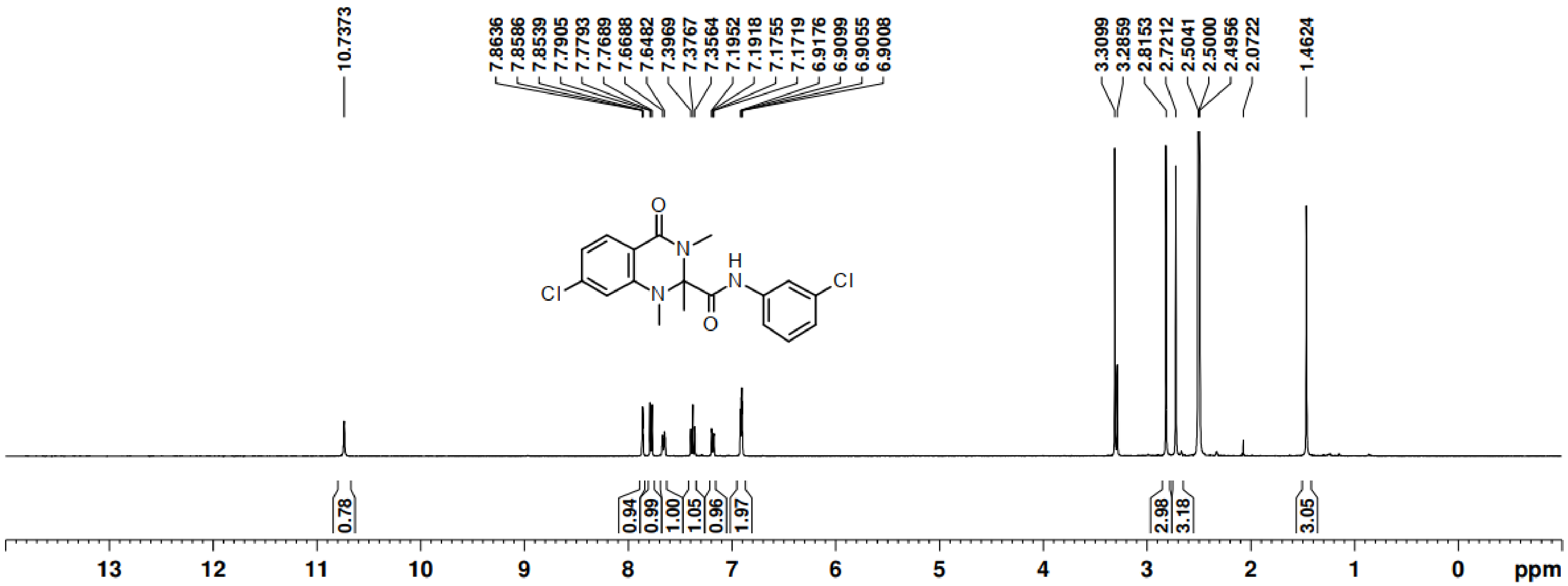

10.7373
7.8636
7.8586
7.8539
7.7905
7.7793
7.7689
7.6688
7.6482
7.3969
7.3767
7.3564
7.1952
7.1918
7.1755
7.1719
6.9176
6.9099
6.9055
6.9008
3.3099
3.2859
2.8153
2.7212
2.5041
2.5000
2.4956
2.0722
1.4624
0.78
0.94
0.99
1.00
1.05
0.96
1.97
2.98
3.18
3.05
13
12
11
10
9
8
7
6
5
4
3
2
1
0
ppm
O
N
H
N
Cl
Cl
N
O

Compound 13

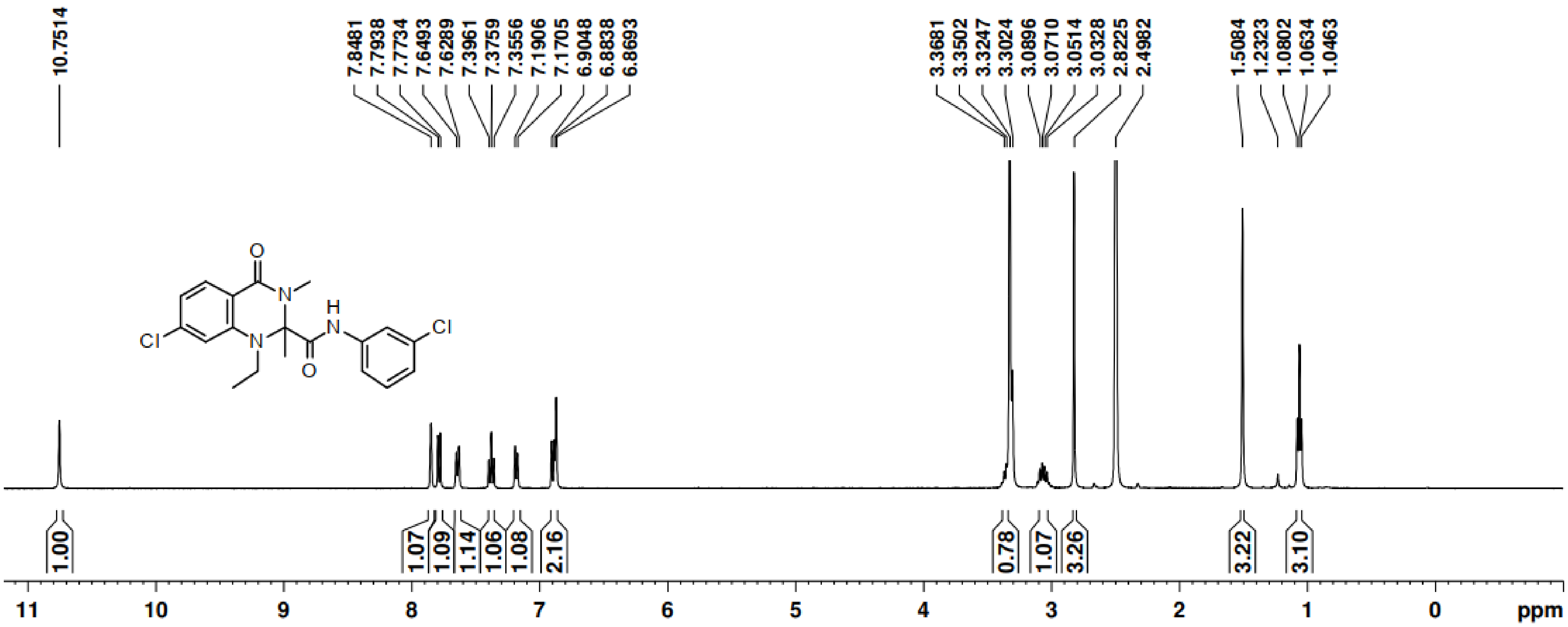
10.7514
7.8481
7.7938
7.7734
7.6493
7.6289
7.3961
7.3759
7.3556
7.1906
7.1705
6.9048
6.8838
6.8693
3.3681
3.3502
3.3247
3.3024
3.0896
3.0710
3.0514
3.0328
2.8225
2.4982
1.5084
1.2323
1.0802
1.0634
1.0463
O
N
H
N
Cl
Cl
N
O
1.00
1.07
1.09
1.14
1.06
1.08
2.16
0.78
1.07
3.26
3.22
3.10
11
10
9
8
7
6
5
4
3
2
1
0
ppm

Compound 14

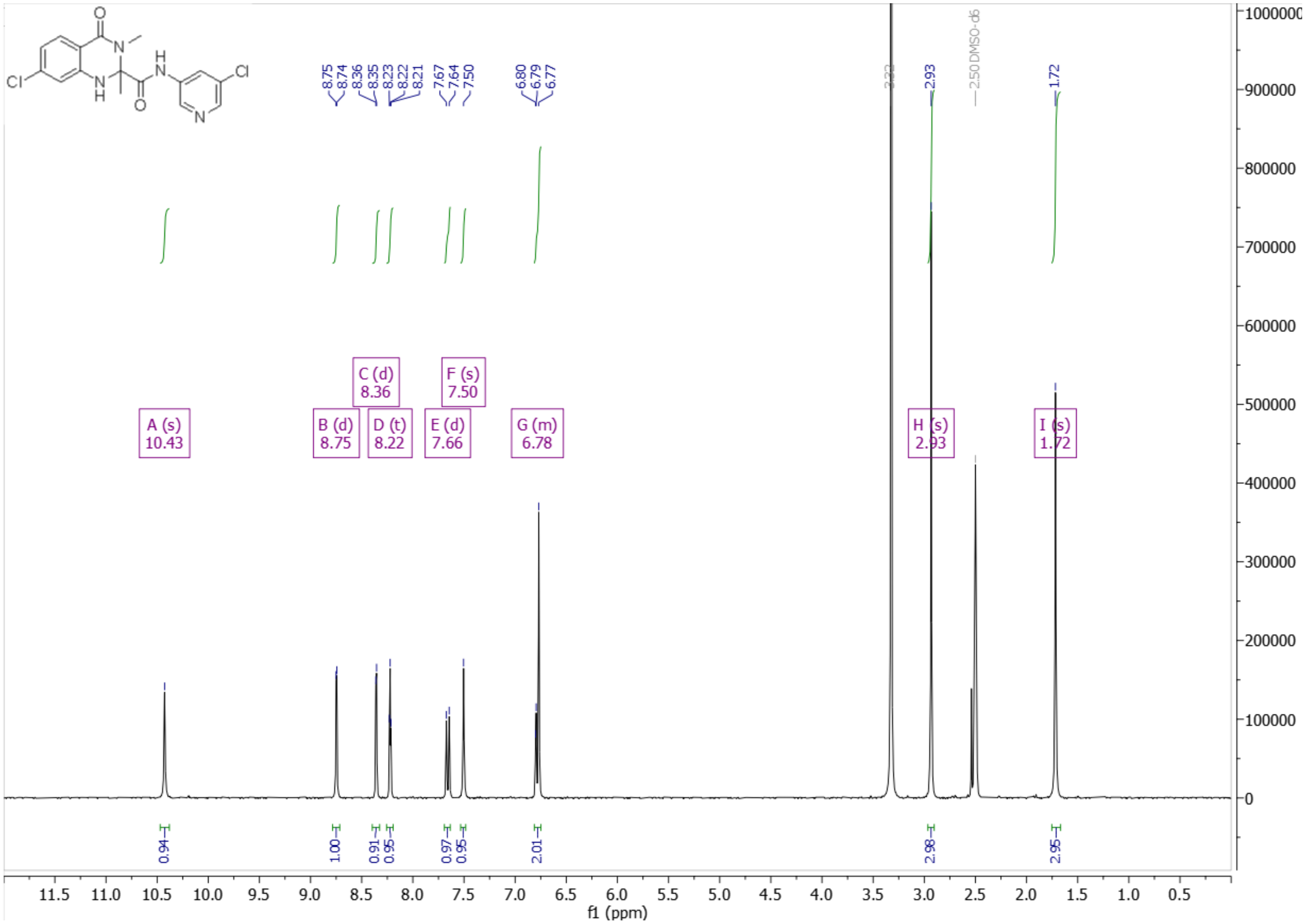

8.75
8.74
8.36
8.35
8.23
8.22
8.21
7.67
7.64
7.50
6.80
6.79
6.77
2.93
2.50 DMSO-d6
1.72
A (s) 10.43
B (d) 8.75
C (d) 8.36
D (t) 8.22
E (d) 7.66
F (s) 7.50
G (m) 6.78
H (s) 2.93
I (s) 1.72
0.94
1.00
0.91
0.95
0.97
0.95
2.01
2.98
2.95
f1 (ppm)

Compound 15

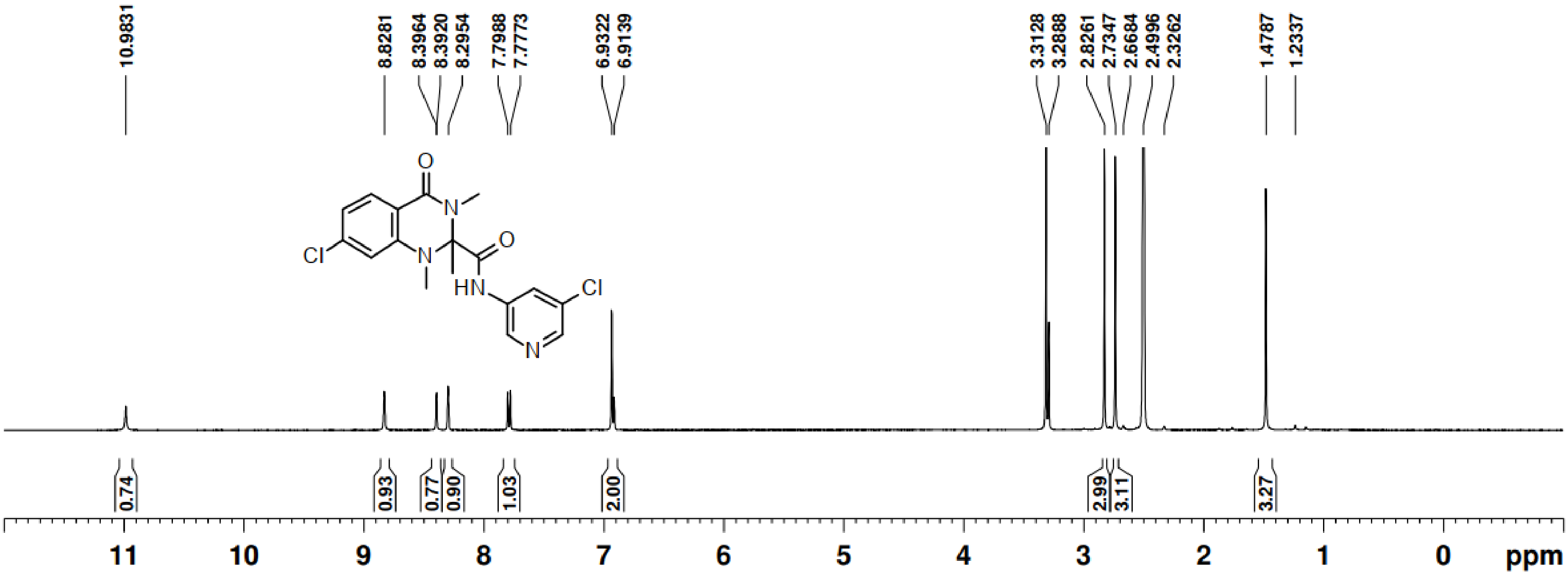

10.9831
8.8281
8.3964
8.3920
8.2954
7.7988
7.7773
6.9322
6.9139
3.3128
3.2888
2.8261
2.7347
2.6684
2.4996
2.3262
1.4787
1.2337
0.74
0.93
0.77
0.90
1.03
2.00
2.99
3.11
3.27
11
10
9
8
7
6
5
4
3
2
1
0
ppm

Compound 16

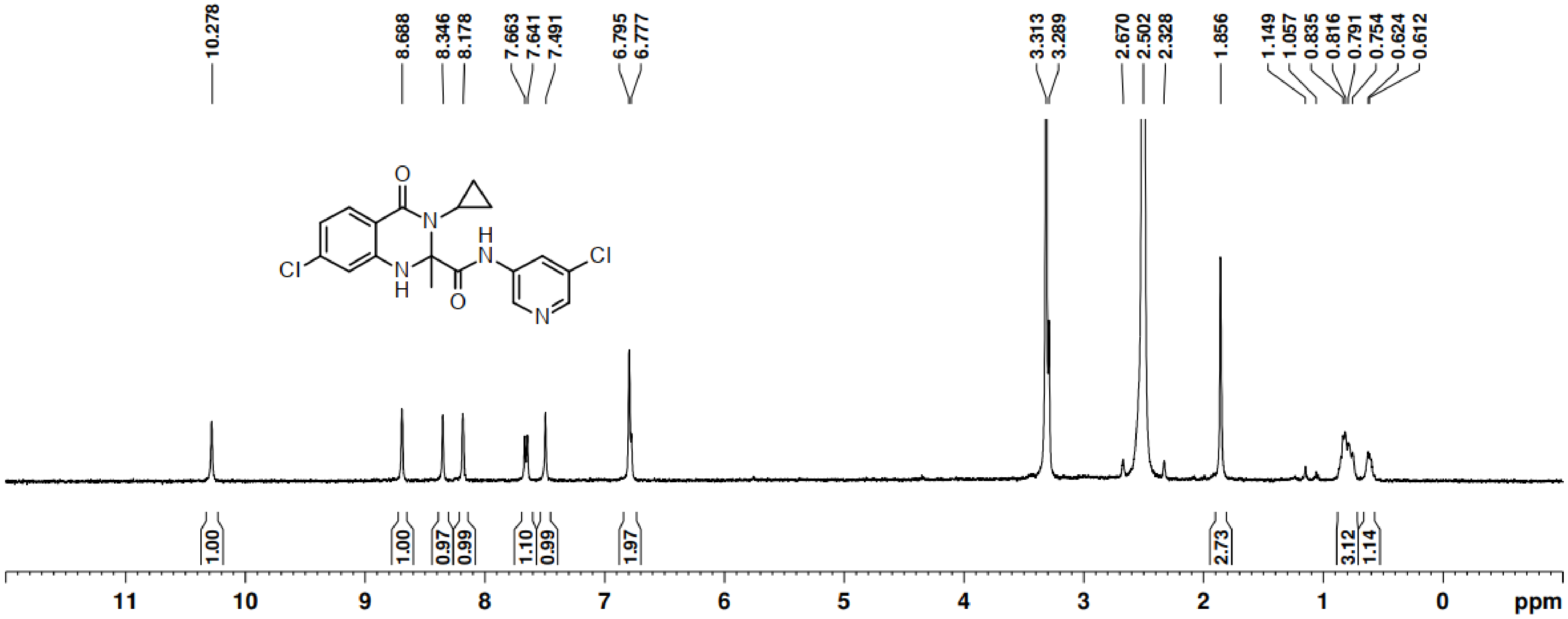
10.278
8.688
8.346
8.178
7.663
7.641
7.491
6.795
6.777
3.313
3.289
2.670
2.502
2.328
1.856
1.149
1.057
0.835
0.816
0.791
0.754
0.624
0.612
O
N
Cl
N
H
H
N
O
Cl
N
1.00
1.00
0.97
0.99
1.10
0.99
1.97
2.73
3.12
1.14
11
10
9
8
7
6
5
4
3
2
1
0
ppm

Compound 17

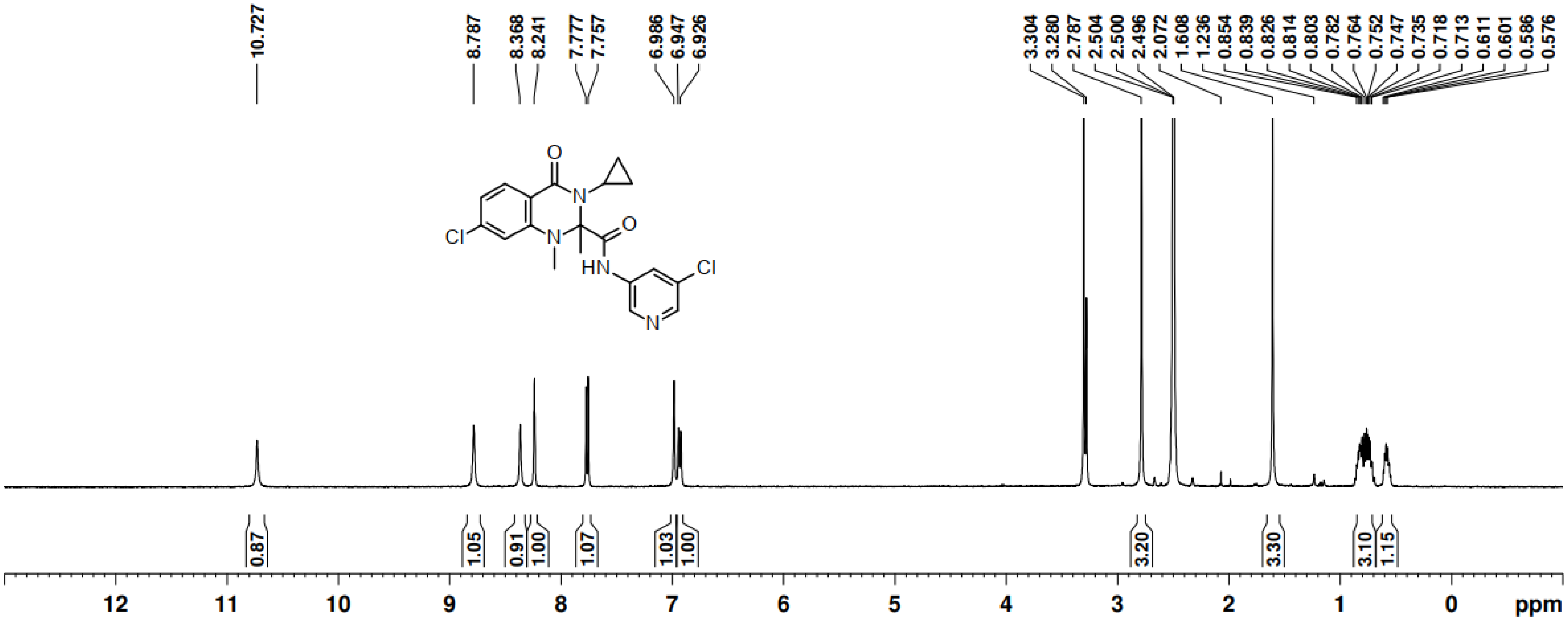
10.727
8.787
8.368
8.241
7.777
7.757
6.986
6.947
6.926
3.304
3.280
2.787
2.504
2.500
2.496
2.072
1.608
1.236
0.854
0.839
0.826
0.814
0.803
0.782
0.764
0.752
0.747
0.735
0.718
0.713
0.611
0.601
0.586
0.576
O
N
Cl
N
O
HN
Cl
N
0.87
1.05
0.91
1.00
1.07
1.03
1.00
3.20
3.30
3.10
1.15
12
11
10
9
8
7
6
5
4
3
2
1
0
ppm

Compound 18

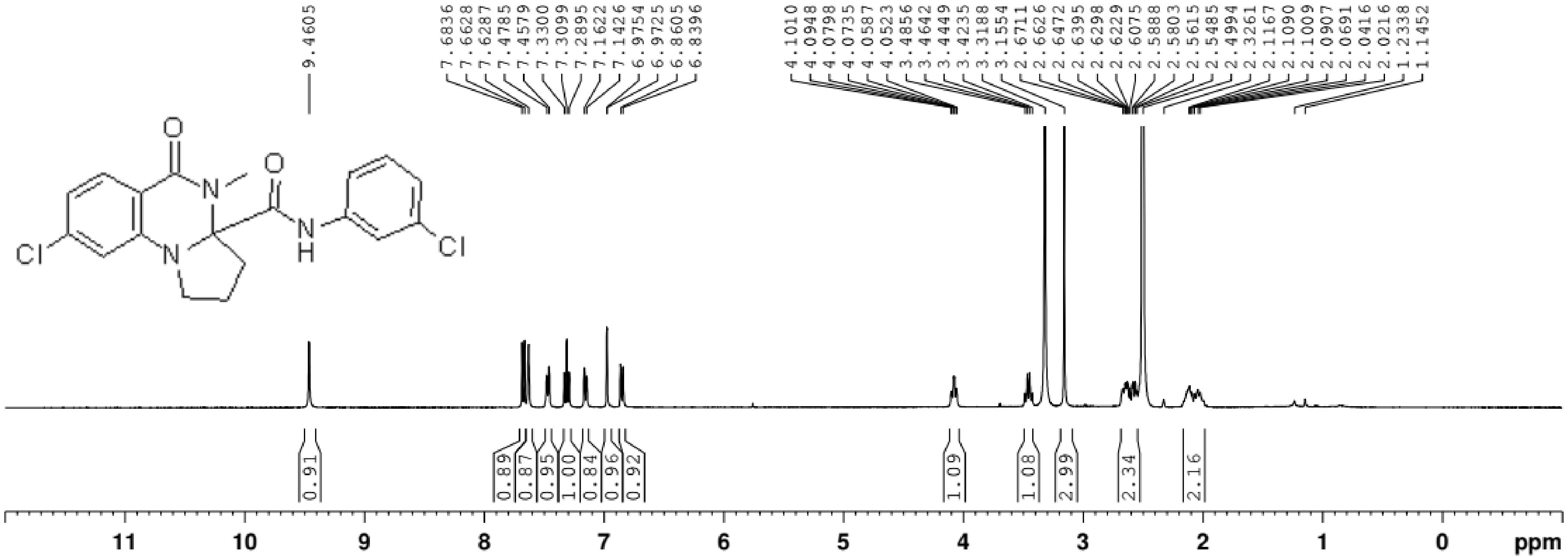

Compound 19

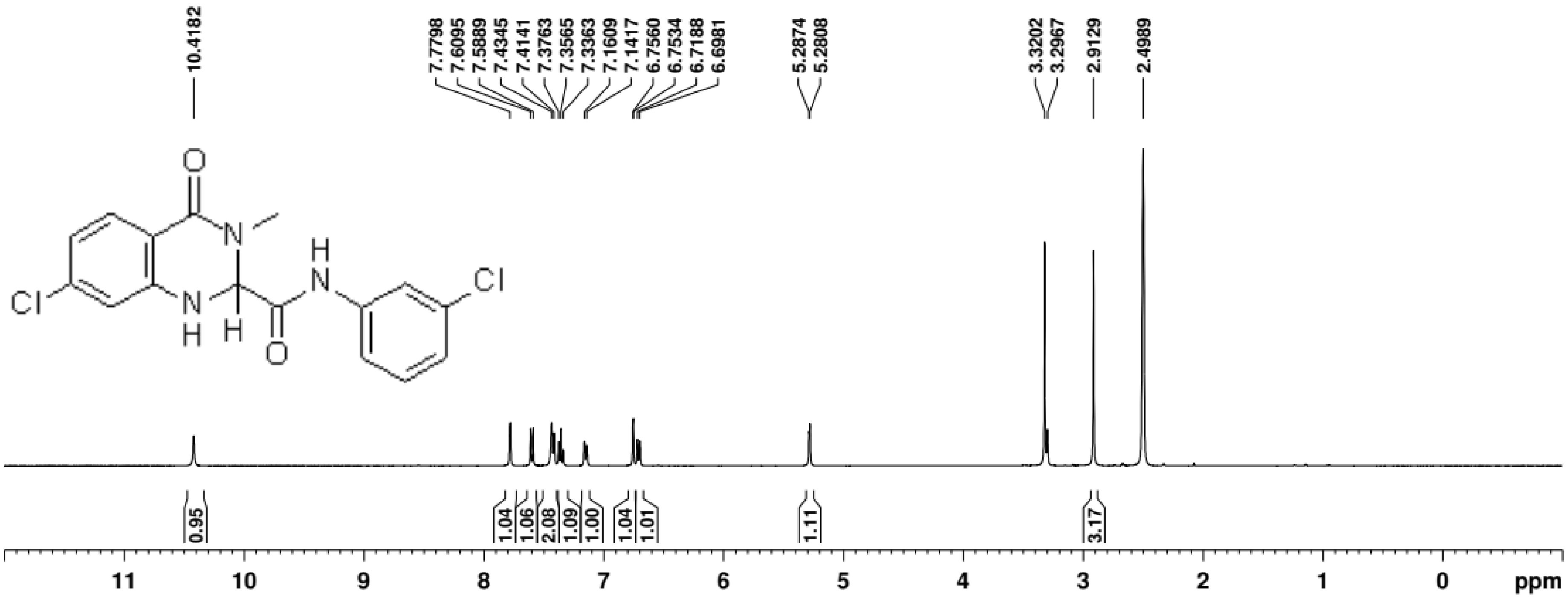

10.4182
7.7798
7.6095
7.5889
7.4345
7.4141
7.3763
7.3565
7.3363
7.1609
7.1417
6.7560
6.7534
6.7188
6.6981
5.2874
5.2808
3.3202
3.2967
2.9129
2.4989
O
N
H
N
Cl
Cl
N
H
H
O
0.95
1.04
1.06
2.08
1.09
1.00
1.04
1.01
1.11
3.17
11
10
9
8
7
6
5
4
3
2
1
0
ppm

Compound 20

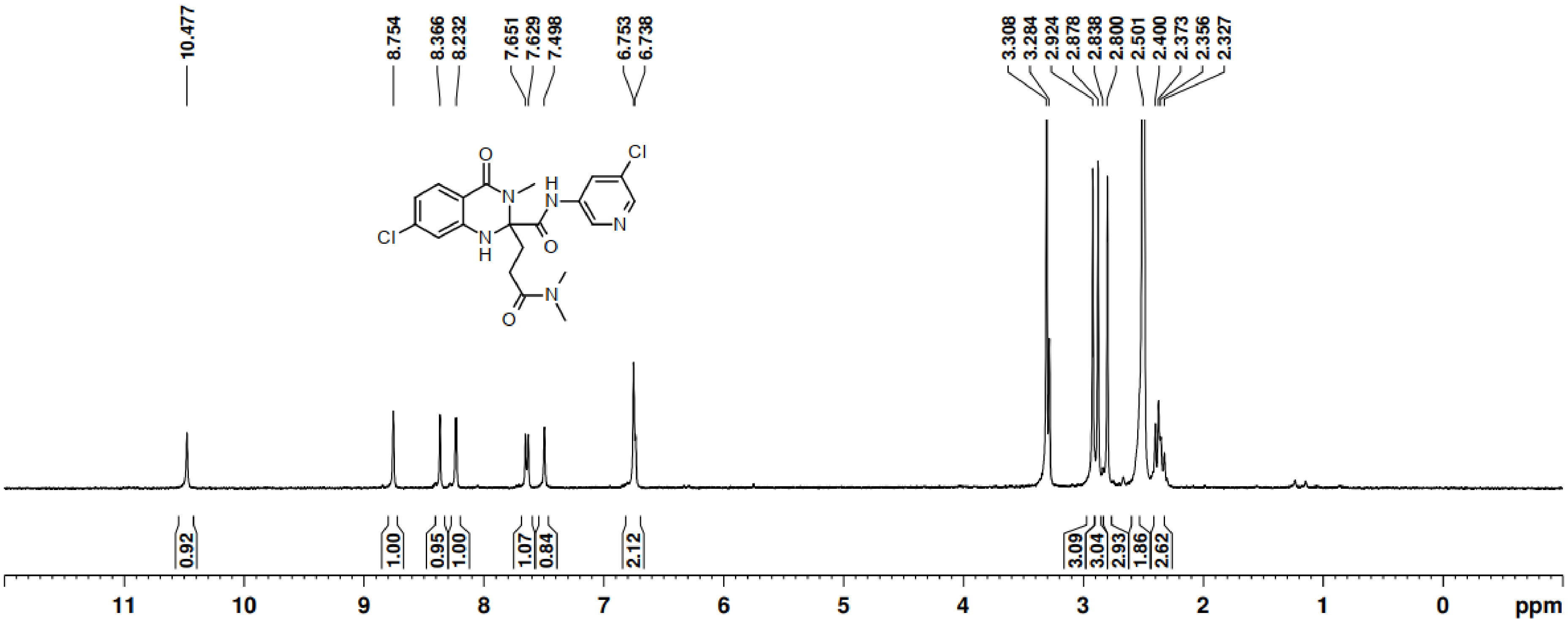
10.477
8.754
8.366
8.232
7.651
7.629
7.498
6.753
6.738
3.308
3.284
2.924
2.878
2.838
2.800
2.501
2.400
2.373
2.356
2.327
0.92
1.00
0.95
1.00
1.07
0.84
2.12
3.09
3.04
2.93
1.86
2.62
11 10 9 8 7 6 5 4 3 2 1 0 ppm

Compound 21

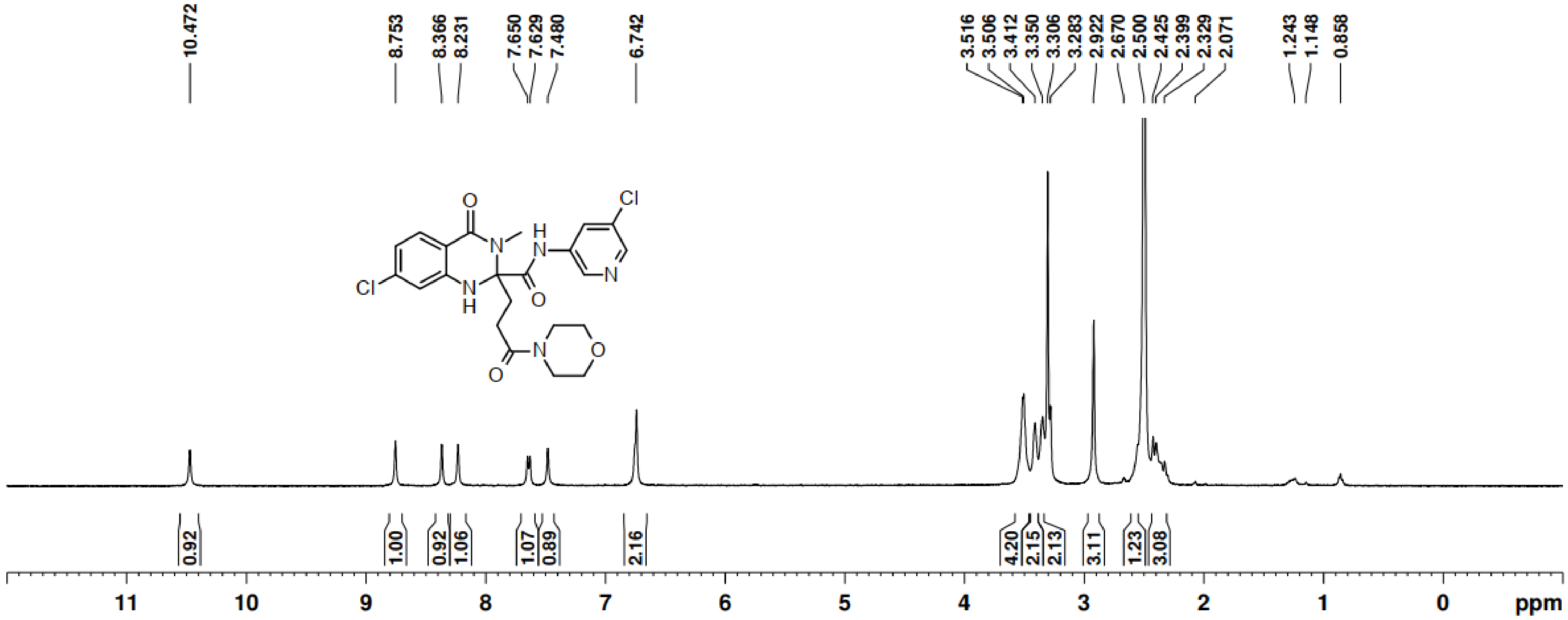

10.472
8.753
8.366
8.231
7.650
7.629
7.480
6.742
3.516
3.506
3.412
3.350
3.306
3.283
2.922
2.670
2.500
2.425
2.399
2.329
2.071
1.243
1.148
0.858
0.92
1.00
0.92
1.06
1.07
0.89
2.16
4.20
2.15
2.13
3.11
1.23
3.08
11
10
9
8
7
6
5
4
3
2
1
0
ppm

Compound 22

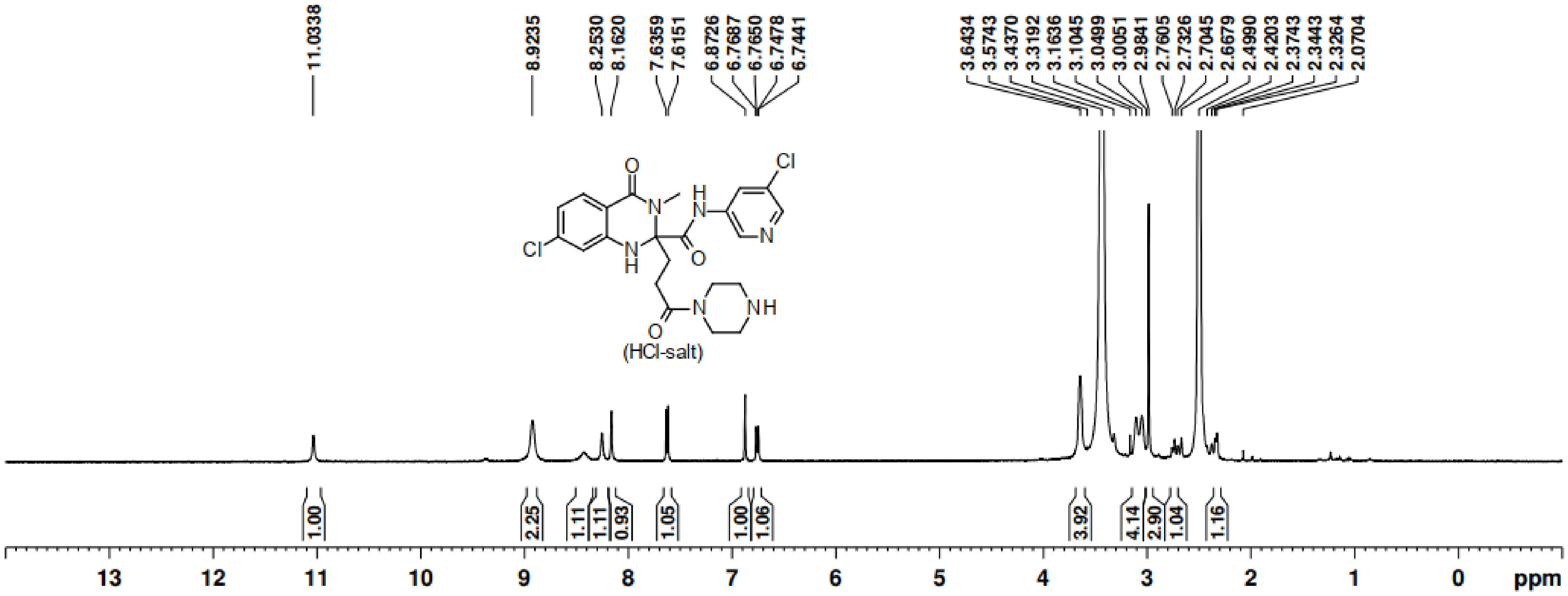

Compound 23

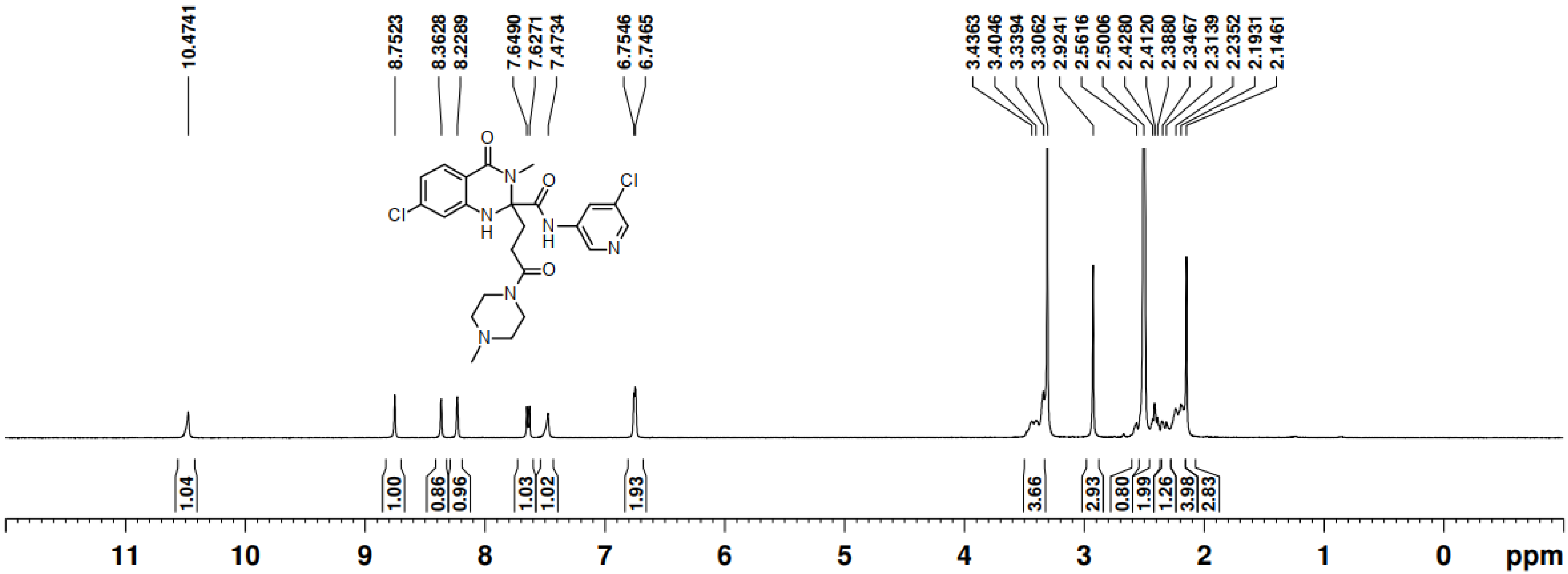
10.4741
8.7523
8.3628
8.2289
7.6490
7.6271
7.4734
6.7546
6.7465
3.4363
3.4046
3.3394
3.3062
2.9241
2.5616
2.5006
2.4280
2.4120
2.3880
2.3467
2.3139
2.2352
2.1931
2.1461
O
N
Cl
N
H
O
Cl
N
H
N
O
N
N
1.04
1.00
0.86
0.96
1.03
1.02
1.93
3.66
2.93
0.80
1.99
1.26
3.98
2.83
11
10
9
8
7
6
5
4
3
2
1
0
ppm

Compound 24

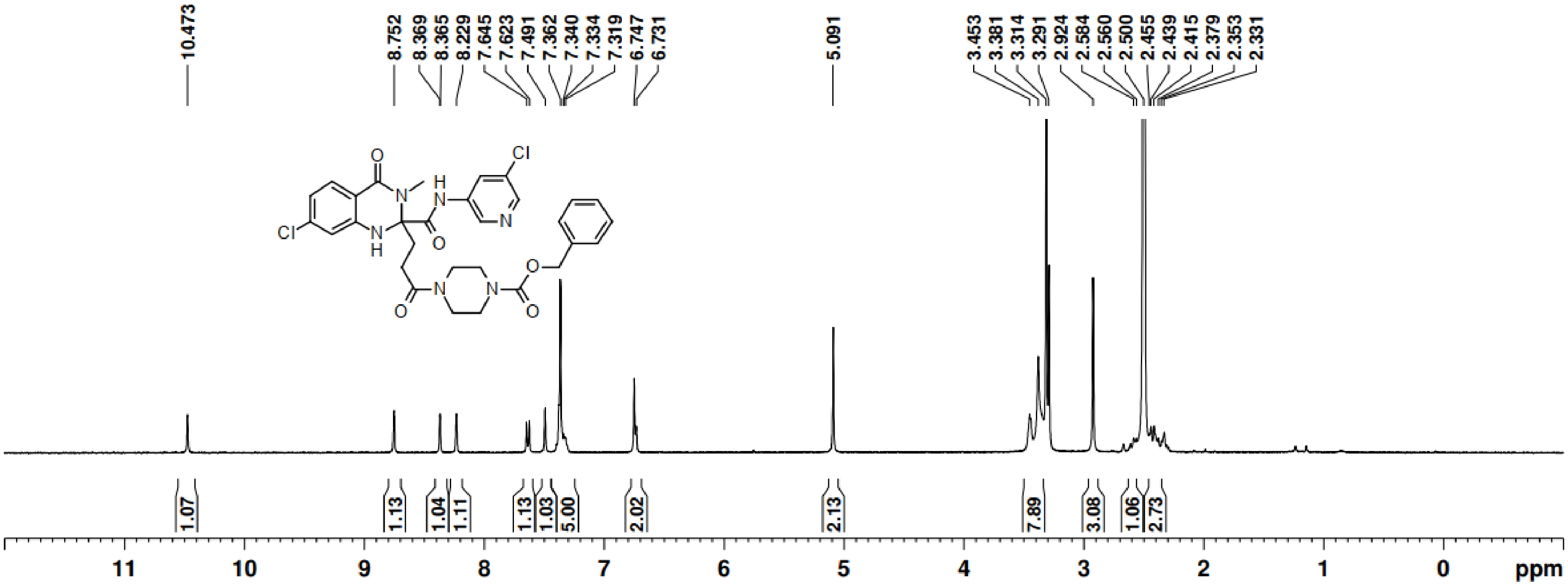
10.473
8.752
8.369
8.365
8.229
7.645
7.623
7.491
7.362
7.340
7.334
7.319
6.747
6.731
5.091
3.453
3.381
3.314
3.291
2.924
2.584
2.560
2.500
2.455
2.439
2.415
2.379
2.353
2.331
1.07
1.13
1.04
1.11
1.13
1.03
5.00
2.02
2.13
7.89
3.08
1.06
2.73
11
10
9
8
7
6
5
4
3
2
1
0
ppm

Compound 25

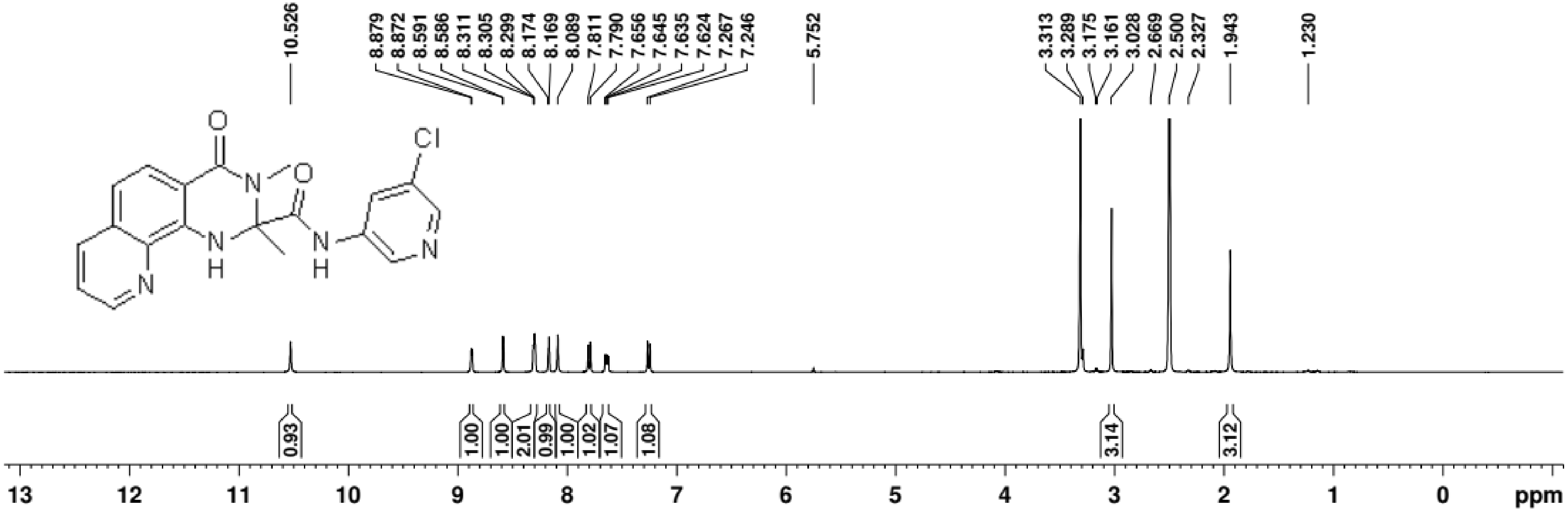
10.526
8.879
8.872
8.591
8.586
8.311
8.305
8.299
8.174
8.169
8.089
7.811
7.790
7.656
7.645
7.635
7.624
7.267
7.246
5.752
3.313
3.289
3.175
3.161
3.028
2.669
2.500
2.327
1.943
1.230
0.93
1.00
1.00
2.01
0.99
1.00
1.02
1.07
1.08
3.14
3.12
13
12
11
10
9
8
7
6
5
4
3
2
1
0
ppm

Compound 26

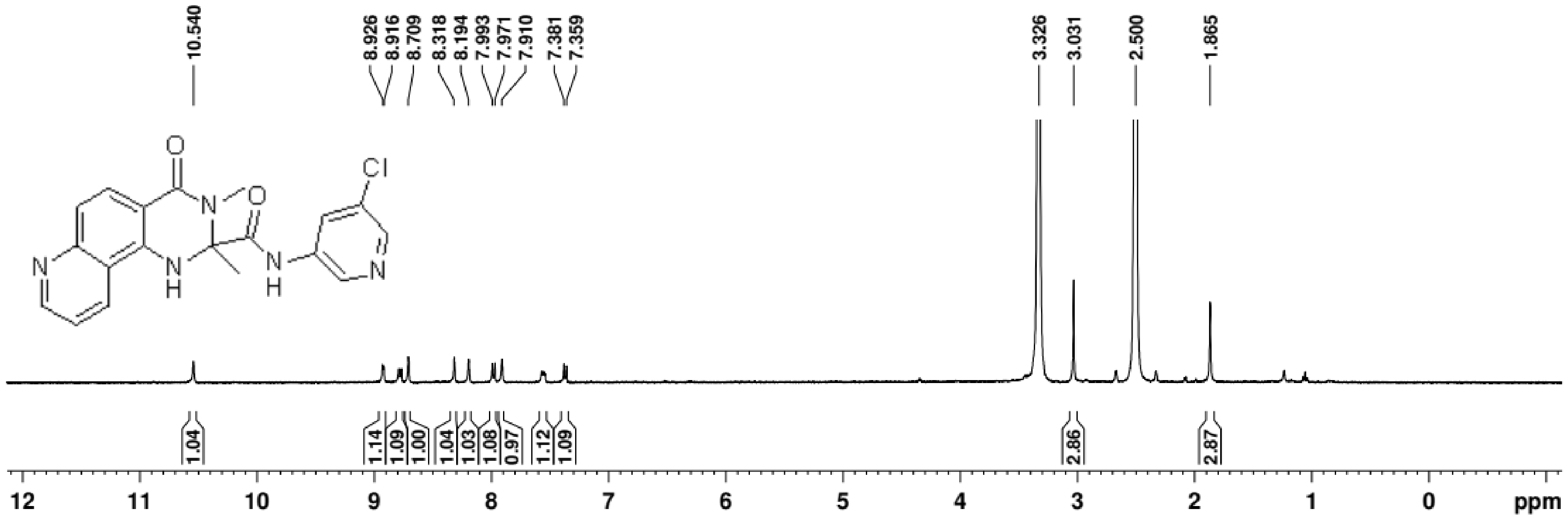
10.540
8.926
8.916
8.709
8.318
8.194
7.993
7.971
7.910
7.381
7.359
3.326
3.031
2.500
1.865
1.04
1.14
1.09
1.00
1.04
1.03
1.08
0.97
1.12
1.09
2.86
2.87
12
11
10
9
8
7
6
5
4
3
2
1
0
ppm

Compound 27

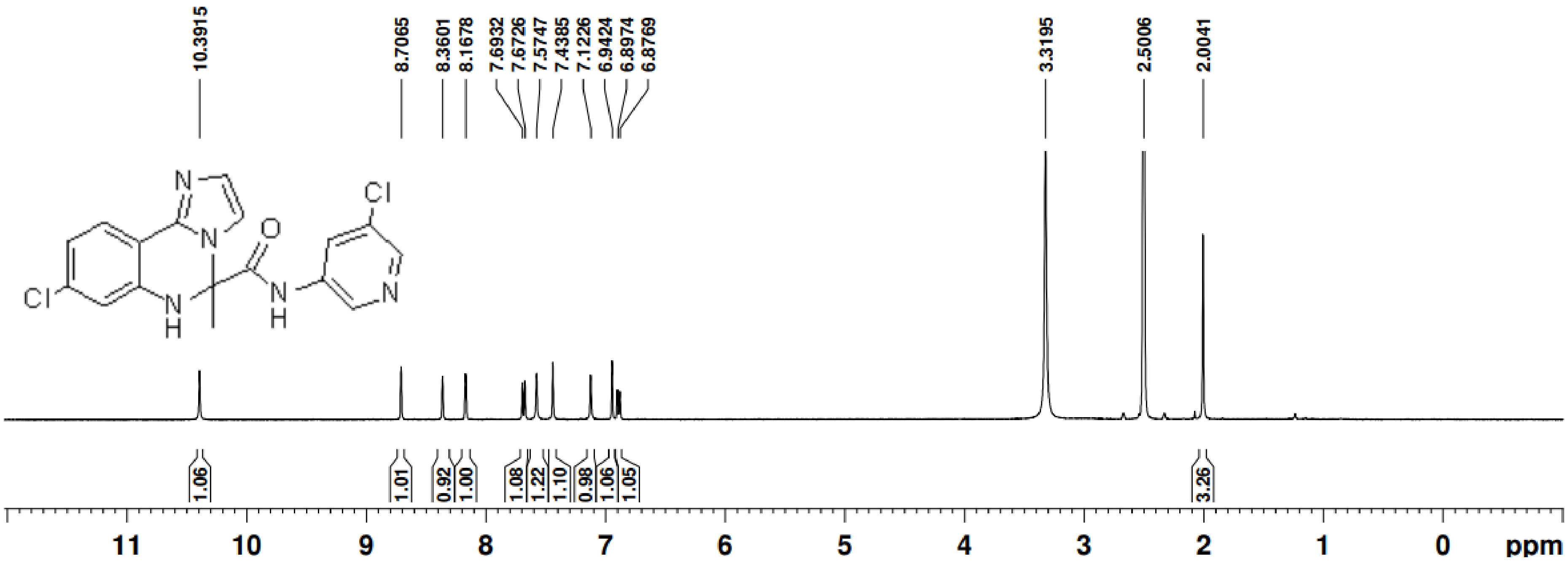
10.3915
8.7065
8.3601
8.1678
7.6932
7.6726
7.5747
7.4385
7.1226
6.9424
6.8974
6.8769
3.3195
2.5006
2.0041
1.06
1.01
0.92
1.00
1.08
1.22
1.10
0.98
1.06
1.05
3.26
11
10
9
8
7
6
5
4
3
2
1
0
ppm

Compound 28

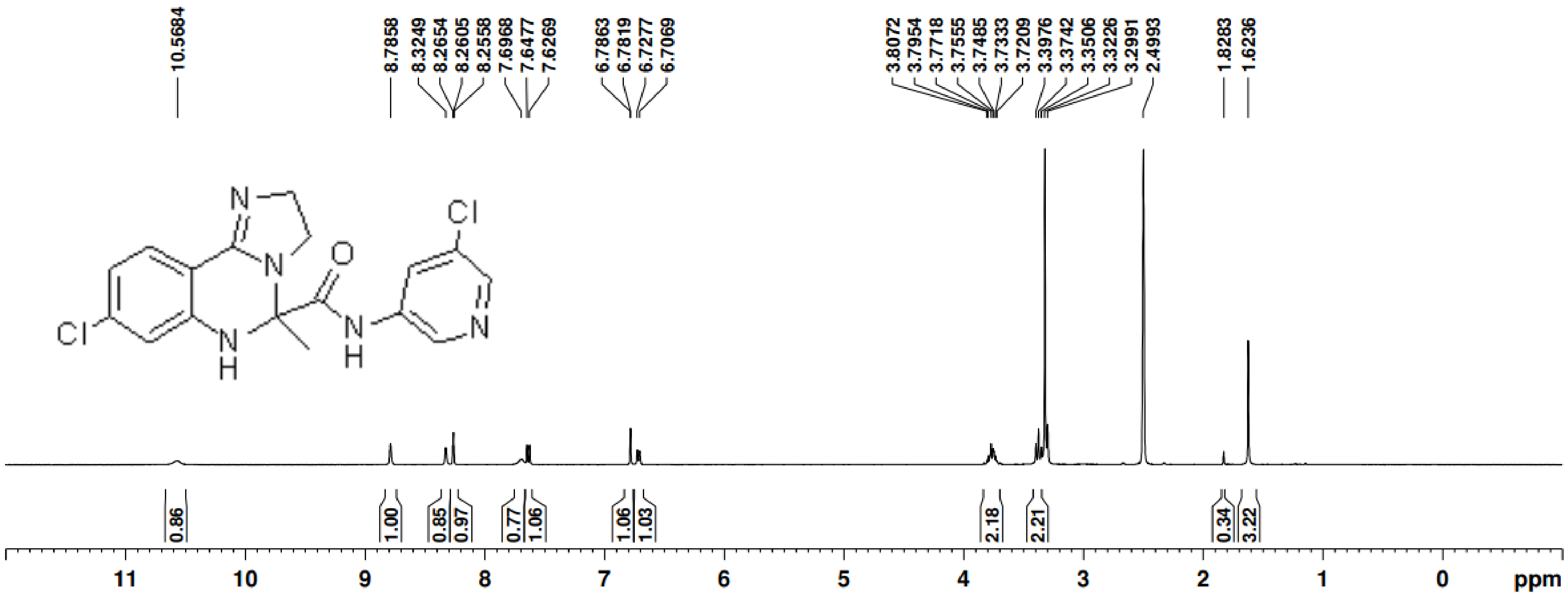

10.5684
8.7858
8.3249
8.2654
8.2605
8.2558
7.6968
7.6477
7.6269
6.7863
6.7819
6.7277
6.7069
3.8072
3.7954
3.7718
3.7555
3.7485
3.7333
3.7209
3.3976
3.3742
3.3506
3.3226
3.2991
2.4993
1.8283
1.6236
Cl
N
N
O
N
H
Cl
N
H
N
Cl
0.86
1.00
0.85
0.97
0.77
1.06
1.06
1.03
2.18
2.21
0.34
3.22
11
10
9
8
7
6
5
4
3
2
1
0
ppm

Compound 29

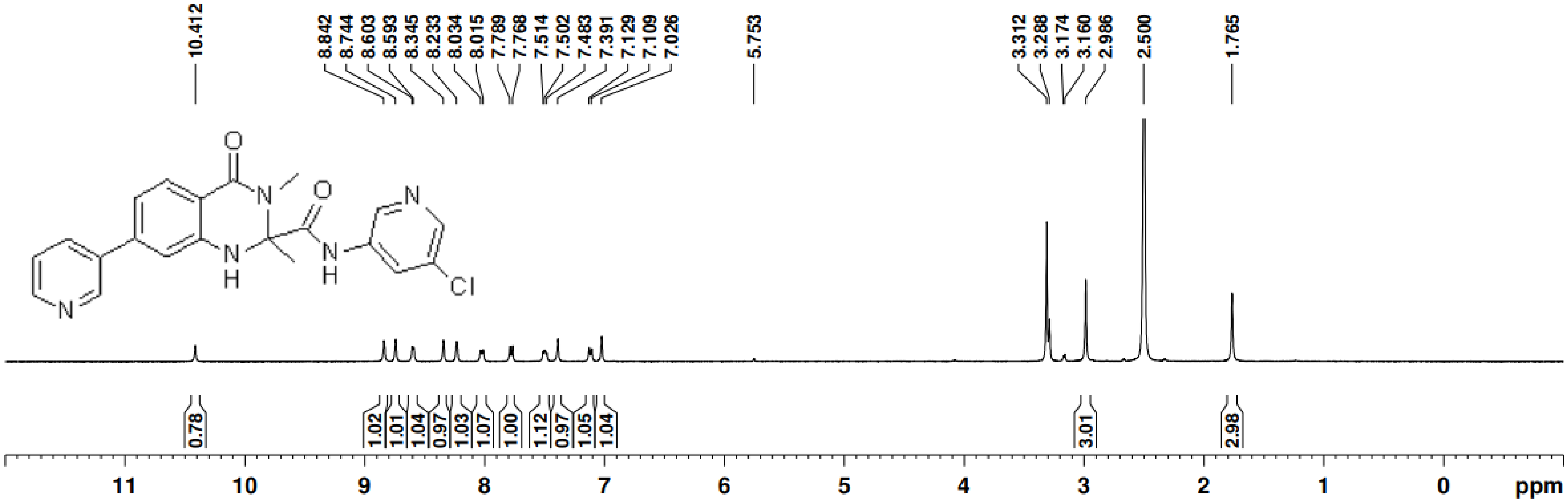
10.412
8.842
8.744
8.603
8.593
8.345
8.233
8.034
8.015
7.789
7.768
7.514
7.502
7.483
7.391
7.129
7.109
7.026
5.753
3.312
3.288
3.174
3.160
2.986
2.500
1.765
0.78
1.02
1.01
1.04
0.97
1.03
1.07
1.00
1.12
0.97
1.05
1.04
3.01
2.98
11
10
9
8
7
6
5
4
3
2
1
0
ppm

Compound 30

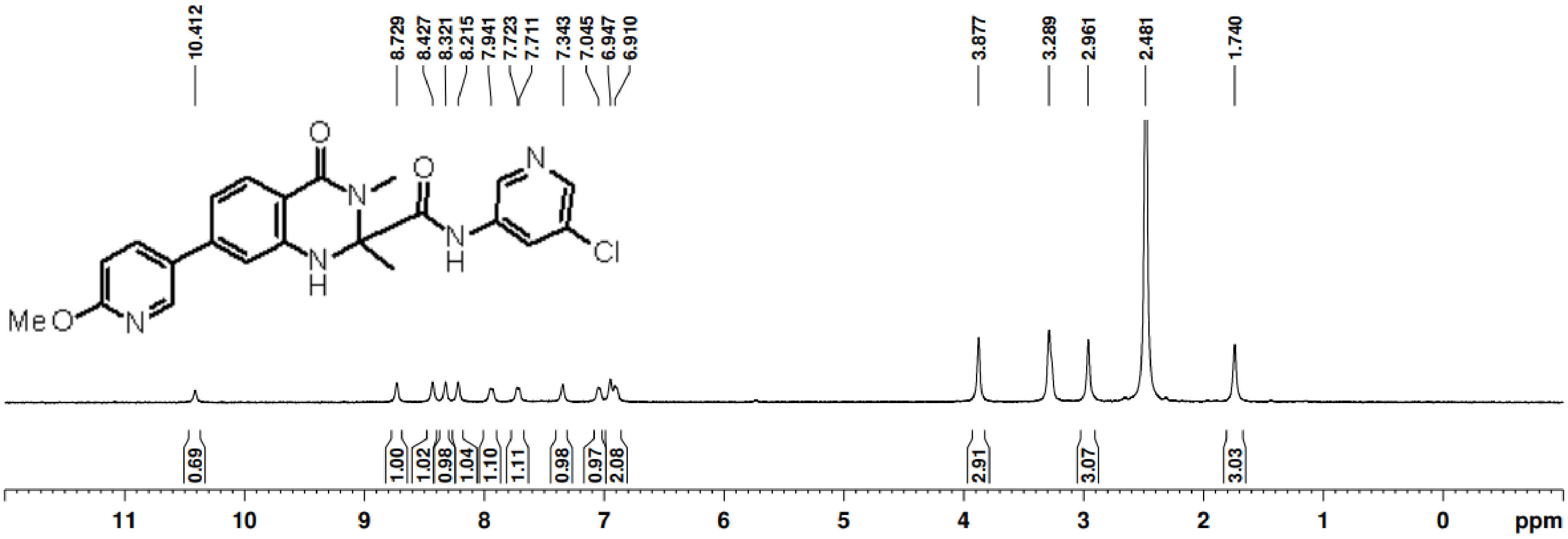

10.412
8.729
8.427
8.321
8.215
7.941
7.723
7.711
7.343
7.045
6.947
6.910
3.877
3.289
2.961
2.481
1.740
MeO
N
H
O
Cl
0.69
1.00
1.02
0.98
1.04
1.10
1.11
0.98
0.97
2.08
2.91
3.07
3.03
11
10
9
8
7
6
5
4
3
2
1
0
ppm

Compound 31

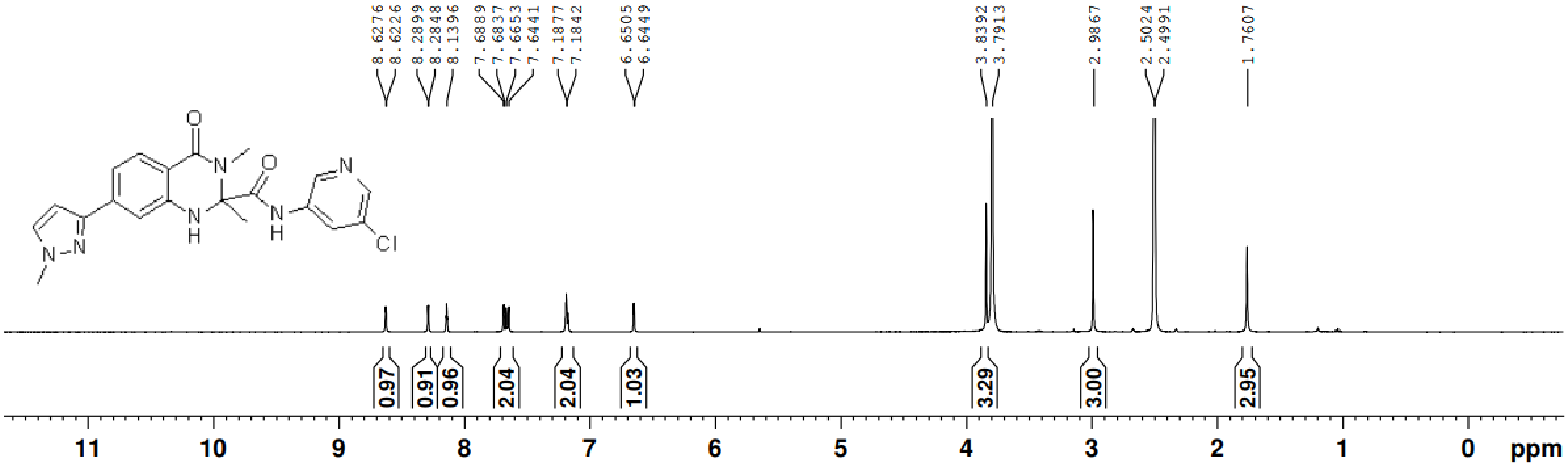
8.6276
8.6226
8.2899
8.2848
8.1396
7.6889
7.6837
7.6653
7.6441
7.1877
7.1842
6.6505
6.6449
3.8392
3.7913
2.9867
2.5024
2.4991
1.7607
0.97
0.91
0.96
2.04
2.04
1.03
3.29
3.00
2.95
11
10
9
8
7
6
5
4
3
2
1
0
ppm

Compound 32

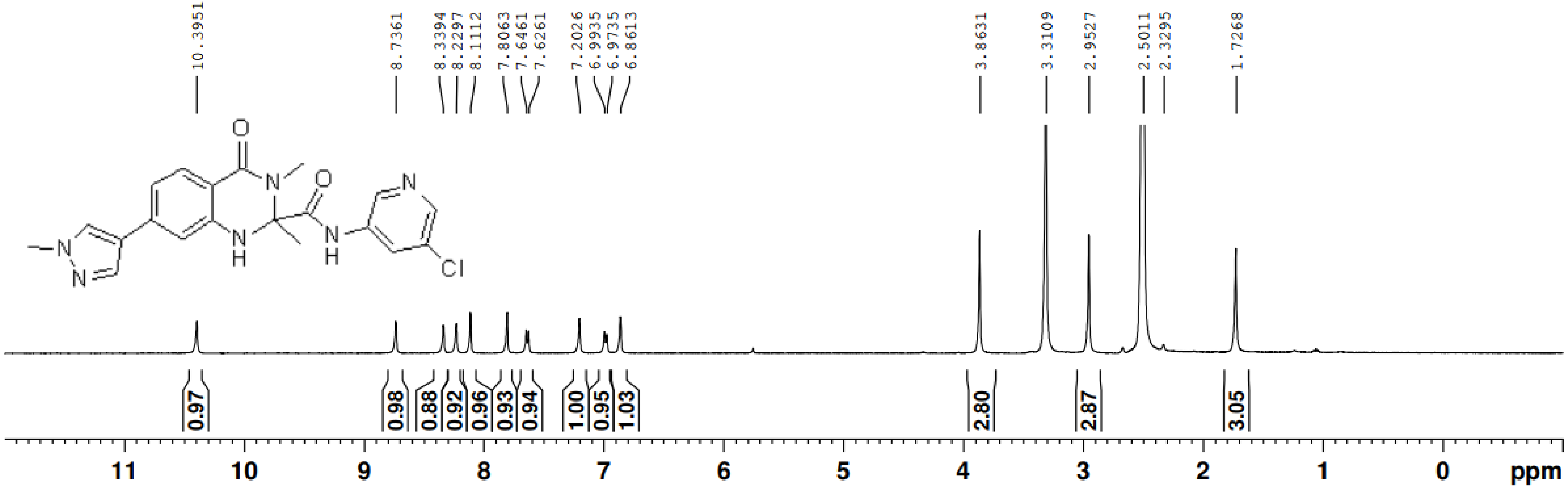

10.3951
8.7361
8.3394
8.2297
8.1112
7.8063
7.6461
7.6261
7.2026
6.9935
6.9735
6.8613
3.8631
3.3109
2.9527
2.5011
2.3295
1.7268
0.97
0.98
0.88
0.92
0.96
0.93
0.94
1.00
0.95
1.03
2.80
2.87
3.05
11
10
9
8
7
6
5
4
3
2
1
0
ppm

Compound 33

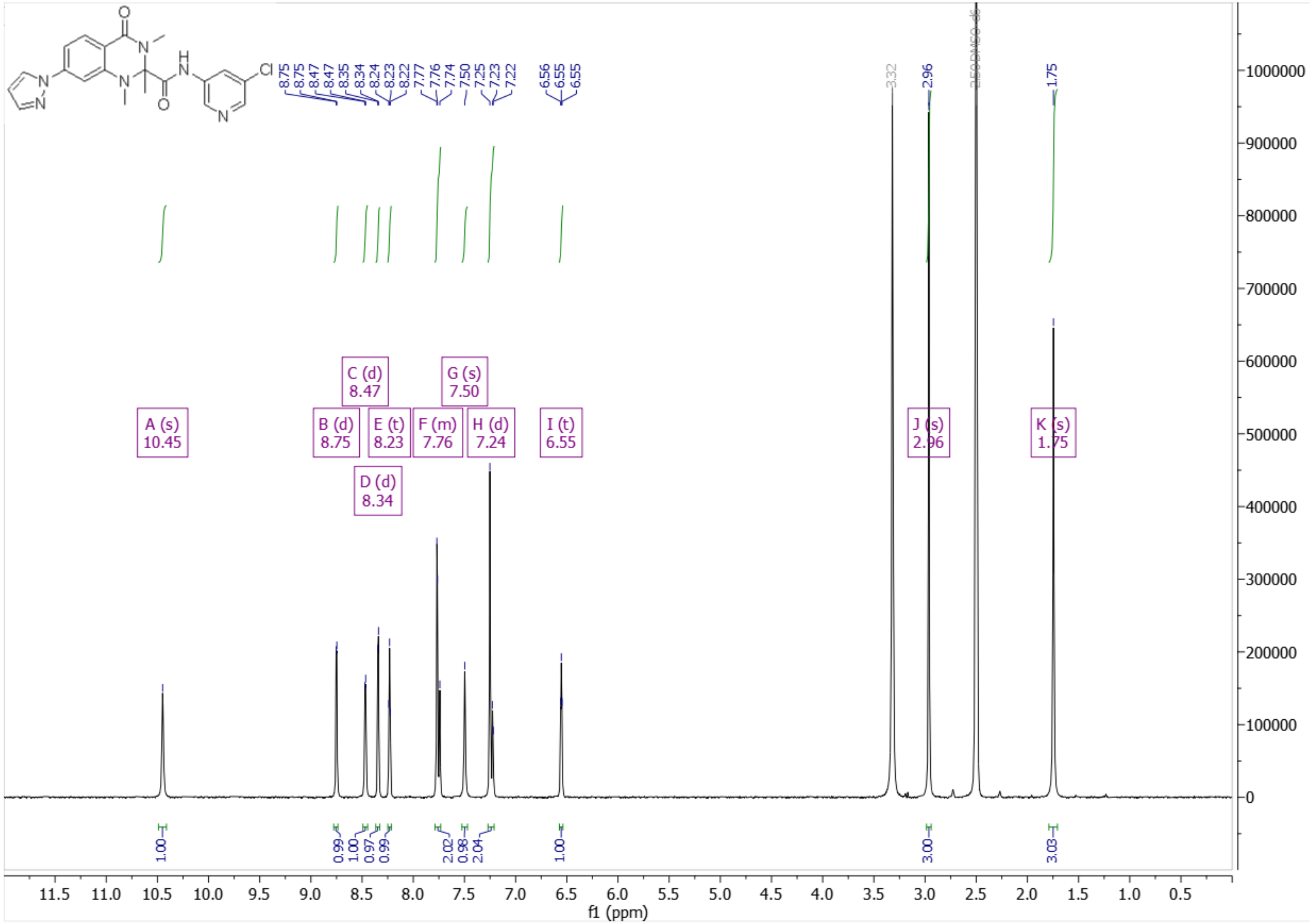

A (s)
10.45
B (d)
8.75
C (d)
8.47
D (d)
8.34
E (t)
8.23
F (m)
7.76
G (s)
7.50
H (d)
7.24
I (t)
6.55
J (s)
2.96
K (s)
1.75
f1 (ppm)

Compound 34

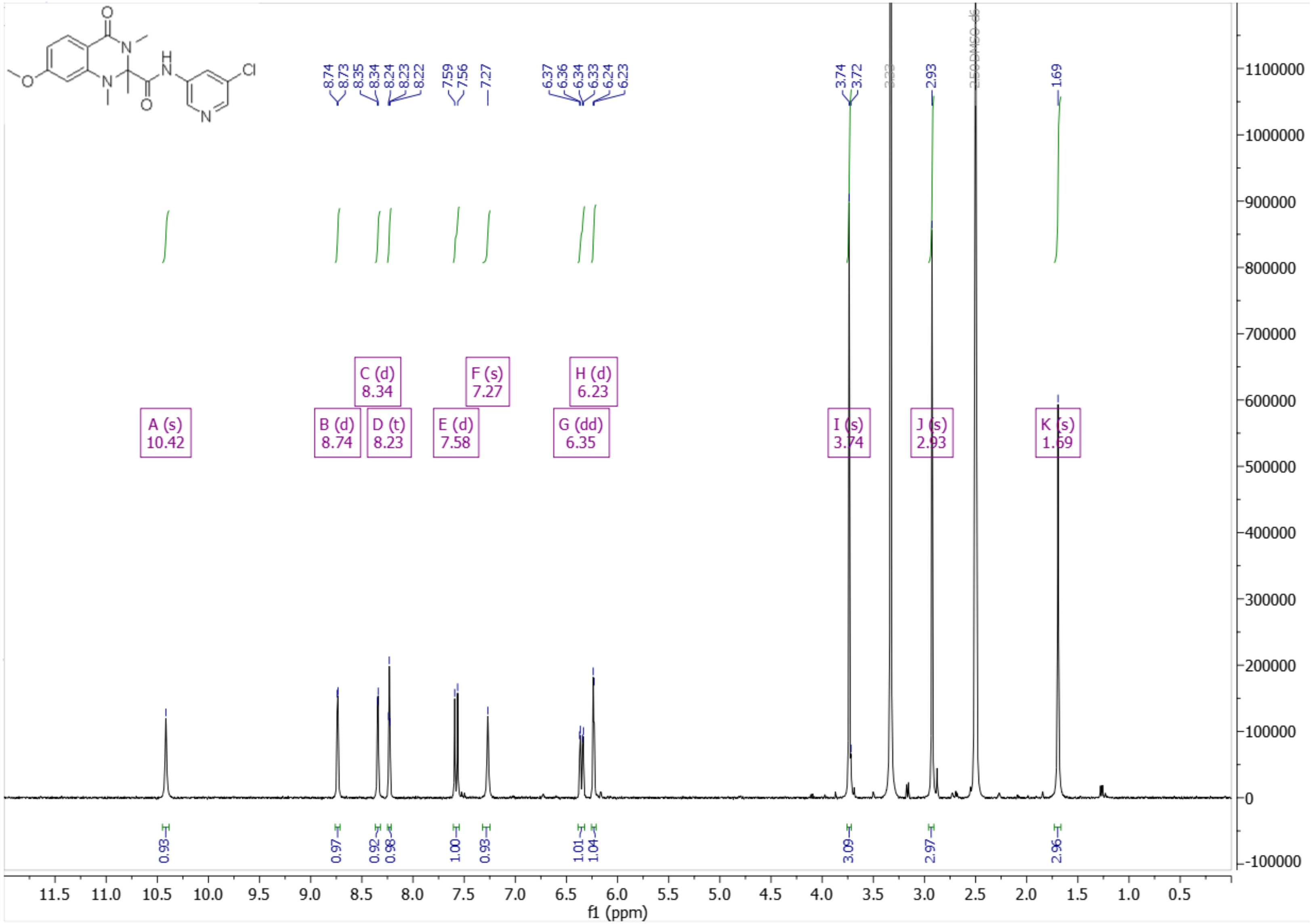

A (s)
10.42
B (d)
8.74
C (d)
8.34
D (t)
8.23
E (d)
7.58
F (s)
7.27
G (dd)
6.35
H (d)
6.23
I (s)
3.74
J (s)
2.93
K (s)
1.69
f1 (ppm)

Compound 35

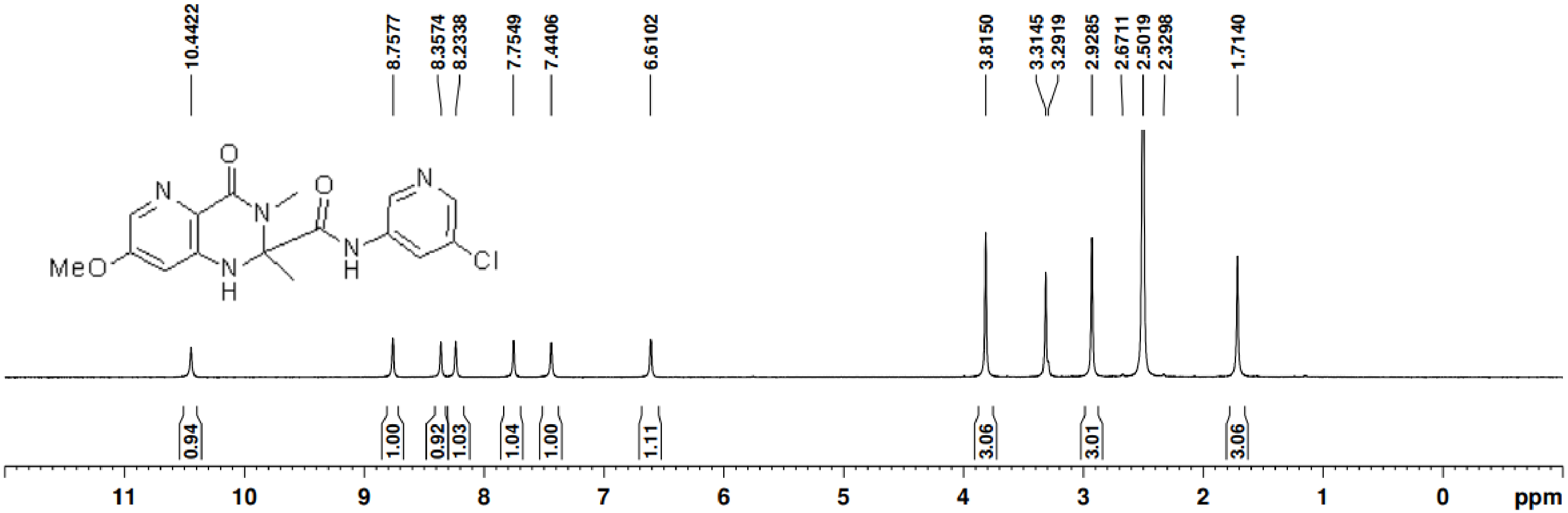
10.4422
8.7577
8.3574
8.2338
7.7549
7.4406
6.6102
3.8150
3.3145
3.2919
2.9285
2.6711
2.5019
2.3298
1.7140
MeO
N
O
N
H
O
N
H
N
Cl
0.94
1.00
0.92
1.03
1.04
1.00
1.11
3.06
3.01
3.06
11
10
9
8
7
6
5
4
3
2
1
0
ppm

Compound 36

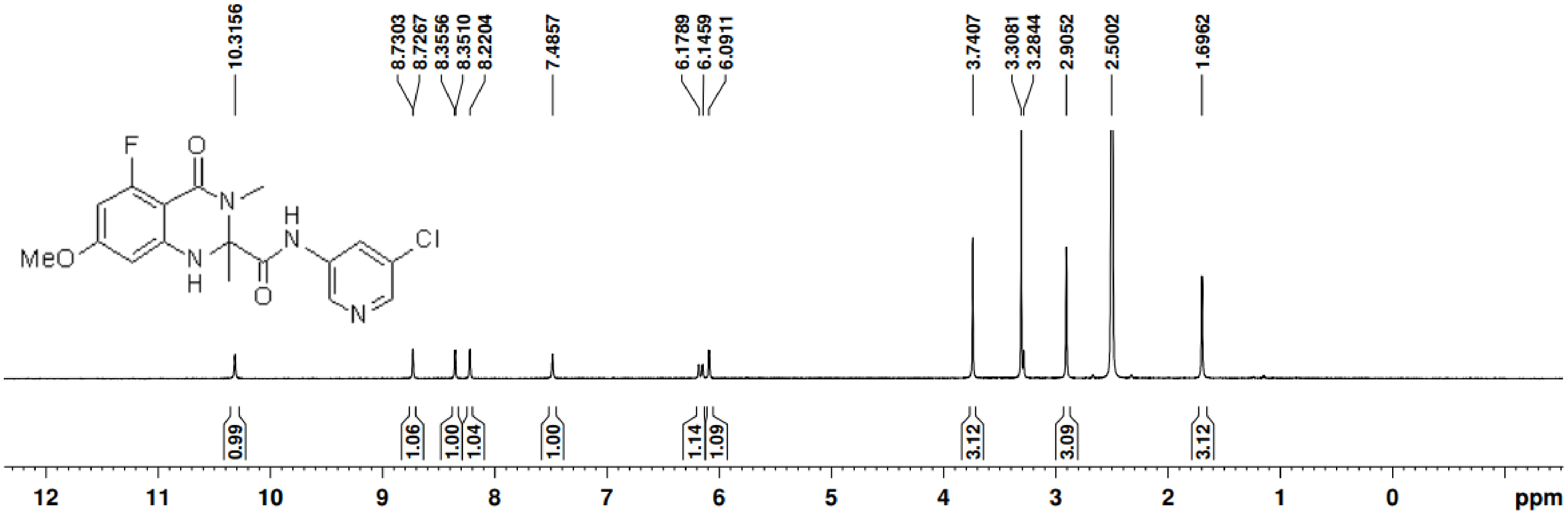
10.3156
8.7303
8.7267
8.3556
8.3510
8.2204
7.4857
6.1789
6.1459
6.0911
3.7407
3.3081
3.2844
2.9052
2.5002
1.6962
F
O
N
H
N
Cl
MeO
N
H
O
N
0.99
1.06
1.00
1.04
1.00
1.14
1.09
3.12
3.09
3.12
12
11
10
9
8
7
6
5
4
3
2
1
0
ppm
